# Graphene‐Inspired Mono‐Elemental 2D Materials: Synthesis, Properties, and Potential Applications of Xenes

**DOI:** 10.1002/advs.202509262

**Published:** 2025-08-24

**Authors:** Yufan Yang, Yuqin Xiao, Qingxin Zhang, Yuxin Yan, Tao Wu, Cheng Heng Pang

**Affiliations:** ^1^ Department of Chemical Engineering University of Nottingham Ningbo China 199 Taikang East Road Ningbo 315100 P. R. China; ^2^ Center for Intelligent and Biomimetic Systems Shenzhen Institutes of Advanced Technology (SIAT) Chinese Academy of Sciences (CAS) Shenzhen 518055 P. R. China; ^3^ State Key Laboratory of Clean Energy Utilization Institute of Carbon Neutrality State Environmental Protection Engineering Center for Coal‐Fired Air Pollution Control Zhejiang University Hangzhou 310027 P. R. China; ^4^ Key Laboratory for Carbonaceous Wastes Processing and Process Intensification Research of Zhejiang Province University of Nottingham Ningbo China Ningbo 315100 P. R. China; ^5^ Municipal Key Laboratory of Clean Energy Conversion Technologies University of Nottingham Ningbo China Ningbo 315100 P. R. China

**Keywords:** Xenes, 2D materials, bottom‐up method, top‐down method, machine learning‐assisted synthesis

## Abstract

Xenes, a class of mono‐elemental two‐dimensional (2D) materials, have emerged as promising candidate materials for next‐generation electronic and energy devices due to their unique structural and electronic properties. This review first systematically categorizes the eighteen experimentally realized Xenes into Group III–VI and other group categories, summarizing their synthesis routes, ranging from top‐down exfoliation to bottom‐up methods. Based on density functional theory (DFT), this paper focuses on theoretical predictions of stable phases and substrate interactions, which guide experimental preparation. Second, functional applications of Xenes in electronics, optoelectronics, catalysis, energy storage, and biomedicine are also reviewed. The impact of atomic configurations on synthesis difficulty, environmental stability, and scalability across different element groups is also discussed. Finally, emerging strategies such as encapsulation, heterostructure design, and machine learning‐guided growth are evaluated to overcome inherent limitations. This paper provides a comprehensive overview of synthesis principles, structure‐property relationships, and stabilization strategies, offering insights into future scalable and robust Xene development directions.

## Graphical abstract

This review summarizes the recent advances in mono‐elemental 2D materials, or Xenes, including their synthesis strategies, theoretical guidance, and stability challenges. It highlights emerging approaches such as deep learning‐assisted design with encapsulation techniques and heterostructure for scalable production and practical applications in electronics, energy, and biomedicine.

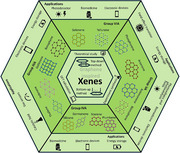

## 1 Introduction

Xenes, a family of monoelemental two‐dimensional (2D) materials composed of elements such as boron,^[^ ^1^ ^]^ silicon,^[^ ^2^ ^]^ phosphorus,^[^ ^3^ ^]^ and bismuth,^[^ ^4^ ^]^ have emerged as promising candidates for next‐generation electronic, optoelectronic, and energy‐related applications. These materials exhibit a variety of atomic arrangements, including planar, curved, and wrinkled geometric structures, and possess unique physicochemical properties such as high carrier mobility, strong spin‐orbit coupling (SOC), and tunable bandgaps.^[^ ^1^, ^5^, ^6^, ^7^, ^8^, ^9^, ^10^ ^]^ The atomic thinness and surface‐dominated nature of Xenes further enhance their potential in fields including catalysis, energy storage, sensing, and nanoelectronics.

While early 2D material research was centered around graphene due to its extraordinary electrical, mechanical, and thermal properties, certain intrinsic limitations have gradually become apparent. The absence of a bandgap renders graphene semi‐metallic, hindering its use in applications that require digital switching, such as field‐effect transistors (FETs).^[^ ^11^, ^12^, ^13^ ^]^ Furthermore, the weak SOC of graphene prevents significant bandgap opening via topological mechanisms, limiting its applicability in spintronics and quantum devices.^[^ ^14^ ^]^ In energy storage, although graphene offers a high theoretical capacity as a lithium‐ion battery anode (up to 744 mAh g^−1^),^[^ ^15^ ^]^ performance enhancements often rely on complex modifications such as heteroatom doping or nanoparticle hybridization, which compromise scalability and structural simplicity.^[^ ^16^ ^]^ These challenges have motivated increasing attention toward Xenes, which retain the dimensional advantages of graphene while offering broader tunability via elemental composition and structural symmetry. For example, borophene and silicene exhibit high theoretical lithium‐ion storage capacity and strong adsorption behavior,^[^ ^15^, ^17^ ^]^ while bismuthene demonstrates an intrinsically opened bandgap suitable for optoelectronic and topological applications.^[^ ^4^ ^]^ The diversity of Xene materials enables geometry‐ and element‐specific tuning of band structures, chemical activity, and interfacial interactions.

Despite these attractive features, the synthesis of Xenes remains a significant challenge. In contrast to van der Waals (vdWs) layered materials, many Xenes do not have stable bulk counterparts and cannot be derived via conventional exfoliation techniques.^[^ ^4^, ^5^ ^]^ Their experimental realization often requires finely tuned growth environments, substrate templating, and kinetic control to stabilize the 2D phase.^[^ ^6^, ^7^, ^8^ ^]^ Moreover, issues such as ambient instability, substrate dependence, and low yield hinder their scalable production and device integration. Overcoming these bottlenecks requires a comprehensive understanding of synthesis mechanisms and stabilization strategies.^[^ ^10^ ^]^ Over the past decade, both top‐down (e.g., chemical exfoliation, plasma etching) and bottom‐up (e.g., molecular beam epitaxy (MBE), chemical vapor deposition) approaches have been developed to fabricate Xenes,^[^ ^1^, ^2^, ^3^, ^4^ ^]^ each offering unique advantages and limitations in crystallinity control, material throughput, and platform compatibility. At the same time, advances in heterostructure engineering, encapsulation, and machine learning‐guided synthesis have opened new avenues for improving material stability and predicting optimal growth conditions.^[^ ^18^, ^19^, ^20^ ^]^ The interdisciplinary nature of Xene research, which encompasses surface science, solid‐state physics, computational modeling, and synthetic chemistry, further highlights the necessity of conducting a comprehensive study on their synthesis and stabilization.

Given the aforementioned progress, a systematic understanding of Xene synthesis strategies and stability enhancement methods is crucial for guiding their scalable preparation and practical integration. This paper reviews the latest advances in Xene synthesis, including the classification of top‐down and bottom‐up approaches and their mechanistic foundations. Representative Xenes are further discussed based on elemental families, with a focus on specific synthesis protocols, structural features, and key application areas. Particular attention is given to current challenges such as the difficulty of exploring synthetic pathways, stability issues in practical applications, and emerging solutions, including heterostructure design, encapsulation techniques, and data‐driven synthesis optimization. By systematically comparing the synthetic routes and stabilization strategies of multiple Xene families, this review has constructed a comprehensive framework, which is expected to stimulate more rational design ideas and provide assistance for the development process of practical applications of Xene‐based materials.

## 2 Synthesis Strategies of Xenes: From Graphene‐Inspired Approaches to Tailored Innovations

**Figure 1:**
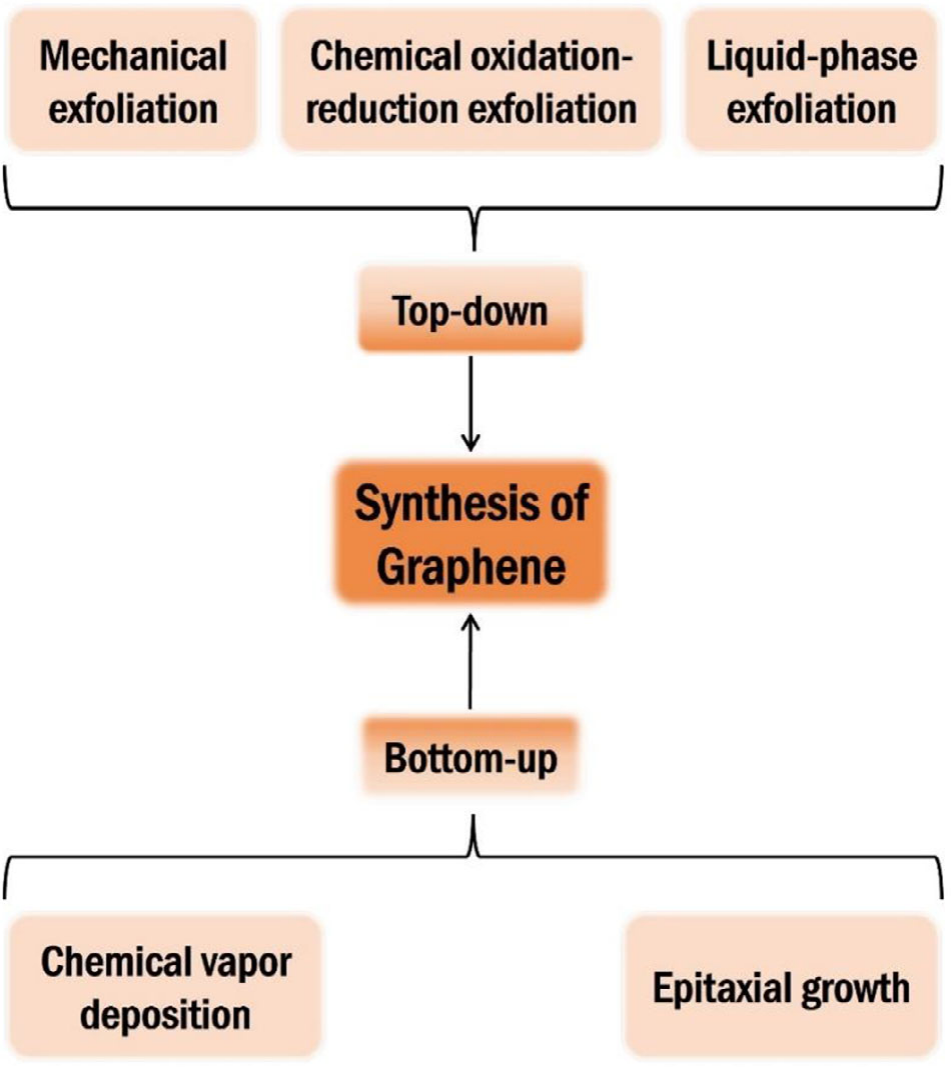
Schematic diagram of Graphene synthesis methods.

The synthesis of Xenes has evolved from graphene‐inspired methods to highly tailored strategies that address the unique structural and chemical demands of various elemental 2D materials. Early breakthroughs in graphene fabrication, such as mechanical exfoliation, liquid‐phase exfoliation (LPE), chemical oxidation‐reduction, and chemical vapor deposition (CVD) as shown in **Figure** **1**, have established the conceptual framework for isolating atomically thin layers and understanding substrate effects.^[^ ^12^, ^21^, ^22^, ^23^ ^]^ However, due to the distinct atomic configurations, buckled geometries, and chemical sensitivities of Xenes, these conventional methods have required substantial adaptations and innovation.

The emerging synthesis techniques for Xenes fall broadly into two categories: top‐down and bottom‐up methods. While both categories build upon the legacy of graphene, they have been significantly extended and refined to meet the specific requirements of individual Xene systems.

### 2.1 Top‐Down Method: Controlled Exfoliation and Chemical Transformation

Top‐down strategies aim to derive monoelemental 2D nanosheets by disassembling bulk or layered precursors through mechanical, chemical, or electrochemical means. While these approaches were originally developed for graphene, such as mechanical exfoliation, chemical oxidation‐reduction, and LPE.^[^ ^12^, ^24^, ^25^, ^26^ ^]^ These methods have been significantly refined and diversified for Xenes to accommodate their diverse bonding structures and reactivity.

In the context of Xenes, top‐down methods no longer rely solely on vdWs interlayer separation, as is the case for graphene. Instead, they increasingly involve chemically guided transformations and selective etching routes. For instance, black phosphorus can be exfoliated into phosphorene through solvent‐assisted sonication under inert conditions to avoid oxidation.^[^ ^1^ ^]^ Tellurene nanosheets have been manually exfoliated and subsequently stabilized in isopropanol (IPA) or N‐methyl‐2‐pyrrolidone (NMP), demonstrating improved dispersion and size uniformity.^[^ ^4^ ^]^ Moreover, layered precursors such as Zintl‐phase compounds and BiTeI‐type structures have enabled electrochemical or chemical transformations to access bismuthene,^[^ ^4^ ^]^ stanene,^[^ ^27^ ^]^ and tellurene^[^ ^28^ ^]^ derivatives. Under certain circumstances, intercalation‐assisted exfoliation is employed to weaken interlayer interactions before shear or sonication exfoliation, which improves both yield and crystallinity.

These innovative routes go beyond the trial‐and‐error exfoliation of graphene and introduce strategies tailored to the intrinsic chemistry of each Xenes system. By refining precursor chemistry, exfoliation environments, and post‐processing techniques, these methods significantly improve the thickness control, lateral size, oxidation resistance, and monodispersity of the resulting nanosheets. Therefore, this concept was initially proposed by the process of graphene exfoliation, and was further developed at the chemical level based on the “top‐down” design philosophy.

### 2.2 Bottom‐Up Method: Epitaxial Precision and Substrate Engineering

Bottom‐up methods construct Xenes atom‐by‐atom or molecule‐by‐molecule, offering precise control over phase, thickness, and crystallographic orientation. These methods originate from techniques used in graphene growth, particularly CVD and epitaxial growth. However, these methods have undergone extensive improvements and optimizations to meet the more complex requirements of Xenes synthesis.

Complementing vacuum‐based techniques, vdWs epitaxy has emerged as a powerful approach. For example, in the case of tellurene, chemical inert layered substrates such as mica can prevent unnecessary hybridization reactions and facilitate the formation of high‐quality crystal structures.^[^ ^29^ ^]^ Moreover, solution‐based bottom‐up strategies such as solvothermal synthesis have enabled the production of large‐area few‐layer tellurene sheets under milder conditions, with lateral sizes exceeding 100 µm and excellent structural integrity.^[^ ^30^ ^]^ Physical vapor transport methods have also been employed for ultrathin bismuthene and tellurene growth using controlled temperature gradients in sealed systems.^[^ ^4^ ^]^ Furthermore, polymorphic Xenes such as borophene have benefited from phase‐selective deposition, where fine‐tuning the substrate lattice, deposition temperature, and post‐annealing profiles can yield distinct β_12_, χ_3_, or striped phases, each with different electronic and mechanical properties.^[^ ^1^ ^]^ When these advanced deposition techniques are combined, they surpass the bottom‐up technical performance of graphene and enable the meticulous design of functional polymorphism, anisotropic properties, and integrable forms.

## 3 Xenes: Synthesis, Properties, and Potential Applications

**Figure 2:**
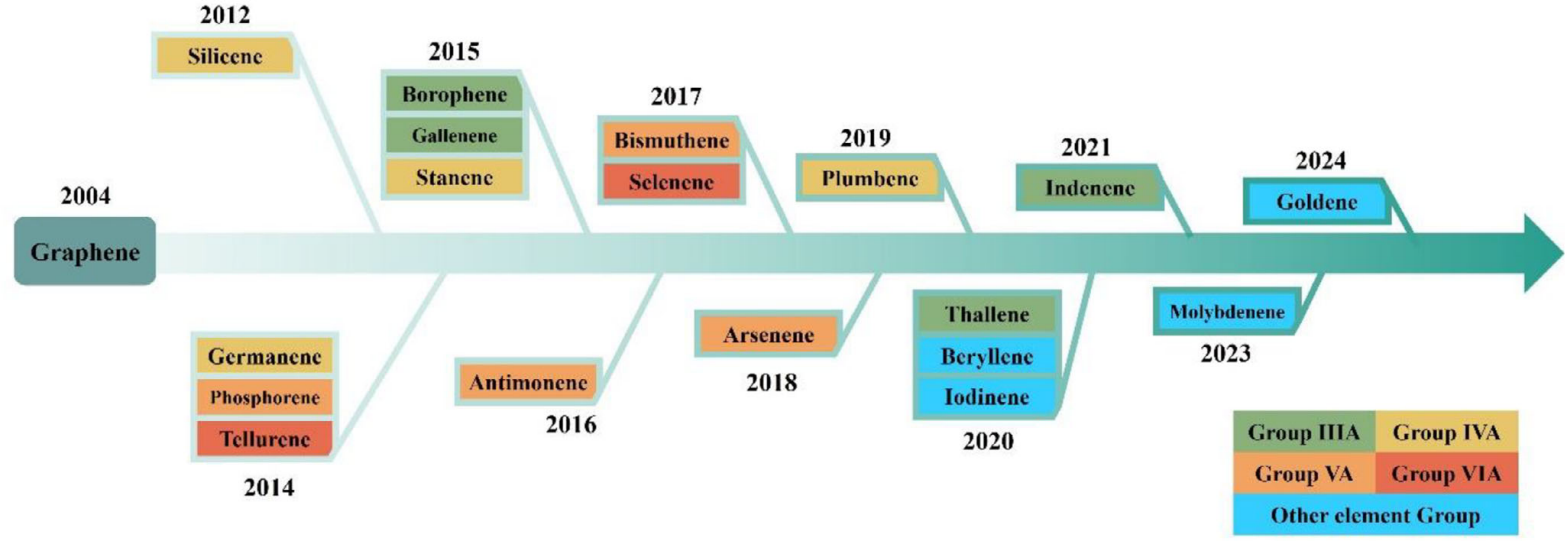
Timeline for the success of Xenes experimental synthesis.

Inspired by the discovery and synthesis of graphene, the concept of Xenes is proposed and subsequently realized experimentally. Many of the fabrication techniques developed for Xenes have been adapted from the graphene synthesis method. These fabrication methods can mainly be divided into the following two categories: top‐down method (e.g., mechanical exfoliation and LPE) and bottom‐up method (e.g., physical vapor deposition (PVD), CVD, and epitaxial growth). Unlike graphene which currently exists as monolayer nanosheets, most 2D‐Xenes are thermodynamically unstable at freestanding, prone to collapse or reconstitution into three‐dimensional structures, and require a suitable substrate to provide physical support in order to maintain their structural integrity.^[^ ^31^, ^32^ ^]^ At present, eighteen types of Xenes have been successfully synthesized, and the research on them is gradually deepening. **Figure** **2** shows the timeline of all the Xenes that have been experimentally synthesized so far. The concept of 2D allotrope silicon was first proposed in 1994,^[^ ^33^ ^]^ and this material was later named “silicene” in analogy to graphene in a subsequent study.^[^ ^34^ ^]^ The boron, an element adjacent to carbon in the periodic table, was proposed by Mannix et al. to prepare atomically thin borophene.^[^ ^1^ ^]^ In addition, the group VIA element selenium, which exists in theory, has also been successfully synthesized by the PVD method, and large 2D selenium nanosheets have been obtained.^[^ ^35^ ^]^

Xenes exhibit numerous commendable characteristics. First, Xenes have the same 2D nanostructure as graphene, which gives them a large specific surface area. The large surface area also leaves their surface atoms exposed, giving them better atomic utilization. In addition, Xenes possess excellent photoelectric performance, including tunable bandgaps and high carrier mobility. These characteristics enable the broad‐spectrum light absorption to cover the ultraviolet and infrared regions, thereby generating superior light collection efficiency. Due to these characteristics, Xenes have great prospects in the field of photocatalysis, where efficient solar energy conversion is indispensable.^[^ ^36^ ^]^ At the same time, due to their single chemical composition, Xenes are easier to be regulated and applied to defect engineering and other fields.^[^ ^37^ ^]^ A variety of Xenes have only been synthesized in recent years, so there are few literatures that review their current research status. This article will review the Xenes that have been successfully synthesized, including their preparation methods, properties, and potential applications. In this review, these Xenes will be discussed based on the differences in the groups of elements in their periodic tables.

### 3.1 Group IIIA

In group IIIA, the 2D forms of four elements have been successfully prepared, including borophene, gallenene, indenene, and thallene. A preliminary introduction to their synthesis methods and morphology is statistically presented in **Table** **1**.

**Table 1:** Xenes of Group IIIA elements.

Xenes	Precursor	Synthetic method	Morphological characteristics	Refs.
Borophene	Pure boron	MBE on Ag (111)	Anisotropic striped phases, with periodicity in *a* direction of 0.51 nm and *b* direction of 0.29 nm	[1]
	Pure boron	MBE on Al (111)	Hexagonal lattice structure, planar and non‐wrinkled surface	[48]
	Pure boron	MBE on Ir (111)	Hexagonal lattice arrangement with (6 × 2) superstructure, rotational domain	[49]
	NaBH4	CVD, H_2_ as carrier gas	With thicknesses of 3.4 nm, large coverage area	[50]
	Boron powder	LPE with sonochemical	Single or multi‐layer structure, lateral dimension achieves 1.2 microns	[54]
	Boron powder	Electrochemical exfoliation	Anisotropic crystal structure, good stability, with dimensions ranging from 400 to 600 nm	[55]
Gallenene	Ga	Solid‐melt exfoliation (Si/SiO_2_)	Dynamic and stable metallic properties	[9]
	Ga	MBE on GaN (0001)	Bialyer gallenene film, hexagonal structure with a thickness of 0.552 nm	[56]
Indenene	In	MBE on SiC (0001)	Triangular lattice structure, honeycomb connectivity	[57]
Thallene	Tl	MBE on Si (111)	√3 × √3‐R30° superstructure with honeycomb geometry, planar surface	[58]
	Tl	MBE like epitaxial on NiSi_2_/Si(111)	Non‐buckled structure, high‐crystallinity monolayer film	[59]

#### 3.1.1 Borophene

##### Synthesis—Bottom‐Up Method

**Figure 3:**
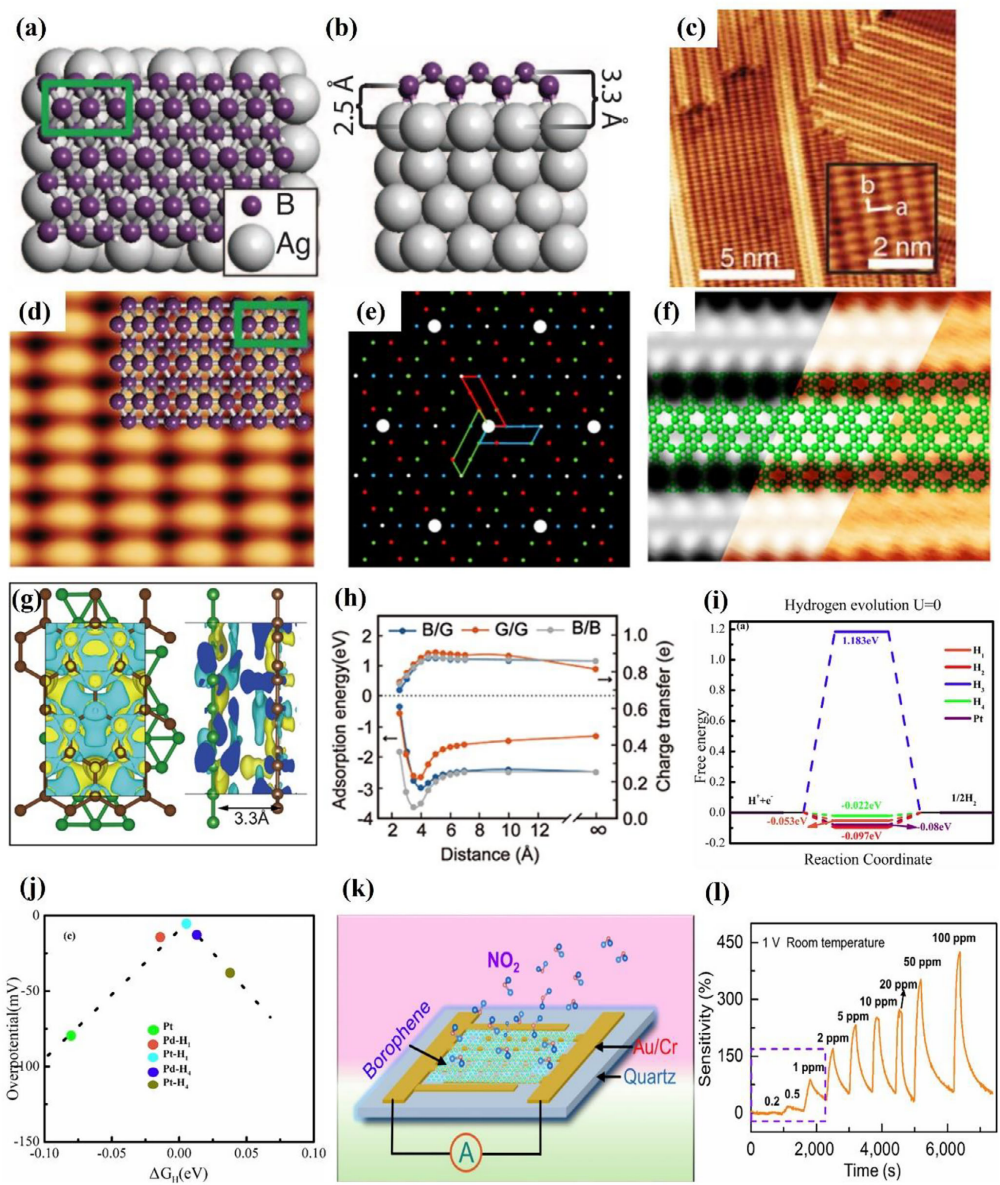
Borophene: from atomic structure to device applications. a) Top and b) side views of simulated borophene on Ag(111), with the unit cell marked in green. c) STM image showing the striped‐phase atomic structure; inset highlights the rectangular lattice with lattice vectors. d) Simulated STM image of empty states overlaid with atomic positions; unit cell indicated in green. Reproduced with permission.^[^ ^1^ ^]^ Copyright 2015, American Association for the Advancement of Science AAAS. e) Simulated hexagonal lattice of χ_6_‐borophene with (6 × 2) superstructure and visible rotational domains. f) Left: Simulated tunneling current isosurface of the χ_6_ structure; Right: Corresponding region from experimental STM imaging; Center: Proposed atomic model superimposed to illustrate agreement between simulated and observed lattice features. Reproduced with permission.^[^ ^49^ ^]^ Copyright 2019, American Chemical Society publications. g) Top and side views of the deformation charge density of B/G; h) Adsorption energies and charge transfer of Li in the interlayers of B/G, bilayer graphene, and bilayer borophene as a function of interlayer distance. Reproduced with permission.^[^ ^51^ ^]^ Copyright 2020, American Chemical Society. i) Step diagram of hydrogen adsorption Gibbs free energy (Δ*G*_H*_) for the χ^3^/TM@WS_2_ heterojunction; j) Volcano plot of HER overpotential for χ^3^/TM@WS_2_ heterojunctions as a function of ΔG_H*_. Reproduced with permission.^[^ ^52^ ^]^ Copyright 2025, American Chemical Society. j) Device schematic of the sensor structure; k) Sensing response transients of borophene to varying concentrations of NO_2_. Reproduced with permission.^[^ ^53^ ^]^ Copyright 2021, Springer Nature.

As the first element of Group IIIA, and adjacent to carbon in the periodic table of elements, boron is a promising candidate for 2D materials. However, since boron has only three valence electrons, it cannot form a stable honeycomb structure like carbon in graphene. As early as 1997, theoretical calculations of the geometry of boron clusters were reported. Based on ab initio quantum‐chemical methods, the Aufbau principle was proposed to construct highly stable triangular lattice boron species structures.^[^ ^38^ ^]^ Subsequent studies have also confirmed that boron clusters have planar or quasi‐planar structures.^[^ ^39^, ^40^, ^41^, ^42^ ^]^ The theoretical study of 2D boron sheets has been carried out, and several structures have been predicted, such as α‐sheets,^[^ ^43^, ^44^ ^]^ β‐sheets,^[^ ^45^ ^]^ and χ‐sheets.^[^ ^46^ ^]^ Through experimental characterization, Liu et al. provided preliminary evidence of the production of layered structure of boron nanotubes in 2010.^[^ ^47^ ^]^ However, 2D boron sheets (borophene) were not experimentally obtained until 2015. Mannix and his colleagues obtained atomic‐scale borophene by MBE method under ultra‐high vacuum (UHV) conditions using a solid boron atom source of 99.9999% purity as a precursor and Ag (111) as a substrate. The simulated structure of borophene on Ag (111) substrate is shown in **Figure** **3**a,b.^[^ ^1^ ^]^ This structure is consistent with the borophene structure of earlier theoretical studies. The scanning tunneling microscope (STM) topography image of the sample (Figure 3c) shows borophene with obvious anisotropic striped phases. Specifically, the periodicity in the *a* direction is 0.51 nm and that in the *b* direction is 0.29 nm. The atomic structure of the boron sheet in the simulated STM rectangular lattice and parallel fringe mode (Figure 3d), verified that the experimental synthetic material was borophene.^[^ ^1^ ^]^ In 2018, the researchers successfully prepared borophene on the surface of Al (111) following the previously proposed method.^[^ ^48^ ^]^ This borophene existed in the form of a planer hexagonal lattice structure with the lattice constant of 2.9 Å. In the following year, the epitaxial growth of borophene on an Ir (111) substrate was also reported, and its lattice structure was characterized by a hexagonal lattice arrangement with (6 × 2) superstructure and rotational domains (Figure 3e).^[^ ^49^ ^]^ The author compared the molecular model optimized via DFT with the high‐resolution STM (HR‐STM) images, as shown in Figure 3f. The strong agreement between simulation and experimental results provides direct evidence supporting the atomic configuration of the synthesized borophene.^[^ ^49^ ^]^ Further, CVD has been successfully applied. The method used sodium borohydride (NaBH_4_) powder as a boron source and quartz as a substrate. The researcher used hydrogen as the carrier gas, and the reaction was taken under 490 °C and 650 °C respectively for half an hour to produce borophene.^[^ ^50^ ^]^ The synthesized borophene film has a thickness of 3.4 nm and a large coverage area on the substrate.

##### Synthesis—Top‐Down Method

With further study of borophene, other synthesis methods have been reported. Since boron does not exist in a layered structure, such methods often require the application of additional steps. In 2019, Ranjan et al. innovatively proposed a top‐down freestanding borophene synthesis method based on the principle of LPE combined with sonochemical.^[^ ^54^ ^]^ Freestanding borophene sheets were synthesized via sonochemical exfoliation of boron powder in polar solvents such as acetone. The borophene synthesized by this method has a thickness ranging from single layer to multiple layers, and the transverse dimension can reach 1.2 microns. Inspired by the method of synthesizing graphene, a method based on electrochemical exfoliation has also been proposed by Chowdhury et al. .^[^ ^55^ ^]^ This method took advantage of the characteristic that the electrical conductivity of boron changes with the change of temperature. Boron, which had similar electrical conductivity to metal under high temperature,^[^ ^60^ ^]^ was used as the cathode, and platinum was used as the anode. The boron sheets were collected in the electrolyte solution (ionic liquid, aqueous inorganic salt, etc.). Then, the collected boron sheets were transferred to acetone, and after ultrasonic and centrifugal treatment, the supernatant was collected and dried to obtain borophene. The product exhibits anisotropic crystal structure and good stability, with a size range of 400 to 600 nm.

##### Properties and Potential Applications of Borophene—Energy Storage

The high theoretical capacity, rapid ion diffusion, and low energy barrier of borophene make it have great application potential in energy storage fields such as batteries and hydrogen storage. For example, lithium ion batteries (LiBs), with the high energy efficiency and environmentally friendly sustainability, has become the current energy storage choice for mobile electronic devices and electric vehicles.^[^ ^61^ ^]^ As a typical representative of 2D materials, graphene has achieved great success in LiBs^[^ ^62^ ^]^ because of its superior electrical conductivity and large specific surface area.^[^ ^63^, ^64^ ^]^ However, its high diffusion barrier (≈0.3 eV) leads to the problem of slow charging and discharging of the battery.^[^ ^8^ ^]^ The emergence of borophene is expected to provide important conditions for breakthroughs in LiBs. Borophene has an excellent adsorption effect on lithium ions. Both Zhao et al.^[^ ^65^ ^]^ and Yang et al.^[^ ^62^ ^]^ simulated the adsorption and diffusion behavior of lithium ions on the surface of borophene based on the DFT calculations. The diffusion barrier of Li simulated by Yang et al. on borophene on the flat can be as low as 0.007 eV, which can be negligible. Zhao's group simulated a fully lithiated borophene (a boron combined with 0.75 lithium) with a theoretical capacity of 1860 mAh g^−1^, which is much larger than graphene (744 mAh g^−1^).^[^ ^15^ ^]^ Borophene showed potential as an anode material for LiBs with a low diffusion energy barrier of 3 meV and a strong adsorption energy of 2.92 eV.^[^ ^62^, ^66^ ^]^ Research on the Li modification of borophene^[^ ^65^ ^]^ has led to more exploration. Er et al. found that the hydrogen storage capacity of borophene is significantly improved after the absorption of alkali metal elements such as Li, Na, and K.^[^ ^67^ ^]^ The hydrogen storage capacity of borophene after Li‐decoration can reach 10.7 wt% molecular hydrogen with the average energy of 0.15 eV/H_2_. The results indicate that alkaline metal‐modified borophene has excellent application prospects for hydrogen storage. One study also used few‐layer borophene as the electrode material in supercapacitors. This capacitor exhibits a wide potential window of 3.0 V and a power density of up to 478.5 W kg^−1^. After 6,000 cycles, it still retained 88.7% of its initial capacitance, indicating excellent cycling stability.^[^ ^68^ ^]^ At the same time, the construction of 2D–2D heterostructures such as borophene/graphene (B/G) has proven effective in enhancing the structural stability of borophene while retaining its remarkable electrochemical properties. As shown in Figure 3g, pronounced interfacial charge redistribution occurs, with electrons transferring from graphene to borophene, forming a stable vdWs interface without disrupting the intrinsic metallicity.^[^ ^51^ ^]^ Moreover, Figure 3h demonstrates that lithium atoms preferentially adsorb in the interlayer region, with a minimum adsorption energy of −2.959 eV at a spacing of 4 Å and maximum charge transfer around 4.5 Å. These findings not only highlight the tunability of interlayer interactions in heterostructures, but also underscore the synergistic effect of combining two 2D materials, resulting in enhanced Li binding and charge transport performance.^[^ ^51^ ^]^ Borophene shows great promise in energy storage field due to its ultralow ion diffusion barriers, high theoretical capacities, and strong adsorption energies. Its metallic conductivity and flexible structure enable rapid charge transport and interfacial tunability, especially in heterostructures like B/G composites. Compared to graphene, borophene offers faster kinetics and higher storage capacity. However, challenges such as environmental instability, synthesis scalability, and mechanical fragility hinder its practical deployment. Addressing these through surface engineering and composite design is key to realizing its application potential.

##### Properties and Potential Applications of Borophene—Catalytic Field

In the catalytic field, the high surface‐to‐volume ratio and electronic tunability of borophene render it a promising candidate for hydrogen evolution reactions (HER). DFT calculations have revealed that both α‐ and β_12_‐phase borophene possess nearly ideal hydrogen adsorption free energy (ΔG_H*_) close to zero, particularly at low‐coordination sites around hexagonal holes, achieving ΔG_H*_ ≈ 0.00 – 0.04 eV, comparable to or surpassing that of Pt.^[^ ^69^ ^]^ Although borophene synthesized via MBE adheres strongly to metallic substrates, computational studies indicate that Ag(111) not only stabilizes the boron lattice but can also modulate its electronic states to preserve or even improve HER activity, while substrates like Cu(111) cause excessive electron transfer and performance deterioration. To further optimize HER performance and overcome borophene's intrinsic air instability and limited H‐trapping capability due to its inert basal plane, Yang et al. proposed a vdWs heterojunction structure by integrating χ^3^‐type borophene with 2H‐WS_2_.^[^ ^52^ ^]^ This heterojunction exhibited excellent catalytic performance with ΔG_H*_ values as low as −0.022 eV (H4 site) and −0.053 eV (H1 site) as shown in Figure 3i, benefiting from enhanced charge redistribution and metallic behavior near the Fermi level induced by interlayer coupling. Remarkably, upon doping noble metal atoms (Pt or Pd) into either the borophene or WS_2_ layer, the system demonstrated further reduction of ΔG_H*_ to near‐zero (0.005 eV for Pt@WS_2_ configuration), placing the system near the top of the HER volcano plot (Figure 3j). These heterostructures not only improve catalytic kinetics but also enhance structural stability by anchoring reactive borophene onto more chemically inert substrates. The high surface area of borophene 2D nanostructure also enables it to have a large number of active sites. For example, borophene can be used as a catalyst in HER with near‐zero free energy.^[^ ^69^ ^]^ Considering that the borophene synthesized by the MBE method is difficult to separate from the substrate, the catalytic performance of borophene/Ag (111) in the HER is also calculated. The results show that the substrate has no negative effect on the catalytic effect.

##### Properties and Potential Applications of Borophene—Gas Sensors

Previous studies have demonstrated the potential of borophene in gas sensors, owing to its high carrier mobility.^[^ ^54^ ^]^ A borophene‐based NO_2_ sensor was successfully fabricated, demonstrating outstanding performance in terms of sensitivity, selectivity, and response dynamics at room temperature. As schematically illustrated in Figure 3k, the sensor architecture consists of ultrathin borophene sheets deposited on a quartz substrate with interdigital Cr/Au electrodes, enabling reliable electrical readout upon gas exposure. The use of high‐quality α‐phase borophene with well‐defined lattice structures ensures effective charge transfer and robust surface activity essential for gas adsorption. Notably, the sensor exhibits an exceptionally low detection limit of 0.2 ppm, outperforming conventional 2D material‐based sensors such as those using graphene, MoS_2_, or phosphorene under the same room temperature conditions. As shown in Figure 3l, the borophene sensor exhibits a rapid, concentration‐dependent electrical response toward NO_2_ in the range of 0.2–100 ppm, with a response time of ∼30 s and recovery time of ∼200 s.^[^ ^53^ ^]^ The use of 2D semiconductor materials (MoS_2_, WS_2_) are the substrate of the β_1_‐borophene sheet has improved the thermal stability and enhanced the gas sensing performance.^[^ ^45^ ^]^ Even under significant bending angles (0°, 45°, and 90°), the device sustains nearly unchanged signal intensity when exposed to 2 ppm NO_2_, demonstrating its mechanical resilience and functional stability. Compared with common graphene, MoS_2_, or reduced graphene oxide sheets sensors, borophene‐based sensors have broad prospects for higher sensitivity and faster reaction rates.^[^ ^70^ ^]^ Borophene shows great promise for gas sensing due to its high carrier mobility, active surface, and flexibility. It achieves ultra‐low NO_2_ detection limits with fast response at room temperature, outperforming graphene and MoS_2_‐based sensors. Its performance remains stable under mechanical deformation. However, challenges such as environmental instability, oxidation, and scalable fabrication limit practical use. Structural engineering and encapsulation are needed to enable reliable, flexible sensing applications.

#### 3.1.2 Gallenene

##### Synthesis—Bottom‐Up Method

**Figure 4:**
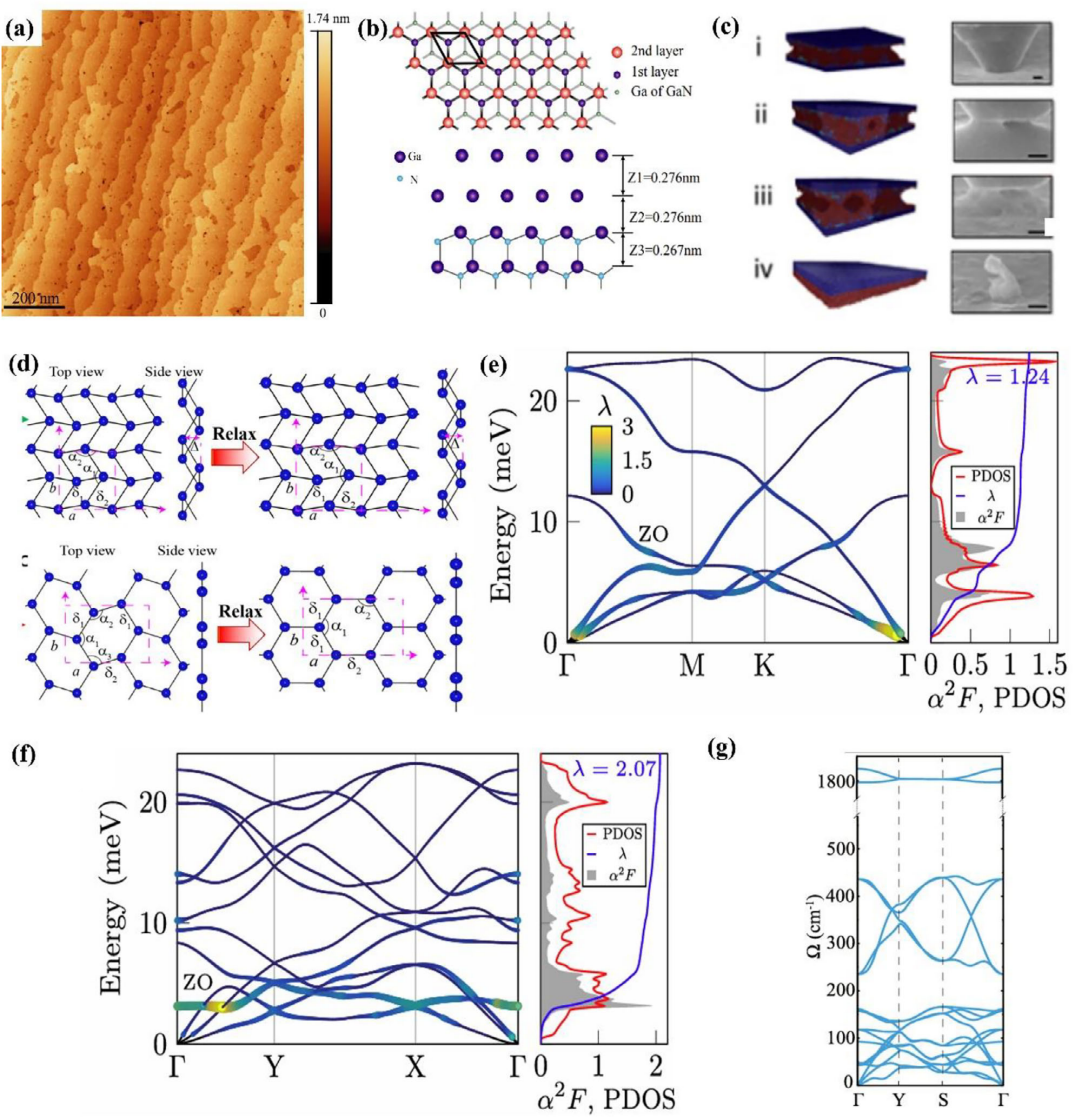
Atomic configurations and phonon characteristics of Gallenene. a) Topographic image of bilayer gallium film; b) Top and profile structure diagram of Ga/GaN, where Z is the average separations between each layer; Reproduced with permission.^[^ ^56^ ^]^ Copyright 2015, APS. c) Diamond flat punch surface image (i–iii is void change, iv is a monolayer of Ga); d) Schematic diagram of two gallenene structures where the top one is Ga (010) and bottom one is Ga (100). Reproduced with permission.^[^ ^9^ ^]^ Copyright 2018, American Association for the Advancement of Science. Phonon dispersion, phonon DOS, Eliashberg function α^2^F(ω), and electron–phonon coupling strength λ(ω) of e) Ga (100) and f) Ga (010). Reproduced with permission.^[^ ^73^ ^]^ Copyright 2021, IOP Publishing. g) Phonon dispersion of w‐gallenene. Reproduced with permission.^[^ ^74^ ^]^ Copyright 2018, American Chemical Society.

Gallium is in the IIIA group of the fourth period in the periodic table of elements. Interestingly, while most other 2D materials, such as graphene, are linked by vdWs forces between adjacent layers.^[^ ^71^ ^]^ Gallium is a quasi‐layered material that connects adjacent 2D planes through covalent bonds.^[^ ^72^ ^]^ Compared with other 2D materials, the covalent interaction in gallenene helps enhance its thermal stability, but this also makes gallenene difficult to obtain through direct top‐down exfoliation. The 2D gallium film was prepared by Zhang et al.^[^ ^56^ ^]^ in 2015. They used a gallium source with a purity of up to 99.995% to obtain Ga films on an Al_2_O_3_ substrate by epitaxial growth method by metal‐organic CVD. GaN with a thickness of 3 microns was deposited on Al_2_O_3_ for the growth of Ga films, and there was a 25 nm AlN buffer layer between the substrate and Al_2_O_3_. Bilayer gallium films were obtained by this method. The morphology of the atomic plane Ga film is shown in **Figure** **4**a. The bilayer Ga atomic film has a hexagonal structure (Figure 4b), with a thickness of approximately 0.552 nm.

##### Synthesis—Top‐Down Method

However, the top‐down approach has been explored as the research deepens. The monolayer and few‐layer Ga films are exfoliated from the molten Ga solid surface in 2018.^[^ ^9^ ^]^ This method takes advantage of the fact that the high thermal vibration of metals leads to a decrease in their strength at temperatures near the melting point. At the interface between the solid metal substrate and the liquid metal, temperature differences can induce heterogeneous nucleation, leading to the growth of solid crystalline layers on top of the liquid metal. Due to the difference in strength, the force required to separate the solid crystals from the surface will be greatly reduced. The method exploits this feature and names it the ‘solid‐melt exfoliation technique’. Specifically, this group placed liquid Ga (50 °C) onto a Si/SiO_2_ substrate and cooled it to room temperature (30 °C) under UHV conditions. Strain is applied with a diamond flat punch to form a void, and a monolayer of Ga is finally separated (Figure 4c). The stress required to separate the Ga monolayer is 1.5 MPa. Interestingly, the report also successfully found the predicted lattice orientations of two different gallenene films. As shown in Figure 4d, a quasi‐2D multilayer structure is formed by exfoliation along the direction of α‐Ga (010). In contrast, the single layer of gallium atoms obtained by exfoliation along the direction of α‐Ga (100) forms a honeycomb structure after relaxation.

##### Properties and Potential Applications Of Gallenene

Although the number of studies on the properties of gallenene is limited till nowadays, the unique physical properties of gallenene make it an exciting 2D material. 2D gallenene represents metallic properties. Gallenene obtained by solid‐melt exfoliation has two distinct lattice planes: Ga (100) and Ga (010). Gallenene materials in both structures have excellent electrical conductivity, very high electrical conductivity, and even 2D superconductivity.^[^ ^9^ ^]^ The stable structure of gallenene enables it to be transferred to any substrate in principle, while maintaining its structure and electronic properties while minimizing the electronic property deviation caused by hybridization. For instance, Ga (100) has good structural stability on metal (Al, Ag, and Ni) and ceramic (GaN) substrates and retains excellent electronic properties. The study of the structure of two gallenene by DFT calculations shows that the phonon band structure of their relaxed structure only has a biaxial tensile strain of 5% and 2%, respectively.^[^ ^73^ ^]^ Their strain‐stabilized phonon dispersions show no imaginary frequencies (Figure 4e,f), verifying their robustness for substrate integration. The Fermi surface forming principle of Ga (100) and Ga (010) is different, resulting in their different density of states at the Fermi level (Ga (100) is 0.91 eV and Ga (010) is 0.43 eV per atom). Moreover, the coupling of low‐energy phonon modes in both gallenene structures is extraordinarily strong, especially in Ga‐010. The electron‐phonon coupling of both two structures is sufficient to induce superconductivity. Another study showed that gallenene with hydrogen addition (known as “gallenane”) has stronger electron‐phonon coupling.^[^ ^74^ ^]^ In particular, the hydrogenation both one‐sided and two‐sided suppresses dynamical instabilities and significantly increases in‐plane stiffness and work function. As shown in Figure 4g, the phonon band structure of one‐sided hydrogenated w‐gallenene becomes fully stable without external strain, indicating the effectiveness of hydrogenation in reinforcing lattice dynamics. The stability of gallenene under high strain, easy of transferring from substrates and superconducting properties make gallenene a promising cornerstone for advancing the fundamental discipline of nano mechanics in the future.

#### 3.1.3 Indenene

##### Synthesis—Bottom‐Up Method

**Figure 5:**
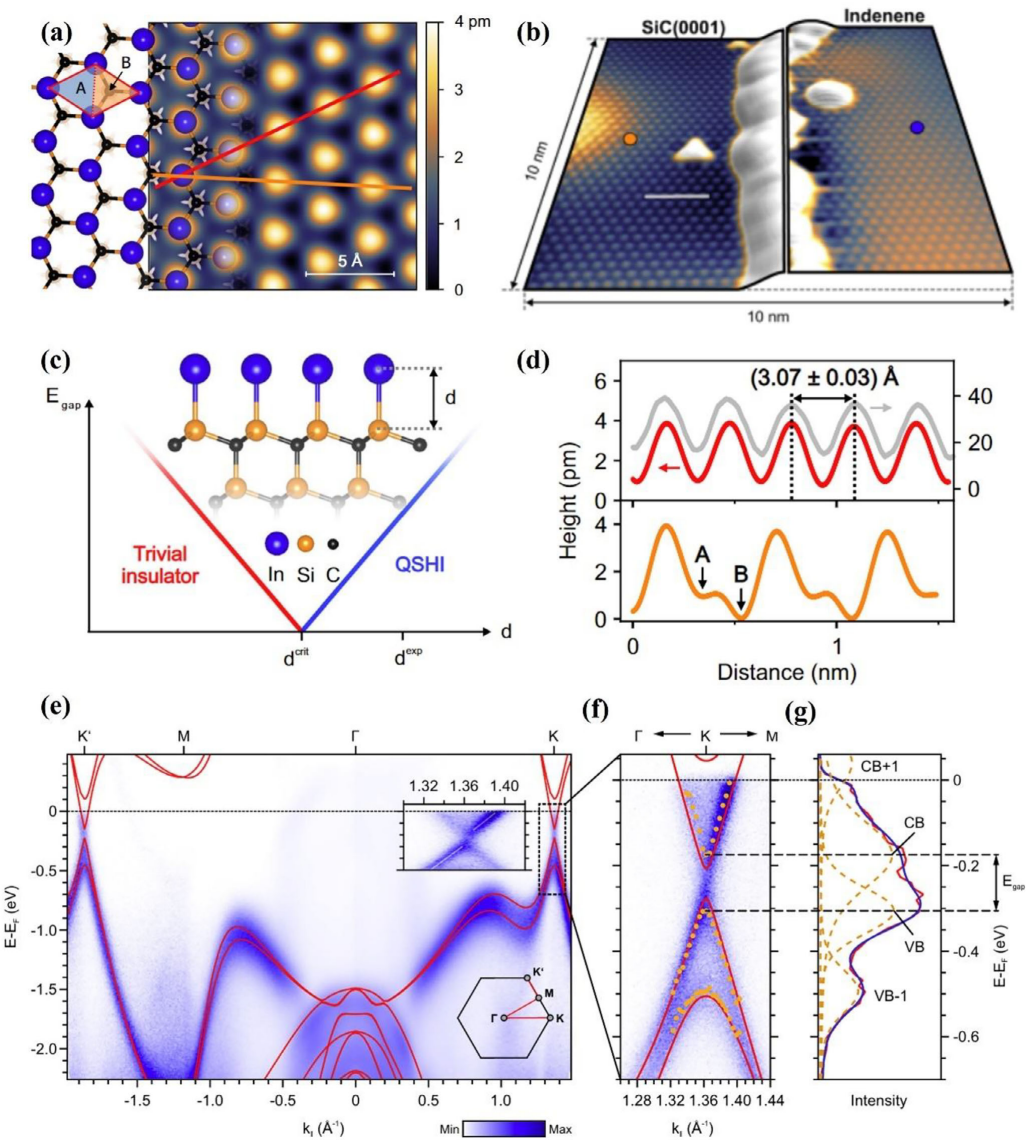
Structural and band characteristics of freestanding indenene. a) STM image showing the triangular lattice of indenene and the (1×1) unit cell with distinct A and B sites; b) The film edge between the freestanding indenene monolayer and the uncovered SiC substrate; c) Side view of the structural model highlighting the In–Si bond at the interface; d) STM height profiles along the red and orange lines in (a), revealing lattice constant and A/B asymmetry; gray curve from bare SiC confirms matching lattice periodicity; e) Angle‐Resolved Photoemission Spectroscopy (ARPES) spectra overlaid with DFT‐calculated bands (red) along high‐symmetry directions. The faint features near the Fermi level are artifacts from He–I satellite lines. Inset: zoomed‐in view at the K‐point showing a clear band gap between upper and lower Dirac cones; f) Energy distribution curve (EDC) peak positions near the K‐point, marked in orange, extracted from fits at selected momentum values; g) EDC at the K‐point (red) with fitted peaks (orange dashed lines) and total fit (blue), resolving VB‐1, VB, and CB states. Reproduced under the terms of the CC‐BY license.^[^ ^57^ ^]^ Copyright 2021, Maximilian Bauernfeind et al.

Indenene is a new member of the 2D material family. Early theoretical research on indenene used the lattice structure of 2D materials with honeycomb lattice.^[^ ^75^ ^]^ Bulk indium possesses a body‐centered tetragonal structure rather than a vdWs layered structure. However, certain atomic planes in the unit cell mimic the geometry of buckled indenene, suggesting that epitaxial techniques may yield monolayer indenene. Bauernfeind et al. considered that In may be arranged in a triangular lattice when deposited on a substrate with a hexagonal structure.^[^ ^57^ ^]^ They successfully realized the triangular lattice structure of monolayer indium on the surface of SiC (0001). The substrate used is 4H‐SiC (0001), prepared by hydrogen atmosphere dry etching process in which hydrogen atoms are introduced to SiC. The substrate that has undergone the above steps has an atomically flat and ordered surface to promote the epitaxial growth of indium. After thermal desorption of surface‐saturated hydrogen from the substrate, ultra‐high purity indium (99.9999%) was used as the evaporation source. The indium atoms preferentially bonded to sites other than the silicon atoms on the substrate, resulting in the successful formation of a monolayer structure, as shown in **Figure** **5**a,b. The triangular lattice of indenene has been confirmed, and the side view of the structure model of indenene (Figure 5c) and the STM height profile (Figure 5d) have been reported, which is conducive to the further exploration of the electronic structure and topological properties of indenene.

##### Properties and Potential Applications of Indenene—Electronic Devices

The exploration of the synthesis of indenene has revealed that the topological properties of indenene can be tuned by adjusting synthesis conditions, which sparked the interests of researchers in the prospect of indenene as a 2D quantum spin Hall (QSH) insulator material. The angle‐resolved photoelectron spectroscopy and DFT calculated energy band structure show that in the presence of SOC, a band gap with a size of E = 70 meV exists. As shown in Figure 5e, this gap occurs between the spin‐split Dirac‐like bands centered at the K/K′ points, which are clearly resolved in both the DFT‐calculated red curves and the experimentally observed ARPES spectra. Notably, the experimental data confirm that the upper and lower Dirac cones do not connect continuously, forming a true energy gap rather than a semi‐metallic touching point. Furthermore, Figure 5f presents a detailed energy distribution curve (EDC) peak fitting near the K‐point, where the orange markers indicate experimentally extracted band positions. These peaks precisely follow the Dirac‐like dispersion and clearly resolve the conduction and valence band edges, enabling accurate determination of the gap size. The presence of bandgap allows further topological features of indenene to be observed.^[^ ^57^ ^]^ According to DFT calculations, it is found that its topology can be changed by the relative strength of inversion‐symmetry breaking (ISB) and SOC. Due to the presence of SiC substrate, the smaller the bond length d of indium and Si as shown in Figure 5c, the stronger the effect of ISB on the indenene layer. The calculated equilibrium bond distance of In/SiC (*d* = 2.68 Å) is very consistent with the one obtained by X‐ray standing wave (*d* = 2.67 ± 0.04 Å). This alignment supports the substrate‐induced inversion symmetry breaking observed in the STM height asymmetry (Figure 5d), which directly lifts the A/B sublattice degeneracy and contributes to the band inversion. Furthermore, the EDC fitting at the K‐point (Figure 5g) reveals three distinct peaks, with a minimum direct band gap of ≈125 meV further validating the SOC‐induced topological band structure. The congruence between the theoretical and experimental bond lengths substantiates the presence of ISB and SOC resulting from Si–In bonding, which in turn leads to the emergence of additional electronic states at the interface, thereby inducing the formation of topological edge states. This confirms the non‐trivial topological nature of indene as a material.^[^ ^57^ ^]^ However, indenene is inherently unstable and can only be studied under UHV conditions, such as STM and X‐ray photoelectron spectroscopy (XPS), limiting the exploration of its properties. A very recent study has proposed a solution to overcome the shortcomings of the inherent instability of indenene. In topological physics, intercalation is usually used to adjust the energy gap induced by SOC of graphene materials.^[^ ^76^ ^]^ Schmitt et al. reversed the roles of the intercalation material and graphene, where graphene is used as the intercalation to protect indenene and make it a stable QSH insulator.^[^ ^77^ ^]^ The In/SiC material with a graphene‐protected thin layer was characterized under non‐UHV conditions and showed excellent performance. Although the conductivity of graphene may interfere with edge transmission measurements, it has undoubtedly succeeded in protecting the indenene‐based QSH insulator material, providing a solution for the manufacture of QSH insulator devices. Indenene exhibits a tunable SOC‐induced band gap and nontrivial topological features confirmed by DFT and ARPES. Graphene intercalation enables stabilization under ambient conditions. However, current studies remain at the fundamental level, with no practical device integration yet achieved. Further efforts are needed for scalable synthesis and edge‐state transport validation.

#### 3.1.4 Thallene

##### Synthesis—Bottom‐Up Method

Bulk thallium crystallizes in a hexagonal structure with metallic bonding, lacking the vdWs layered character required for exfoliation. Therefore, the synthesis of thallene requires a bottom‐up approach. Gruznev et al. followed the synthesis process of other Xenes and fabricated thallene by epitaxial growth in 2020.^[^ ^58^ ^]^ NiSi_2_/Si (111) was selected as the substrate due to its unique compatibility with thallium atoms. This substrate provides a stable template, promoting the crystallization of Tl atoms into a honeycomb lattice. Specifically, the interaction between the Tl atom and the NiSi_2_ layer is crucial for stabilizing the thallium structure. The substrate has the ability to induce the ordered structure of Tl atoms, thereby forming the desired planar honeycomb lattice. DFT calculations also prove that the existence of NiSi_2_ layers is energy‐beneficial for the formation of thallene. As a more recent advance in thallene synthesis, Mihalyuk et al. further improved the quality and functionality of thallene monolayers by introducing an interfacial Sn layer between thallium and the NiSi_2_/Si(111) substrate.^[^ ^59^ ^]^ The Sn decoration effectively decouples thallene electronically from the substrate, enabling the formation of large‐scale, flat honeycomb thallene with minimal substrate‐induced perturbation. This interface engineering strategy not only stabilizes thallene growth but also introduces promising spintronic functionalities, making it a compelling candidate for future 2D‐Xene–based devices.

##### Properties and Potential Applications of Thallene—Electronic Devices

**Figure 6:**
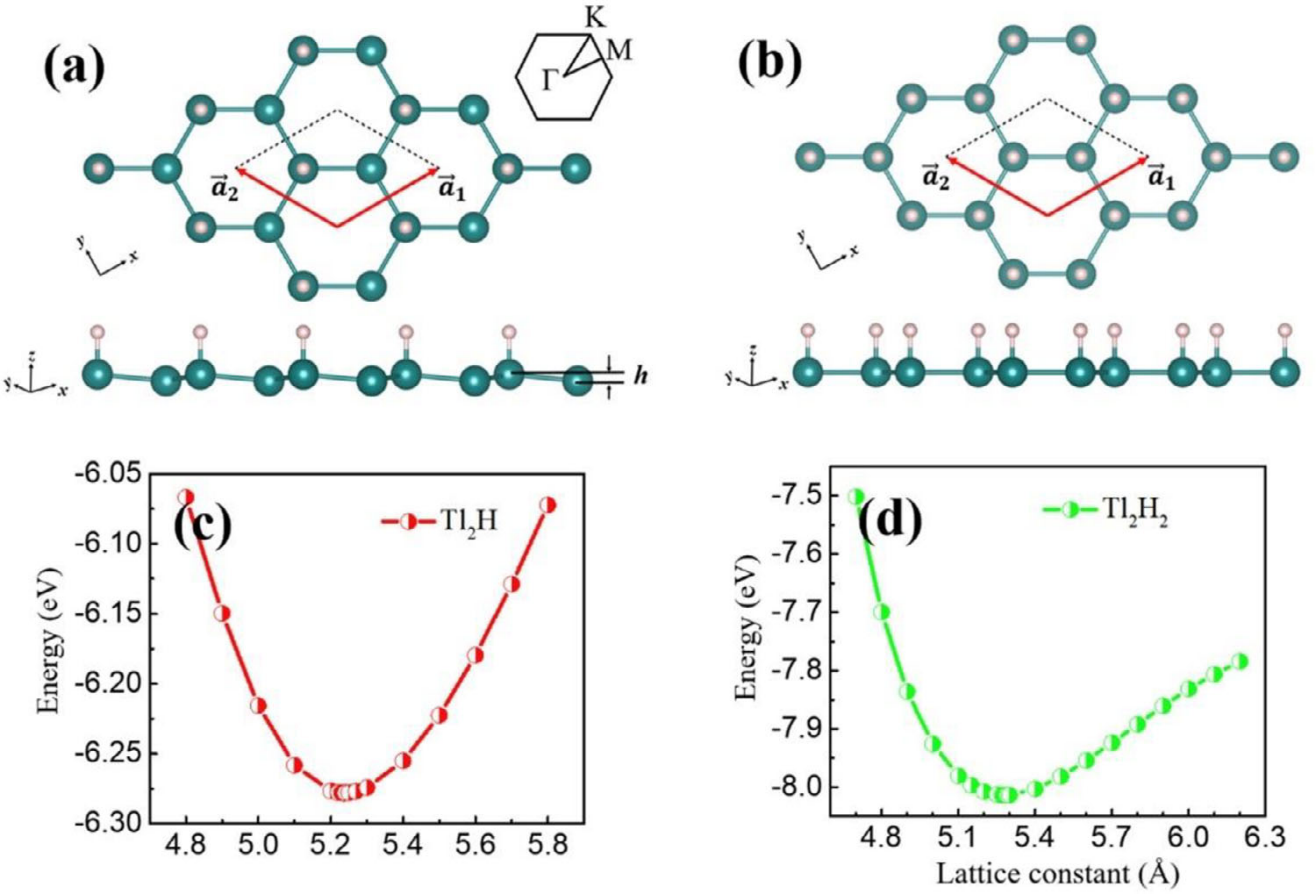
Atomic structures and energetic profiles of Thallene. The top and side views of the structure of a) Monolayer Tl_2_H and b) Monolayer Tl_2_H_2_. The green balls represent Tl, and the white balls represent H atoms, respectively; c,d) The total energy of monolayer Tl_2_H and Tl_2_H_2_ structures varies with the lattice constants. Reproduced under the terms of the CC‐BY license.^[^ ^78^ ^]^ Copyright 2023, Xiaojuan Liu et al.

There are few reports on the properties and potential applications of thallene. However, a recent study explored the properties of hydrogenated thallene. Since the QSH effect of graphene was reported,^[^ ^14^ ^]^ many graphene‐like 2D materials have also been found to possess this effect. As a topological trivial extension semiconductor material, the QSH effect of thallene can be realized by large biaxial strain.^[^ ^78^ ^]^ Gruznev et al. reported that the structure of monolayer thallene is a flat 2D honeycomb lattice structure without buckling.^[^ ^58^ ^]^ Building on this work, Liu et al. established the molecular model of hydrogenated thallene (Tl_2_H and Tl_2_H_2_) and performed DFT calculations.^[^ ^78^ ^]^ Due to the bonding of different hydrogen atoms, the lateral geometry of these two forms of thallene shows low curvature (**Figure** **6**a) and plane (Figure 6b) structures. The optimal lattice constants of Tl_2_H and Tl_2_H_2_ are 5.24 Å and 5.28 Å, respectively, as shown in Figure 6c,d. The calculated formation energies of the two types of thallene hydrides are ‐1.60 eV and ‐2.23 eV, respectively, which indicates that the reaction between thallene and hydrogen molecules is an exothermic reaction, and the feasibility of functional synthesis of this material is proved. The QSH states of the two thallene remain stable at 5% strain and have topological nontrivial band gaps much higher than those of most QSH insulators. The foundation for the creation of topological electrical devices operating at ambient temperature is believed to come from thallene materials. DFT calculations confirm the feasible synthesis and structural stability of thallene. Compared to other QSH materials, thallene offers promising band topology without buckling. However, current findings remain theoretical, and experimental realization and device integration have yet to be achieved.

### 3.2 Group IVA

Belonging to group IVA, carbon, silicon, germanium, tin, and lead share a valence configuration of ns^2^np^2^, enabling the formation of four covalent bonds.^[^ ^31^, ^32^ ^]^ Although carbon can easily form a planar sp^2^ hybridized graphene lattice, other common IVA elements can also exist in a 2D hexagonal structure under restricted conditions, although they usually exhibit slight buckling to stabilize their 2D configuration. **Table** **2** summarizes the synthesis and morphology of the four Xenes (silicene, germanene, stanene, plumbene) in group IVA.

**Table 2:** Xenes of Group IVA elements.

Xenes	Precursor	Synthetic method	Morphological characteristics	Refs.
Silicene	Si	MBE on Ag (111)	Hexagonal honeycomb structure, with a height of approximately 0.1 nm	[79]
	Silicon film	PLD in Argon atmosphere	Hexagonal lattice, novel rectangular lattice structure (lattice distance 0.23 nm)	[80]
	CaSi_2_	Wet‐chemical exfoliation	An accordion‐like microstructure, each layer with a thickness of ≈0.6 nm	[81]
	CaSi_2_	Vacuum–Nitrogen Assisted topotactical deintercalation	Multilayer stacked flakes, lateral size from 2–3 µm to ≈100 µm	[82]
Germanene	Ge	MBE on Au (111)	Clear hexagonal honeycomb structure, smooth surface	[83]
	Ge	MBE on Pt (111)	Hexagonal honeycomb structure with folded configuration (0.6 Å)	[84]
	GeCa_2_	Mechanical exfoliation	Smooth surface, uniform thickness, and layered structure	[85]
Stanene	Sn	MBE on Bi_2_Te_3_ (111)	Buckling hexagonal honeycomb lattice with wrinkles (approximately 0.1 nm) with a random height modulation (0.06 nm)	[86]
	Li_5_Sn_2_	LPE	Height of 4 nm and transverse dimension of several micrometers, lattice spacing of 0.29 nm	[27]
Plumbene	Pb	MBE on Pd (111)	Hexagonal lattice structure, unit cell size is ≈0.48 nm, flat structure	[87]

#### 3.2.1 Silicene

##### Synthesis—Bottom‐Up Method

**Figure 7:**
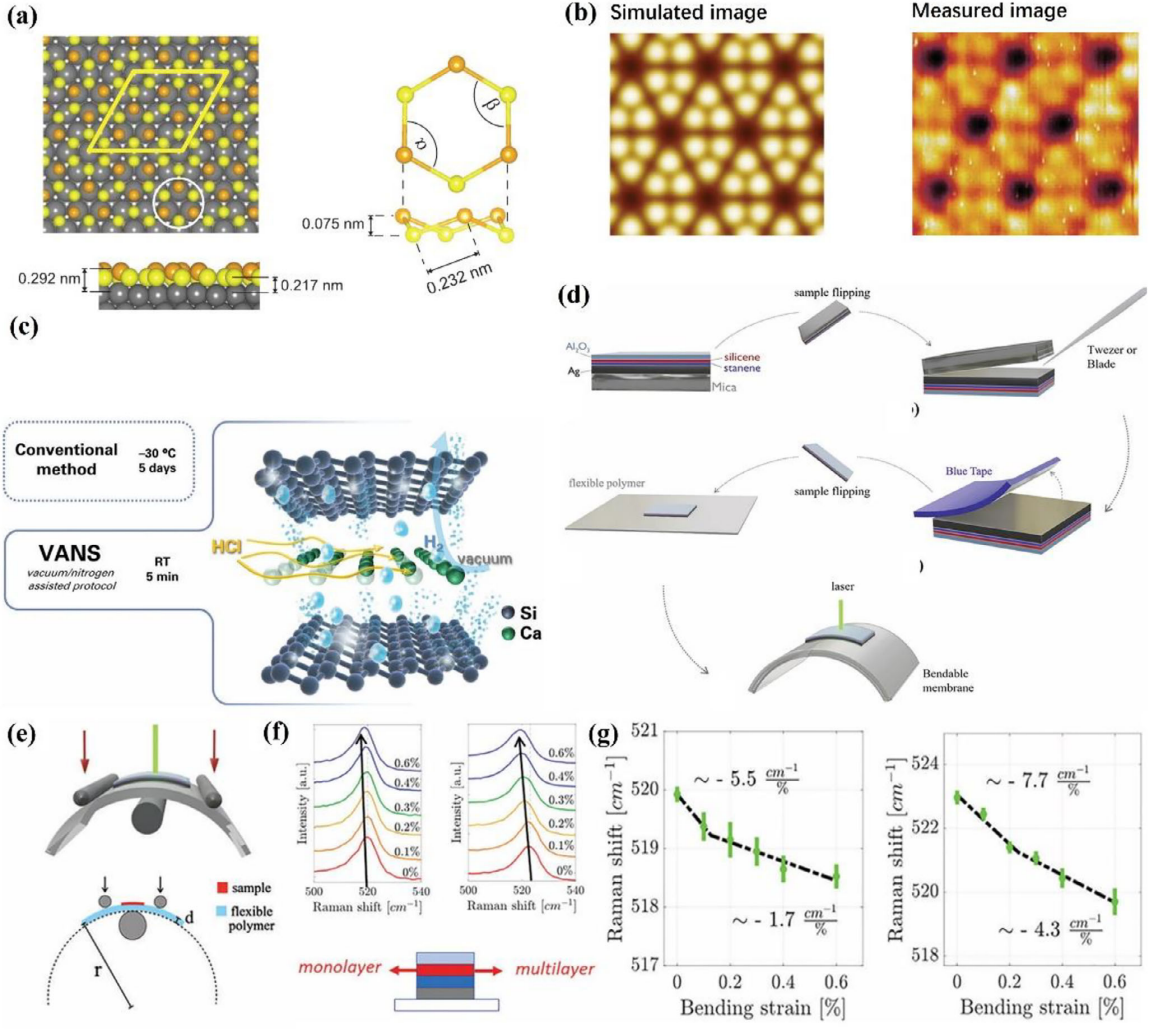
From atomic structure to device integration: synthesis and strain engineering of silicene‐based materials. a) Atomic structure of silicene on Ag(111): top view, side view, and highlighted hexagonal unit cell (white circle); b) Left: Simulated silicene structure STM image; Right: Experiment observed STM image for silicene structure. Reproduced with permission.^[^ ^79^ ^]^ Copyright 2012, American Physical Society. c) Schematic illustration of CaSi_2_ deintercalation into silicene via conventional and VANS methods. Reproduced under the terms of the CC‐BY license.^[^ ^82^ ^]^ Copyright 2024, Erika Kozma et al. d) Schematic illustration of the fabrication process for silicene‐based bendable membranes, including epitaxial growth, encapsulation, mechanical delamination, and transfer onto flexible substrates; e) Schematic of the three‐point bending setup used to apply uniaxial strain to bendable silicene membranes; f) Raman peak positions of monolayer and multilayer silicene membranes under applied strain; g) Raman shift of monolayer (left) and multilayer (right) silicene–stanene heterostructures as a function of applied strain. Reproduced under the terms of the CC‐BY license.^[^ ^92^ ^]^ Copyright 2023, Christian Martella et al.

As a Group IVA element like carbon, silicon has been considered for the possibility of having a 2D structure for decades. As early as 1994, Takeda and Shiraishi proposed a theoretically stable wavy aromatic configuration in which silicon can form.^[^ ^33^ ^]^ In 2007, Guzman‐Verri et al. further investigated this theory and named the 2D nanostructure of silicon as silicene.^[^ ^31^ ^]^ However, silicon contains sp^3^ hybridization of strong covalent bonds, and there is no allotrope of silicon in nature, as graphite for graphene. It is not easy to fabricate silicene by mechanical exfoliation methods, like the way of graphene synthesis.^[^ ^12^ ^]^ Therefore, the epitaxial growth method was used in experiments to synthesize silicene. In 2010, synthetic silicon nanoribbons were reported.^[^ ^2^ ^]^ Nonetheless, the fabrication approach of silicene based on MBE was reported in 2012 Ag (111) was used as a substrate, which was cleaned by Ar^+^ sputtering and annealed at 530 °C. Then, silicene was fabricated by depositing directly heated silicon crystal as the precursor on the substrate surface between 220 and 260 °C under UHV.^[^ ^79^ ^]^ The DFT simulation results calculated by Vogt et al. are shown in **Figure** **7**a, including both the side and top views of the silicene structure. Figure 7b compares the simulated silicene structure with the experimental STM image, and the two structures are consistent.^[^ ^79^ ^]^ The silicene film presents a honeycomb structure, and the thickness is approximately 0.1 nm obtained by measuring the edge of the film.

Unlike graphene, the honeycomb lattice structure of silicene exhibits a curved arrangement due to its sp^3^/sp^2^‐like hybridization, which is also consistent with earlier theoretical studies.^[^ ^31^, ^33^ ^]^ The success of the experiment of epitaxial growth to synthesize silicene has aroused greater interest, and subsequent substrates such as Ir (111),^[^ ^88^ ^]^ MoS_2_,^[^ ^89^ ^]^ and Pb (111)^[^ ^90^ ^]^ have also been reported. Interestingly, silicon atoms grow on the same substrate to form different structures, such as herringbone structure and graphene‐like honeycomb crystal structure on the surface of Ru (001). This difference can be attributed to the coverage of silicon atoms. The herringbone structure is more favorable in terms of energy, resulting in low atomic coverage. As the atomic coverage increases, new silicon atoms attach to the elbow of the existing herringbone structure to form a hexagon, which gradually develops into a honeycomb structure.^[^ ^91^ ^]^ A novel method of synthesizing silicene by pulsed laser deposition (PLD) method has been proposed in recent years. The report uses a high‐energy pulsed laser beam (wavelength 1064 nm) directed at the surface of a silicon wafer to heat the silicon to a molten or vaporized state rapidly.^[^ ^80^ ^]^ The molten or vaporized silicon was then transported in the form of plasma to the surface of an amorphous carbon substrate, which was eventually deposited and formed as a film of amorphous silicon. Then, according to the nanoscale characteristics of electron de Broglie waves, silicene crystals were generated by electron beam irradiation (with a density of 0.5 nA nm^−2^). Through TEM characterization, silicene crystals have two structures: one is a hexagonal lattice, and the other is a newly discovered rectangular lattice (with a lattice distance of 0.23 nm). Theoretical calculations show that rectangular lattice silicene exhibits lower energy than hexagonal lattice during the optimization process and shows better dynamic stability.^[^ ^80^ ^]^

##### Synthesis—Top‐Down Method

However, the difficulty of silicene transfer from the substrate due to epitaxial growth limits further research. A top‐down approach was reported recently. This report suggested a method to synthesis silicene by wet‐chemical exfoliation.^[^ ^81^ ^]^ Bulk silicon adopts a diamond cubic lattice with strong covalent sp^3^ bonding, thus lacking the weak interlayer forces required for exfoliation. Consequently, silicene synthesis in this work relies on topochemical deintercalation from layered Zintl‐phase CaSi_2_ rather than direct exfoliation from elemental silicon. The silicon layer is diffused from the outside to the inside by the oxidation of I_2_ to obtain multilayer silicon nanosheets. Through thermal expansion, nitrogen cooling, and gasification, monolayer silicene can be exfoliated. The obtained multilayer silicene nanosheets have a structure similar to that of an accordion and the thickness of each layer is 0.6 nm. Similarly, another method for large‐scale preparation of silicene based on liquid oxidation and CaSi_2_ exfoliation has also been proposed.^[^ ^93^ ^]^ Building upon these top‐down strategies, Kozma et al. introduced a Vacuum–Nitrogen Assisted (VANS) deintercalation method, which enables the rapid production of multilayer hydrogen‐terminated silicene nanosheets under ambient conditions as shown in Figure 7c.^[^ ^82^ ^]^ This method alternates vacuum and nitrogen purging to accelerate the removal of hydrogen gas generated during the reaction with hydrochloric acid, thereby significantly enhancing the deintercalation kinetics. Compared to previous methods that require days of low‐temperature processing, the VANS method completes the synthesis within minutes and yields high‐quality silicene with minimal oxygen contamination and reduced defect density. Such an approach offers improved scalability and compatibility with flexible or solution‐processed electronics, positioning VANS‐derived silicene as a promising candidate for future device‐level integration.

##### Properties and Potential Applications of Silicene—Energy Storage

The research on the application of silicon‐based materials in the field of energy storage, particularly in LiBs, has a long history. However due to the significant volume expansion and contraction of silicon during the charge‐discharge cycle, the development of this field is challenging.^[^ ^94^ ^]^ Similar to Group IIIA borophene, silicene has been explored for energy storage because of its excellent ability to bind lithium atoms and its ultra‐high theoretical capacity.^[^ ^95^ ^]^ The low diffusion barrier (0.2 eV) of silicene is also a competitive advantage for its applications in LiBs.^[^ ^96^ ^]^ Binary system studies of silicon and lithium propose that each silicon atom can hold 4.4 lithium atoms, reaching a specific insertion capacity of 4200 mAh g^−1^.^[^ ^17^ ^]^ The 2D layered structure of silicene can also buffer the volume change. Liu et al. carried out DFT calculations of silicenes they synthesized to explore the maximum Li adsorption numbers (0.5 and 0.43, respectively) of monolayer and bilayer silicenes, and the capacities were calculated as 954 and 715 mAh g^−1^, respectively. The electrochemical performance of LiBs using silicene as anode material was also evaluated by cyclic voltammetry. After 100 cycles, silicene still retains 76% of its original reversible capacity, demonstrating cycling stability. Additionally, silicene has excellent electrochemical performance even at an extremely high current density (5 A g^−1^) and maintained stable capacity (312 and 596 mAh g^−1^, respectively) after 1800 cycles.^[^ ^93^ ^]^ Another report also obtained the same theoretical capacity in LiBs of single and bilayer silicene anodes and calculated the volume change of their physicochemical processes to 13% and 24%, respectively.^[^ ^97^ ^]^ Silicene has also been found to have potential applications in supercapacitors. Quantum capacitance refers to the amount of charge stored per unit mass of a material and is an important parameter for evaluating the performance of supercapacitors. An ab initio molecular dynamics study based on non‐equilibrium Green's function combined with DFT was used to calculate the quantum capacitance of graphene, silicene and carbon nanomaterials. The results showed that, in terms of weight capacitance, silicene double‐layer capacitors had the highest specific capacitance (electrostatic potential energy (EP) of 2.59 µF cm^−2^), followed by graphene (EP of 2.35 µF cm^−2^) and carbon nanotubes (EP of 1.36 µF cm^−2^).^[^ ^98^ ^]^ Guo et al. for the first time assembled silicene into a high‐voltage symmetric supercapacitor with a voltage window of 0–3 V, a maximum specific capacitance of 0.41 mF cm^−2^, an energy density of up to 1.22 mJ cm^−2^, and a capacitor retention rate of up to 96.6% after 10 000 cycles, demonstrating the excellent electrochemical performance of silicene for supercapacitor applications.^[^ ^99^ ^]^ Silicene offers high theoretical capacity and low Li diffusion barriers, supporting its potential in LiBs and supercapacitors. Its 2D structure mitigates volume expansion, while both theoretical and experimental results show stable cycling and excellent rate performance. Notably, silicene exhibits superior quantum capacitance compared to graphene. Despite these advantages, practical deployment is still hindered by synthetic and structural stability challenges.

##### Electronic Devices

Silicene has been predicted to have the QSH effect in previous studies.^[^ ^100^ ^]^ As a QSH‐based quantum mechanical switch, a topological insulator field‐effect transistor can be controlled via topological phase transitions, enabling on and off switching based on the presence or absence of topological edge states.^[^ ^13^ ^]^ These states, including non‐trivial and trivial ballistic QSH modes, can determine the topological phase of the material. Compared with traditional solid‐state dielectrics (breakdown field is 0.7 eV nm^−1^), the critical field required for silicene is only 0.05 eV nm^−1^ to undergo a topological phase transition.^[^ ^6^ ^]^ In addition, materials with stronger SOC will lead to a higher operating temperature, making the critical electric field relatively high. The intermediate SOC possessed by silicene (1.55 meV)^[^ ^100^ ^]^ makes it a better candidate for topological insulator field‐effect transistors. To realize topological insulator field‐effect transistors (TI‐FETs) based on silicene, both scalable membrane fabrication and strain‐responsiveness are essential. Martella et al. reported a complete transfer process to obtain bendable silicene and silicene–stanene heterostructure membranes from Ag(111)/mica substrates by using mechanical delamination and flexible substrate attachment as shown in Figure 7d.^[^ ^92^ ^]^ These membranes were subjected to uniaxial strain using a custom‐built three‐point bending apparatus (Figure 7e), allowing in situ Raman measurements under controlled deformation. The Raman spectra of multilayer silicene–stanene heterostructures displayed a clear redshift with increasing strain (Figure 7f), with a peak shift rate reaching −7.7 cm^−1^ per percentage (Figure 7g). Silicene's QSH effect and low critical field enable topological phase switching, offering promise for TI‐FETs. Its moderate SOC supports potential room‐temperature operation. Recent advances in flexible membrane fabrication and strain‐controlled Raman tuning demonstrate its structural responsiveness. Nonetheless, scalable synthesis and robust edge‐state retention remain key challenges for device integration.

##### Biomedical Fields

Silicene and its derivatives have also shown interesting performance in the biomedicine field. Lin et al. proposed the application of the silene‐based materials they synthesized in oncology medicine.^[^ ^81^ ^]^ In their study, the degradation performance of silicene was investigated and compared with other inorganic nano agents, and it was found that silicene has unique absorption properties and excellent photothermal conversion efficiency in the near‐infrared (NIR) region, making it a strong candidate for photothermal cancer therapy. Silicene nanosheets modified by bovine serum albumin are biocompatible and can induce apoptosis of breast cancer cells in mouse experiments under near‐infrared laser irradiation. Another study obtained silicene @ Pt composite nanosheets by in‐situ generation and growth. The surface was modified with organic soy phospholipids (SP) to improve biocompatibility. The synthesized silicene @ Pt‐SP exhibits near‐infrared light absorption in the ultraviolet visible spectrum.^[^ ^101^ ^]^ In order to further explore its photothermal conversion performance, an 808 nm laser with different power densities was irradiated, and the photothermal conversion efficiency of silicene @ Pt‐SP (30.1%) was calculated to be higher than that of Cu_9_S_5_ (25.7%) and bismuth sulfide nanorods (28.1%).^[^ ^101^ ^]^ Silicene and its composites exhibit strong NIR absorption and high photothermal conversion efficiency, showing promise for cancer therapy. Modified with proteins or phospholipids, they demonstrate good biocompatibility and tumor ablation effects in vivo. Notably, silicene @ Pt‐SP achieves 30.1% conversion efficiency, surpassing other nanomaterials. However, practical biomedical translation requires further assessment of safety, degradation, and delivery.

#### 3.2.2 Germanene

##### Synthesis—Bottom‐Up Method

**Figure 8:**
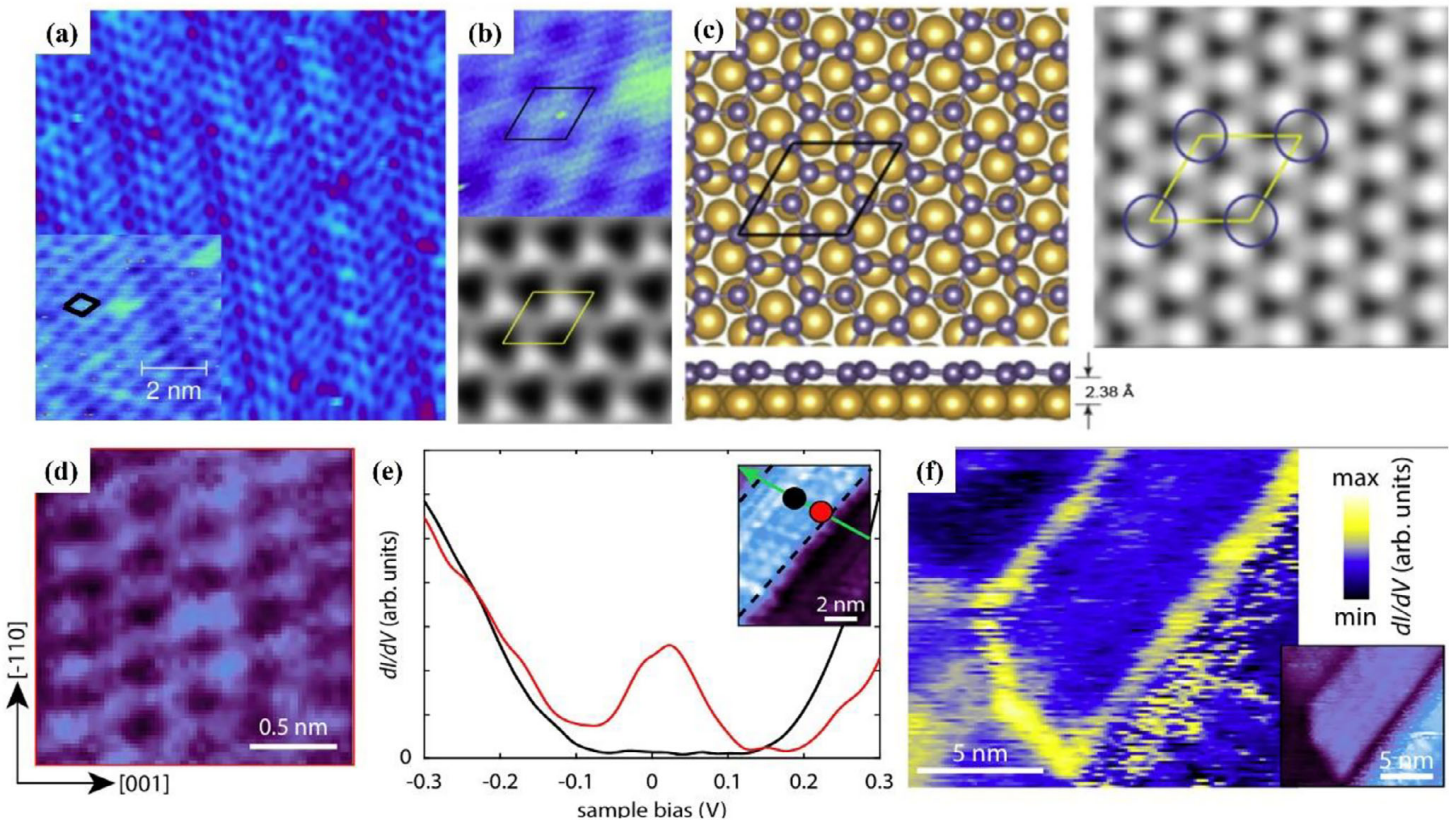
Atomic imaging and electronic structure of Germanene for topological applications. a) STM image of germanene honeycomb superstructure, the inset is part of close‐up STM image, black represents the unit cell of germanene; b) Comparison of experimental STM (top) and simulated STM (bottom) images of germanene; c) Left: Top and side view of germanene structure on Au (111) surface; Right: Simulated STM image of germanene on Au (111) surface. Reproduced under the terms of the Creative Commons Attribution licence.^[^ ^83^ ^]^ Copyright 2014, IOP Publishing Ltd. d) STM image revealing the honeycomb lattice of germanene with zigzag edges; e) *dI*/*dV* spectra showing edge‐localized conductance peaks within the topological gap; f) Spatial map of the edge state, confirming continuous conduction along the ribbon boundary. Reproduced under the terms of the CC‐BY‐NC‐ND.^[^ ^105^ ^]^ Copyright 2025, Dennis J. Klaassen et al.

Germanene was predicted to have the same buckled honeycomb lattice structure as silicene in 2009.^[^ ^32^ ^]^ Laboratory synthesis of germanene was initially attempted using the MBE method on the surface of Ag (111). However, due to the strong interaction between germanene and the Ag (111) surface, complex interactions occur between Ge atoms and Ag surface atoms, which may even form surface alloys or induce significant lattice distortions.^[^ ^83^ ^]^ Dávila et al. selected Au (111) substrate, which was also a noble metal, and successfully obtained germanene by the MBE method. The method used in this experiment is similar to the steps they reported for the synthesis of silicene,^[^ ^79^ ^]^ and a similar growth process resulted in a √3 × √3 reconstructed germanene layer on a √7 × √7 Au (111) supercell.^[^ ^83^ ^]^ The STM image of germanene obtained at a growth temperature of 200 °C is shown in **Figure** **8**a. Germanene exhibits a clear and nearly flat honeycomb structure. The simulated STM image of germane is shown in Figure 8b, and the simulation results confirm the reliability of the molecular structure of the synthesized germanene.^[^ ^83^ ^]^ The researchers also reported the atomic structure of germanene on the surface of Au (111), as shown in Figure 8c. In the same year, another research group successfully synthesized germanene on a Pt (111) surface using the MBE method.^[^ ^84^ ^]^ It is worth noting that the annealing process in the range of 600–750 K was used here to prevent the formation of Ge‐Pt surface alloy during synthesis. Through low‐energy electron diffraction and STM observations, germanene has a hexagonal honeycomb structure and an undulation of ≈0.6 Å. Combined with the DFT calculation results, the formation of germanene nanomaterials was confirmed. More substrates, for example, Al, hexagonal aluminum nitride (AlN), Cu, and beyond have been used for epitaxial growth synthesis of germanene, expanding the possibility for this controlled synthesis and integration into functional devices.^[^ ^102^, ^103^, ^104^ ^]^

##### Synthesis—Top‐Down Method

Although large pieces of germanium crystallize in a diamond cubic structure and cannot be exfoliated directly, the Zintl‐phase CaGe_2_ has layered and wrinkled Ge^−^ sheets, which are separated by Ca^2^⁺ ions. This makes top‐down methods possible. One work utilized this structural feature of Zintl‐phase CaGe_2_. First, high‐purity Ge and Ca were reacted in a vacuum through high‐temperature reactions to prepare CaGe_2_. A chemical displacement reaction was then carried out between hydrochloric acid and CaGe_2_ to replace calcium atoms with hydrogen atoms, generating germanane (hydrogenated germanene). Then, through the mechanical exfoliation method, germanane was exfoliated from the bulk crystal into thin sheets. Finally, these thin sheets were subjected to thermal annealing treatment in an argon atmosphere to remove hydrogen atoms and thereby transform into germanene.^[^ ^85^ ^]^ The thickness of the germane sheets after annealing was uniformly reduced by approximately 40%. This is consistent with the interlayer spacing of germanene (≈3.2 Å) and that of germanane (5.5 Å). Meanwhile, the thickness of the entire thin sheet varies uniformly, and the surface of the thin sheet is smooth, which further supports the annealing process and retains the 2D structure and preeminent crystallinity of the germanene sheet.

##### Properties and Potential Applications of Germanene—Energy Storage

The 2D properties of germanene make it as promising as silicene in the field of energy storage.^[^ ^95^ ^]^ Germanene has been shown to have a hexagonal structure^[^ ^79^ ^]^ and strongly layered properties (with a repeat period of 3.2 Å) that make it have a large specific surface area. The property of a large surface area allows a high concentration of Na^+^ to be inserted into germanene.^[^ ^106^ ^]^ Liu et al. also measured that germane nanosheets had an initial discharge capacity of 695 mAh g^−1^ at a current density of 0.1 A g^−1^, outperforming other germanium‐based materials such as GeH (490 mAh g^−1^) and Ge particles (296 mAh g^−1^). In terms of initial Coulomb efficiency, germanene also showed superior performance at 60.8%, compared to 48.8% for GeH and 49.6% for Ge particles. At the same time, the 2D ultra‐thin nanostructure of germanene can effectively promote the transport of electrons and ions, shorten the diffusion path of Na^+^, and accelerate the absorption of Na^+^.^[^ ^107^ ^]^ Germanene shows promising potential for sodium‐ion storage due to its high surface area and Na⁺ mobility, with initial electrochemical validation; however, practical integration remains limited by stability, synthesis scalability, and long‐term performance assessments.

##### Properties and Potential Applications of Germanene—Electronic Devices

Germanene nanoribbons offer a unique platform for next‐generation topological quantum devices owing to their intrinsic SOC and robust edge states. Their potential is rooted in their atomically precise structure and symmetry, as revealed by high‐resolution scanning tunneling microscopy.^[^ ^105^ ^]^ Figure 8d presents an atomic‐resolution STM image showing the low‐buckled honeycomb lattice of a germanene nanoribbon with zigzag edge termination, a structural motif known to support topologically protected edge modes. Functionally, these edge states manifest as prominent conductance features in local tunneling spectra. As demonstrated in Figure 8e, a clear peak in the differential conductance (*dI*/*dV*) spectrum appears at ≈30 meV when the STM tip probes the nanoribbon edge, confirming the existence of a localized state within the bulk bandgap. And its bulk region remains in an insulated state, which is exactly as expected based on the characteristics of topological insulators. Furthermore, the spatial distribution of these states as visualized in Figure 8f, reveals a continuous signal running along the nanoribbon edges, indicative of 1D topological channels with dissipationless transport characteristics. Such properties position germanene nanoribbons as highly attractive candidates for ultra‐compact quantum circuits, where arrays of parallel edge channels can be harnessed to build high‐density quantum interconnects. It is worth noting that when the bandwidth is reduced to approximately 2.6 nm or less, the system undergoes a dimensional transformation: one‐dimensional edge states disappear, and zero‐dimensional end states with symmetry protection are generated.^[^ ^105^ ^]^ Conceptually, this is similar to the Majorana zero mode. Germanene nanoribbons feature strong SOC and support tunable topological states, enabling dissipationless edge transport and potential use in quantum devices.

Recent studies reveal that twisted bilayer germanene can host electrically tunable quantum valley Hall states, thanks to its sizable spin–orbit coupling and intrinsic bandgap.^[^ ^108^ ^]^ When the applied electric field exceeds a critical threshold, bandgap inversion occurs in AB/BA domains, giving rise to a triangular network of topologically protected 1D channels. This controllable valley‐protected transport lays the groundwork for a novel type of robust and low‐dissipation quantum valley Hall transistor. Germanene integrates tunable topological states into both edge‐confined and moiré‐modulated transport scenarios, offering versatile platforms for quantum circuitry. Its strong spin‐orbit coupling property enables the formation of stable and lossless channels, and it also has the ability to be controlled by an electric field. This is more advantageous than graphene without a band gap. Nonetheless, ambient instability, nanoscale twist control, and integration challenges constrain practical deployment. However, experimental verifications such as atomic‐level resolution edge patterns and field‐induced band inversion have further confirmed the technical feasibility of this technology. These findings collectively indicate that germanene is expected to be an ideal material for manufacturing compact, low‐power, and topologically protected quantum devices.

##### Properties and Potential Applications of Germanene—Biomedical Fields

Germanene also has good biocompatibility and light absorption capacity in the NIR region. A hydrogel functionalized composite based on germanene was used by Feng et al. to explore its potential applications in drug delivery.^[^ ^109^ ^]^ Doxorubicin was loaded in germanene‐hydrogel to test the drug release behavior. Upon NIR radiation, the drug was successfully released triggered by the localized temperature rise resulting from the strong NIR absorbance of germanene, and antibacterial effects were also observed.

#### 3.2.3 Stanene

##### Synthesis—Bottom‐Up Method

**Figure 9:**
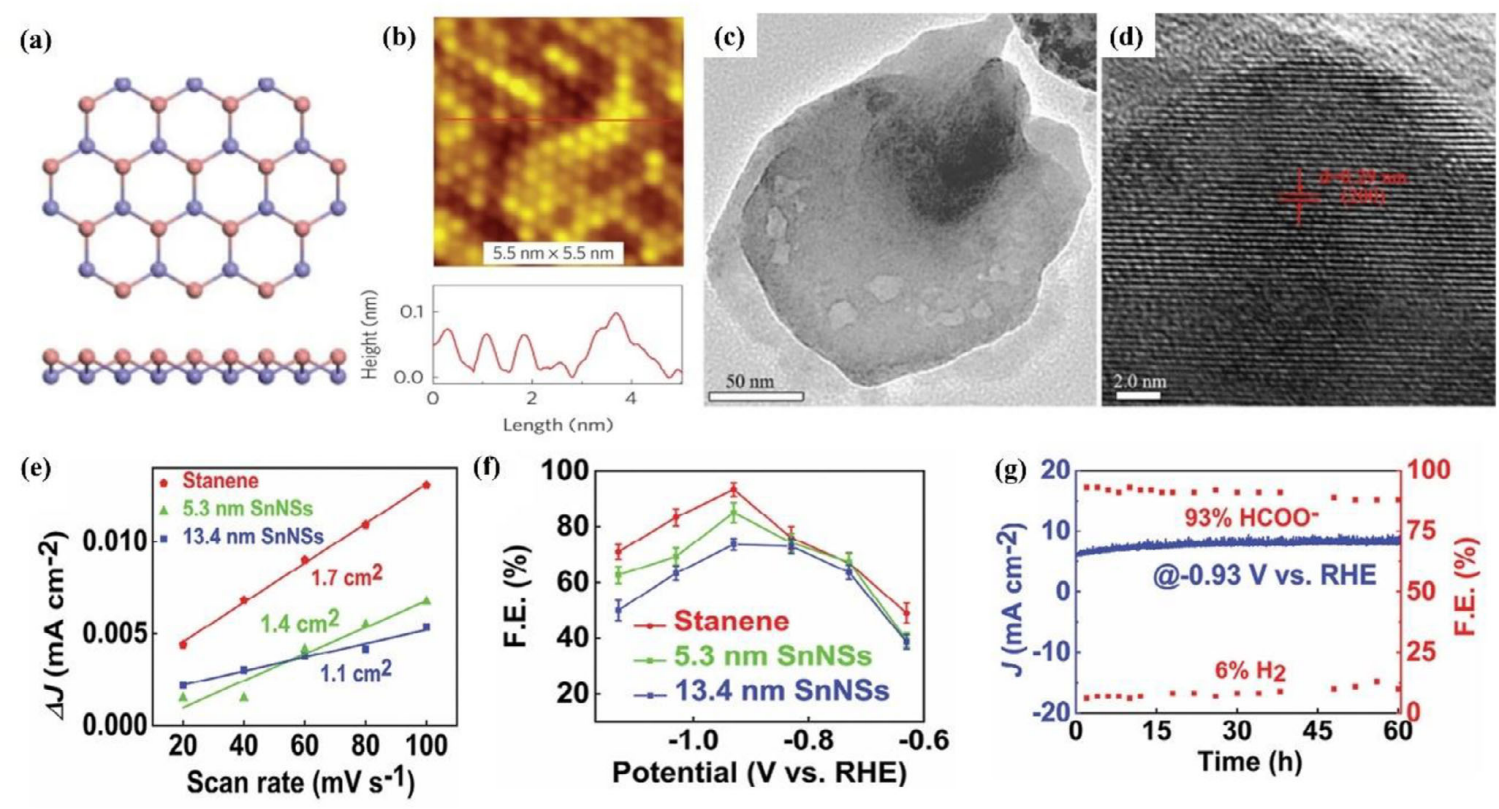
Structural characterization and electrocatalytic CO_2_ reduction performance of Stanene. a) Lattice structure of stanene. Top view (upper) and side view (lower) where red shows the top Sn atoms and blue shows the bottom Sn atoms; b) STM image of stanine (top) and height line profile (bottom); Reproduced with permission.^[^ ^86^ ^]^ Copyright 2015, Springer Nature. c) TEM image of stanene; d) HR‐TEM image of stanene. Reproduced with permission.^[^ ^27^ ^]^ Copyright 2019, Royal Society of Chemistry publication. e) Charging current density differences (Δ*J*) versus scan rates for Sn nanosheets of varying thicknesses, used to calculate their electrochemical surface areas (ECSA); f) Faradaic efficiency (F.E.) for formate production as a function of applied potential for Sn nanosheets with different thicknesses; g) Long‐term CO_2_RR at −0.93 V reveals stable F.E. for HCOO^−^ and H_2_ on stanine; Reproduced with permission.^[^ ^114^ ^]^ Copyright 2025, John Wiley and Sons.

The first experimental synthesis of stanene, another Xenes in group IVA, was achieved using the MBE method. In this experiment, MBE method successfully grew stanene films on Bi_2_Te_3_ (111) substrates. High‐purity tin (99.999%) was deposited onto the Bi_2_Te_3_ (111) substrate at a rate of ≈0.4 monolayers per minute at room temperature.^[^ ^86^ ^]^ The top and side views of the stanene lattice are shown in **Figure** **9**a. Its surface matches the step and platform structure of the substrate and has a hexagonal honeycomb lattice structure as shown in Figure 9b. The hexagonal honeycomb structure of Stanene is bulking, and the height difference between the top and bottom Sn atoms is usually about 0.1 nm. Due to the compressive stress within the substrate and stanene film planes, as well as the presence of hydrogen adsorption during the growth process, stanene has a random height modulation of approximately 0.06nm. More substrates, such as InSb (111),^[^ ^110^ ^]^ Ag (111),^[^ ^111^ ^]^ Cu (111),^[^ ^112^ ^]^ and PbTe (111),^[^ ^113^ ^]^ were successfully used to grow stanene in further studies, providing a research basis for its further research and fabrication.

##### Synthesis—Top‐Down Method

Sn crystallizes in a body‐centered tetragonal structure at room temperature, forming a three‐dimensional metallic network that lacks any vdWs layered character. This prevents Sn from being directly exfoliated into 2D materials via top‐down methods. A method combining LPE with dealloying successfully synthesized stanine.^[^ ^27^ ^]^ Ma et al. utilized a layered Li_5_Sn_2_ alloy synthesized by melting lithium and β‐Sn at 600 °C under argon. The alloy contains biatomic Sn layers separated by lithium, which can be selectively removed in aqueous solution, enabling the release of few‐layer stanene nanosheets via ultrasonic stripping. Figure 9c,d shows the transmission electron microscope (TEM) image and high‐resolution TEM (HR‐TEM) image of stanene obtained in the experiment.^[^ ^27^ ^]^ The thickness of stanene is ≈4 nm, and its transverse dimensions are at the micrometer level. Stanene nanosheets have a lattice width of ≈0.29 nm, corresponding to the (200) lattice plane of the bulk tin.

##### Properties and Potential Applications of Stanene—Energy Storage

Although the theoretical capacitance of stanene (226 mAh g^−1^) is not as high as other group IVA Xenes like silicene, Mortazavi et al. conducted DFT calculations and found that the activation energy for Li diffusion in stanene is only 0.1 eV,^[^ ^95^ ^]^ while that in silicene is 0.2 eV.^[^ ^96^ ^]^ They also found that the existence of hollow sites in the hexagonal lattice of stanene makes the adsorption of Li stable and almost constant. Another study on the application of stanene in sodium‐ion batteries highlighted the significance of the hollow sites within stanene. The findings demonstrate that defects associated with these hollow sites can notably lower the activation energy of stanene after hydrogen passivation, thereby enhancing its Na^+^ storage capacity. This defective stanene can have a capacity of up to 272 mAh g^−1^.^[^ ^115^ ^]^ Due to the limitations in the experimental synthesis of stanene, most related research to date has relied on the DFT calculations. However, the controlled synthesis of defective stanene has emerged as a promising direction for future energy storage applications.

##### Properties and Potential Applications of Stanene—Topological Insulators

Topological insulator materials have attracted much attention because of their unique electronic properties. In particular, the spin alignment of conductive electrons on the surface has attracted widespread attention in material science and condensed matter physics. The feasibility of low‐energy operation of non‐dissipative conductive edge states on the surface has also garnered considerable interest in these fields.^[^ ^116^ ^]^ Earlier studies have shown that heavier elements are thought to have a stronger SOC effect.^[^ ^100^, ^117^ ^]^ The large mass of Sn atoms makes it represent a stronger SOC effect than materials such as graphene in group IVA. Hence, stanene is considered a good topological insulator. Theoretical studies based on first‐principal calculations have announced that the band gap of pure stanene is 0.1 eV, and after chemical functionalization (e.g., ─F, ─Cl, ─Br, ─I, and ─OH), the bandgap can be increased to 0.3 eV.^[^ ^117^ ^]^ By changing the chemical functional groups, the topological properties of materials can be changed. Such modification can regulate the Fermi velocity of the edge state, and even the topological phase of the material can be qualitatively changed. External strains (such as compression or tension) can also regulate the band inversion and QSH state, which makes it possible to regulate QSH insulators experimentally. Through functionalization and strain engineering, stanene's topological characteristics can be precisely modulated, enabling bandgap opening and control over QSH states. While these strategies highlight its potential for room‐temperature applications, translating such tunability into robust, scalable devices remains an open challenge.

##### Properties and Potential Applications of Stanene—Catalytic Field

Stanene has recently emerged as a promising electrocatalyst for the carbon dioxide reduction reaction (CO_2_RR), particularly in the selective formation of formate (HCOO^−^). Unlike conventional Sn‐based catalysts such as nanoparticles, nanowires, or bulk Sn nanosheets, stanene exhibits distinct advantages due to its atomic‐scale thickness, high density of edge sites, and favorable electronic structure. In a recent study, free‐standing stanene was synthesized for the first time via a scalable wet‐chemical route, yielding monolayer nanosheets with an average thickness of ≈0.7 nm and a porous morphology that enhances the exposure of catalytically active Sn(100) step edges.^[^ ^114^ ^]^ The superior electrochemical surface area (ECSA) of stanene, compared to thicker Sn nanosheets, further confirms the efficient exposure of active sites (Figure 9e). Electrochemical tests revealed a high Faradaic efficiency of up to 93% at −0.93 V vs. reversible hydrogen electrode as shown in Figure 9f, while in situ Mössbauer spectroscopy confirmed that the presence of zero‐valent Sn is closely correlated with formate selectivity. DFT calculations further identified the Sn(100) step edge as the primary active site, where the CO_2_‐to‐formate pathway proceeds with low energy barriers and suppressed competing HER activity. Moreover, the long‐term CO_2_RR performance test at −0.93 V showed that stanene maintains a stable current density and Faradaic efficiency over extended operation, highlighting its durability under practical conditions (Figure 9g).^[^ ^114^ ^]^ This study elucidates the mechanistic origins of stanene's high CO_2_RR selectivity, highlights its structural and functional advantages over traditional Sn‐based catalysts, and offers a technically feasible framework for scalable CO_2_ conversion, including detailed assessment of its working principles, performance metrics, and practical limitations.

#### 3.2.4 Plumbene

##### Synthesis—Bottom‐Up Method

**Figure 10:**
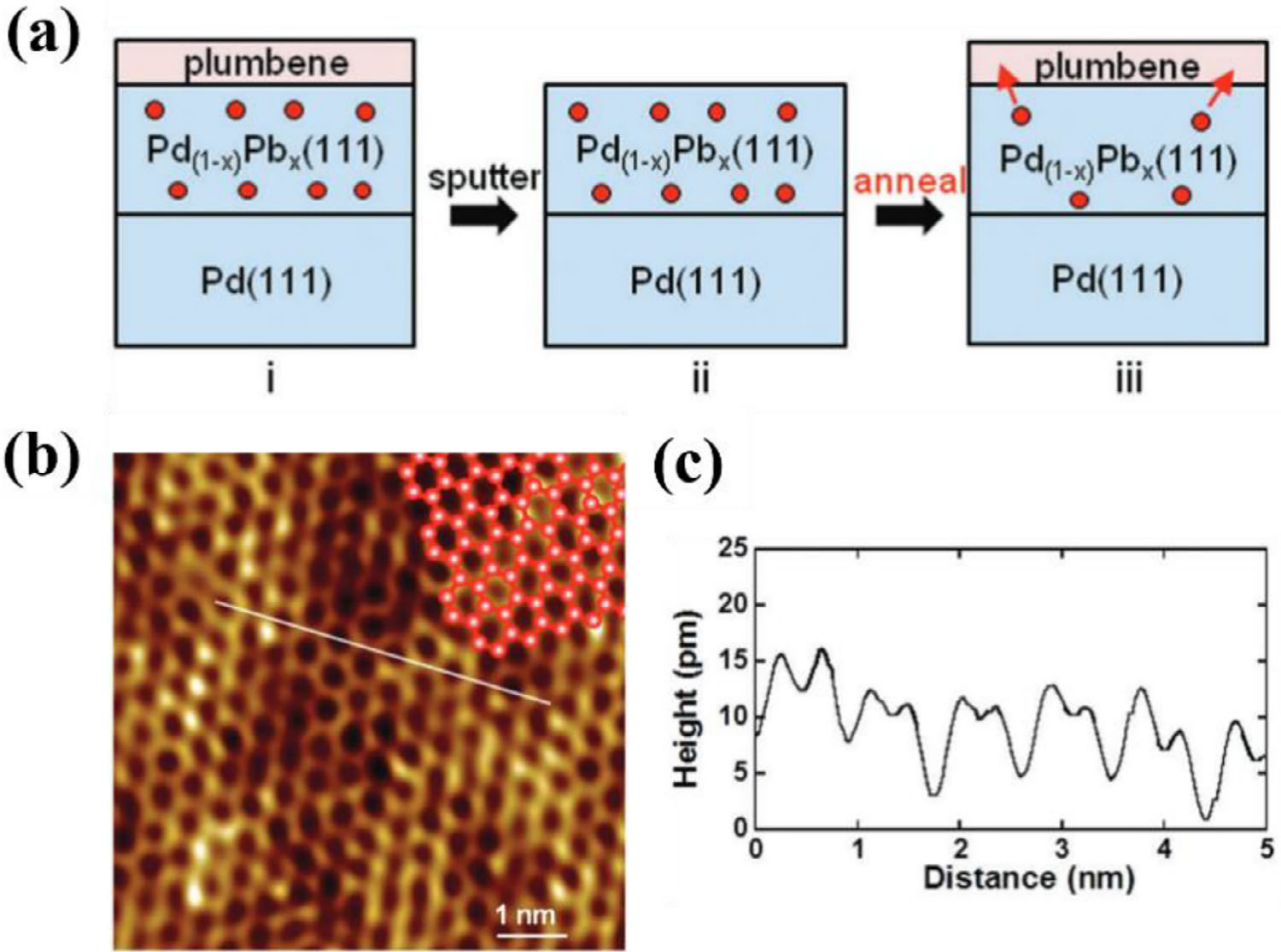
The experimental synthesis and characterization of plumbene. a) Schematic diagram of plumbene synthesis process where i) Plumbene on the Pd_1−x_Pb_x_ (111) alloy film on Pd (111), ii) Remove plumbene after sputtering and iii) Pb segregation of plumbene growth after annealing; b) STM atomic structure image, red balls are the highlight of the honeycomb structure; c) Height profile of white line in (b). Reproduced with permission.^[^ ^87^ ^]^ Copyright 2019, John Wiley and Sons.

Plumbene is the most recently synthesized Xenes among the group IVA elements. Bulk lead crystallizes in a face‐centered cubic structure with metallic bonding, forming a three‐dimensional isotropic lattice. This non‐layered structure lacks vdWs gaps, thereby precluding direct exfoliation into 2D plumbene via top‐down strategies. Yuhara et al. successfully fabricated plumbene using MBE, following a deposition protocol similar to those used for other graphene‐like materials.^[^ ^87^ ^]^ The experiment selected Pd (111) as the substrate, and the substrate was cleaned with 2 keV Ar^+^ sputtering and annealed at 850 °C. Then, under UHV and room temperature conditions, Pb was deposited on the substrate at a rate of 0.4 mL min^−1^. When Pd (111) was annealed at 600 °C, a solid solution of plumbene and Pd_1‐x_Pb_x_ (111) was formed on the surface. After further sputtering with Ar^+^ ions and annealing of the solid solution, the Auger electron spectroscopy signal shows the formation of plumbene through segregation (the process diagram is shown in **Figure** **10**a). The flat honeycomb structure of plumbene was obtained by atomic STM images, as shown in Figure 10b, along with the height profile (Figure 10c).^[^ ^87^ ^]^ The unit cell size is approximately 0.48 nm. A more recent study used Ir (111) as the substrate to grow a flat honeycomb structure of plumbene on a laid Fe monolayer.^[^ ^118^ ^]^

##### Properties and Potential Applications of Plumbene—Hydrogen Storage

Plumbene is considered to have potential as a hydrogen storage material because of its large specific surface area and similar nanostructure to that of 2D materials such as graphene.^[^ ^119^ ^]^ Vivek et al. calculated the interaction between plumbene and hydrogen molecules based on DFT. Hydrogen gravity density of plumbene up to 6.74 wt% is obtained from the calculations, which has reached the standard of becoming the next generation hydrogen storage material recommended by the US Department of Energy (5.5wt%).^[^ ^119^ ^]^ At the same time, they calculated the average adsorption energy (*E*_avg_) of plumbene for hydrogen. The computed result is −117 meV, which is higher than that of graphene (−63.181 meV) and hexagonal boron nitride (−60.352 meV), indicating a stronger interaction between hydrogen molecules and the plumbene surface. Furthermore, the influence of an external electric field on hydrogen adsorption performance was investigated. The results showed that the *E*_avg_ was within the required energy range under a positive electric field, suggesting that plumbene enables controlled hydrogen uptake and release. With strong H_2_ adsorption energy and a DOE‐compliant storage capacity, plumbene offers a structurally tunable platform for reversible hydrogen storage. External electric fields further enable dynamic control, bridging theoretical feasibility with future functional deployment.

### 3.3 Group VA

In group VA, 2D materials of phosphorus, arsenic, antimony, and bismuth have all been successfully prepared. Interestingly, during the exploration of 2D materials, two allotropes of phosphorus (blue phosphorus and black phosphorus) were found to have two 2D structures. The synthesis method and morphology of the VA group Xenes are shown in **Table** **3**.

**Table 3:** Xenes of Group VA elements.

Xenes	Precursor	Synthetic method	Morphological characteristics	Refs.
Phosphorene (black)	Black phosphorus	Mechanical exfoliation	Single‐layer and few‐layer 2D black phosphorus, transverse dimensions ranges from several micrometers to tens of micrometers	[3]
	Black phosphorus	LPE	Single‐layer black phosphorus nanosheets with good crystallinity	[126]
	Black phosphorus	PLD	Thickness ranges from 2 nm to 10 nm, transverse dimension is approximately 4.9 nm	[127]
Phosphorene (blue)	Black phosphorus	MBE on Au (111)	Arranged in a wrinkled hexagonal structure with a height of approximately 2.3 Å	[128]
	Black phosphorus	MBE on Au (111)	Height of 0.15 nm and lattice constant of 0.35 nm	[129]
Arsenene	As	MBE on Ag (111)	A wrinkled honeycomb structure, a lattice constant of 3.6 Å	[130]
	As	LPE with NMP solvent	Excellent crystallinity and layered structure, with a thickness of 5–12nm	[131]
Antimonene	Sb	MBE on Ge (111)	Thickness is 2–7 layers (single layer thickness of about 5.24 Å), obvious wrinkles	[132]
	Sb	Mechanical exfoliation	Monolayer antimonene is 0.4nm, a hexagonal lattice structure and good crystallinity	[133]
	β ‐phase Sb	Pressurized Alloying Assisted Synthesis	Lateral dimension is 3 µm, thickness is less than 2 nm, wrinkled hexagonal lattice structure	[134]
Bismuthene	Bi	Epitaxial growth on SiC (0001)	Hexagonal honeycomb structure, lattice constant is 5.35 Å	[4]
	Bi	LPE with sonochemical	Honeycomb structure, lattice constant is 0.322 nm, thickness is 4 nm	[135]
	Bi	Ionic liquid‐assisted grinding and exfoliation method	Rhombohedral lattice structure, transverse size of 20 nm, thickness of 2 nm	[136]

#### 3.3.1 Phosphorene

##### Synthesis

Several allotropes of phosphorus (white phosphorus, black phosphorus, and blue phosphorus)^[^ ^120^, ^121^ ^]^ have been reported so far. This review will discuss the synthesis of 2D black phosphorus (BKP) and 2D blue phosphorus (BLP) using both top‐down and bottom‐up synthesis methods.

##### 2D Black Phosphorus

**Figure 11:**
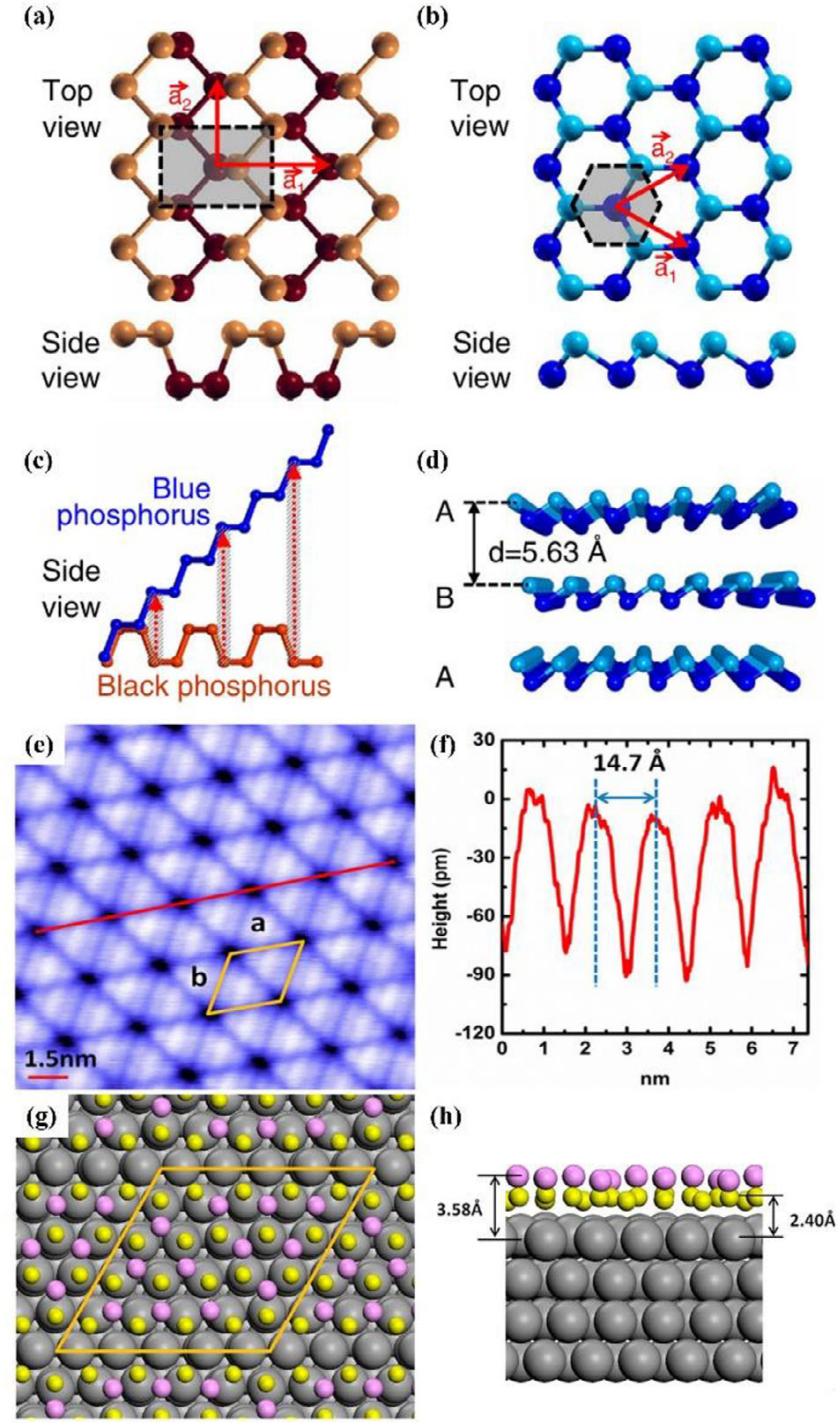
Atomic structure of BKP and BLP. Top and side view of the monolayer honeycomb structure of a) black and b) blue phosphorus. Different shades of color are used to represent the top and bottom positions of the atoms; c) Schematic diagram of converting black phosphorus to blue phosphorus by dislocation method; d) Side view of the structure of A‐B stacked blue phosphorus. Reproduced with permission.^[^ ^121^ ^]^ Copyright 2014, American Physical Society. e) HR‐STM image of single layer blue phosphorus on Au (111). The highlighted orange rhombus represents the unit cell of the phosphorus where *a* = *b* = 14.7 Å; f) The profile along the red line in (e), the distance between black void is 14.7 Å; g) Top and h) side view of the simulated DFT images for single layer blue phosphorene on the Au (111) surface. The top phosphorus atoms are represented as purple and the bottom as yellow balls, respectively. Au atoms are represented as gray balls. Reproduced with permission.^[^ ^128^ ^]^ 2016 American Chemical Society.

BKP is an allotrope of phosphorus discovered in 1914,^[^ ^120^ ^]^ and has been shown to be thermodynamically more stable than other phosphorous allotropes.^[^ ^122^ ^]^ The layered structure formed through the linked atom of BKP has drawn more attention after the exfoliation of graphene.^[^ ^123^ ^]^ The weak vdWs force interaction between the layered structures of BKP makes it possible to obtain 2D black phosphorus, commonly referred to phosphorene, by mechanical exfoliation.^[^ ^3^ ^]^ As shown in **Figure** **11**a, both the top and side view of the honeycomb crystal structure of black phosphorus calculated by DFT is stacked with adjacent layers relying on the interaction of weak vdWs forces.^[^ ^121^ ^]^ By improving the ‘Scotch tape method’, C‐Gomez et al. peeled off high‐purity BKP (99.998%) with blue Nitto tape several times. Then, the tape with BKP microcrystals was pressed onto the polydimethylsiloxane substrate. BKP sheets of varying thickness can be distinguished under an OM. Through Raman spectroscopy and SEM image characterization, it was confirmed that monolayer and multilayer BKP were obtained, and their transverse dimensions could range from several micrometers to tens of micrometers.^[^ ^3^ ^]^ However, the mechanical exfoliation method is still limited to laboratory scale,^[^ ^124^ ^]^ and the exfoliated BKP nanosheets are irreversibly oxidized when exposed to ambient environmental conditions, which limits their long‐term stability and practical applications.^[^ ^125^ ^]^ An LPE method using NMP as an organic solvent has been successfully used to exfoliate bulk BKP to get BKP nanosheets.^[^ ^126^ ^]^ Following ultrasonication at 30 W for one hour, the dispersion was centrifuged to obtain fully exfoliated 2D BKP nanosheets. The BKP nanosheets obtained by LPE have a single‐layer number and good crystallinity and possess performance comparable to that of mechanically exfoliated BKP nanosheets. However, to meet the requirements of phosphorene for practical device applications, smaller and more uniform 2D BKP nanosheets are required. As a result, the bottom‐up synthesis approaches have attracted more interest as a promising alternative. A method for synthesizing ultrathin BKP films based on PLD method was proposed by Yang et al.^[^ ^127^ ^]^ The work uses a range of substrate materials (graphene, Cu, or SiO_2_/Si substrate) and BKP as the target under vacuum conditions. Using KrF pulsed laser ablation with a repetition rate of 5 Hz, the film was grown at a substrate temperature of 150 °C. In order to obtain a uniform film, both the substrate and the target are rotated in this experiment. The thickness range of 2D BKP is from 2 nm to 10 nm, and the transverse dimension is approximately 4.9 nm.^[^ ^127^ ^]^

##### 2D Blue Phosphorus

Another allotrope of phosphorus, BLP, is also considered to have the potential to become a graphene‐like 2D material. As shown in Figure 11b, based on DFT calculations, stable BLP is generated by the flipping part of phosphorus atoms without changing the bond angle (as shown in Figure 11c). Figure 11d shows the layered structure of BLP, indicating the possibility of single‐layer BLP synthesis.^[^ ^121^ ^]^ However, to the best of the author's knowledge, there have been no reports on top‐down exfoliation methods for BLP so far. The synthesis of 2D BLP is achieved by adopting the bottom‐up method. Zhang et al. proposed a technique for growing monolayer blue phosphorus on Au (111) substrate using the MBE method.^[^ ^128^ ^]^ This work used clean Au (111) after repeated bombardment with Ar^+^ as substrate and BKP as a phosphorus source. After depositing at 260 °C and annealing at 250 °C for an hour, monolayer blue phosphorene was synthesized. The HR‐STM image of this phosphorene as shown in Figure 11e. Interestingly, the ordered structure of this phosphorene presents a wrinkled hexagonal shape, which is consistent with the silicene grown on Ag (111).^[^ ^79^ ^]^ This can be attributed to the similar lattice parameters and sawtooth lattice edges of silicene and phosphorene, which is of great significance for the design and synthesis of other 2D materials. The yellow diamond shows the unit cell structure of the blue phosphorene, and Figure 11f shows the height profile along the red line in Figure 11e, showing a distance of 14.7 Å between the centers of adjacent phosphorus vacancies (dark holes). The calculation results based on DFT are shown in Figure 11g,h. The orange diamond in Figure 11g is the supercell with a side length of 14.49 Å, while Figure 11h shows the height between the top and bottom phosphorus atoms, with the Au being 3.58 and 2.4 Å, respectively.^[^ ^128^ ^]^ This result was further confirmed by another MBE synthesis method, which also uses Au (111) as substrate. In that work, the researcher also obtained blue phosphorene arranged in a buckled honeycomb lattice but with the InP as a phosphorus source.^[^ ^129^ ^]^ The height of the grown 2D BLP is approximately 0.15 nm, with the lattice constant of 0.35 nm. More recent studies have extended substrate choices to use Cu,^[^ ^137^ ^]^ Ag,^[^ ^138^ ^]^ or Pt^[^ ^139^ ^]^ and successfully synthesized 2D blue phosphorene.

##### Properties and Potential Applications of Phosphorene—Field‐Effect Transistors

**Figure 12:**
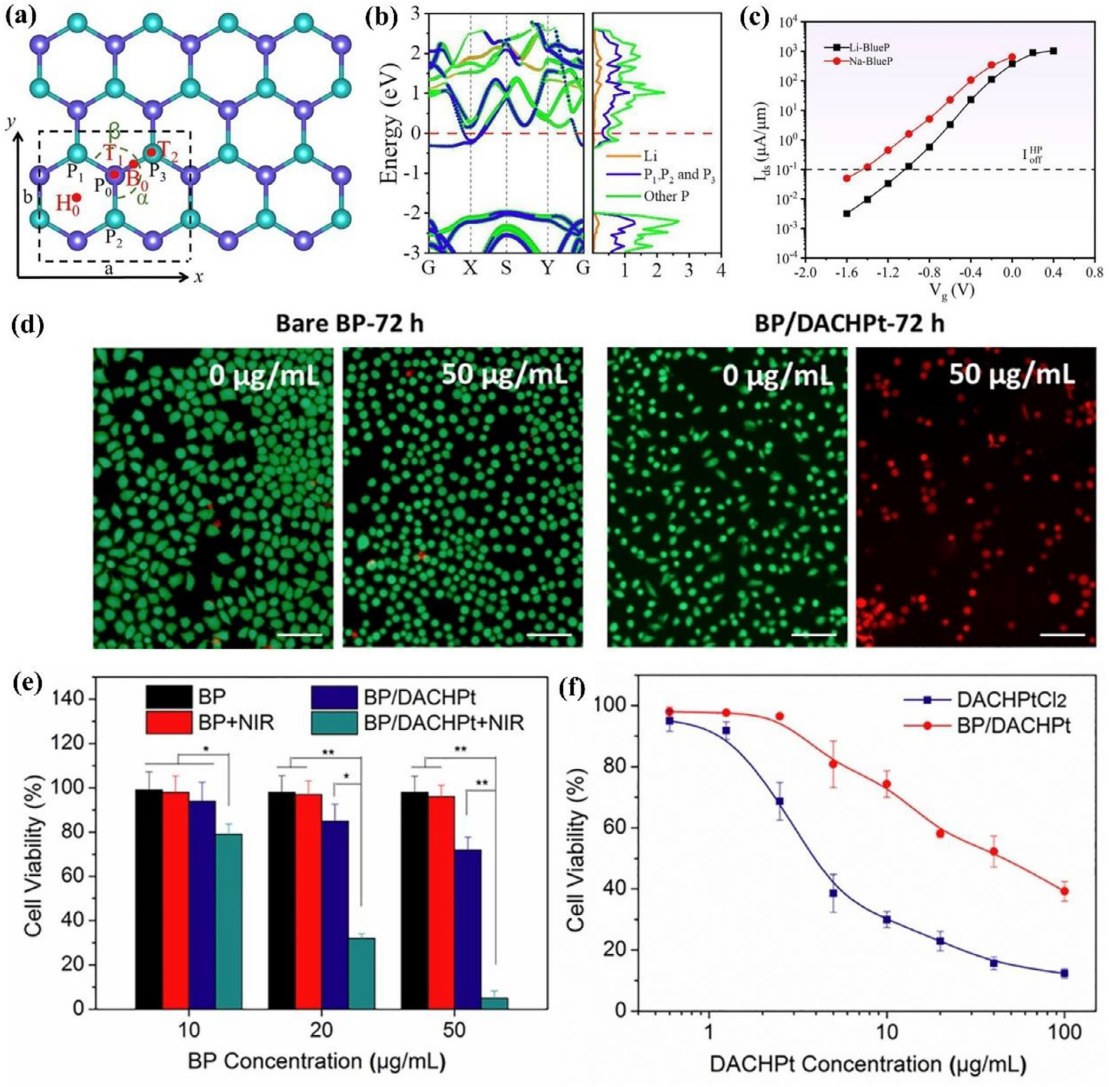
Multifunctional applications of phosphorene in electronics and biomedicine. a) Top view of adsorption sites (H_0_, T_1_, T_2_, B_0_) for adatom X on BLP; b) The energy band structure and density of states (DOS) of lithium; c) The *I*_ds_–*V*_g_ curves of two FETs; $I_{OFF}^{HP}$ indicates the off‐state current of the high‐performance international technology roadmap for semiconductors standard; Reproduced with permission.^[^ ^141^ ^]^ Copyright 2025, American Physical Society. d) Fluorescence images of HeLa cells stained with Calcein Acetoxymethyl Ester (green, live) and Propidium Iodide (red, dead) after NIR irradiation, following treatment with bare BKP or BKP/DACHPt ((1R,2R‐diaminocyclohexane)platinum(II)); e) Cell viability after incubation with bare BKP or BKP/DACHPt, with or without NIR irradiation; f) Cell viability after treatment with DACHPtCl_2_ or BKP/DACHPt for 48 h; Reproduced with permission.^[^ ^142^ ^]^ Copyright 2019, Elsevier.

The variable bandgap and high carrier mobility of phosphene make it a promising material for the fabrication of FETs. The tunable band gap ranging from ≈0.3 eV to ≈1.5 eV of phosphorene enables a high on/off current ratio, which is important for efficient switching behavior in FETs.^[^ ^3^, ^10^, ^125^, ^126^ ^]^ In addition, the high carrier mobility of phosphorene (≈1000 cm^2^ V^−1^ s^−1^) makes the rapid charge transport possible, allowing rapid device response.^[^ ^140^ ^]^ These characteristics, including high‐current and high‐power output, make phosphorene advantageous for the applications of transistors. The phosphorene‐based FETs reduce the drain current by a factor of 10^5^ by changing the opening and closing of the channel under a vacuum environment. The modulation of the drain current is ≈10^4^ times larger than that of graphene.^[^ ^140^ ^]^ Building upon these promising features, recent studies have explored interface engineering strategies to further reduce contact resistance and enhance charge injection in phosphorene FETs. One effective approach is the surface adsorption of light elements onto monolayer BLP.^[^ ^141^ ^]^ As shown in **Figure** **12**a, Li atoms preferentially adsorb onto T_1_ sites of BLP, forming stable configurations with minimal lattice deformation. Importantly, Figure 12b illustrates that Li adsorption significantly alters the electronic band structure, shifting the Fermi level into the conduction band and thereby inducing metallic behavior, which was an essential condition for forming Ohmic contacts. Utilizing this effect, BLP‐based FETs with Li‐adsorbed electrodes exhibit superior transport characteristics, as shown in Figure 12c. Compared with Na‐adsorbed electrodes, Li‐BLP contacts enable a higher on‐state current (Ion = 9.0 µA µm^−1^) and a reduced subthreshold swing (SS ≈ 266 mV dec^−1^), indicating stronger gate modulation and improved carrier injection efficiency.^[^ ^141^ ^]^ These results confirm that Li surface adsorption not only achieves low contact resistance but also offers a reliable, defect‐free alternative to conventional doping or metal‐contact engineering, providing a new pathway to optimize phosphorene‐based transistor performance at the atomic scale.

##### Properties and Potential Applications of Phosphorene—Energy Storage

The tunable band gap, high carrier mobility, and great mechanical strength of phosphene also make it a promising candidate for energy storage.^[^ ^10^, ^126^ ^]^ Unlike graphene, the in‐plane bonding sp^3^ hybrid of phosphorene exhibits a buckled structure that makes it useful for supercapacitors.^[^ ^3^ ^]^ When compared with graphene, the larger layer distance enables phosphorene to have good electrical conductivity and rapid ion diffusivity in the applications of supercapacitors. One study reported by Hao et al. synthesized black phosphorene in acetone and successfully fabricated supercapacitors by using LPE to avoid the oxidation of phosphorene in the environment.^[^ ^143^ ^]^ The maximum power density of this supercapacitor can reach 8.83 W cm^−3^, which is ≈10 times higher than similar devices produced with graphene.^[^ ^144^ ^]^ Another work produced a supercapacitor using tightly stacked graphene and phosphorene nanosheets as electrodes. The supercapacitor was tested to have an excellent energy density of 11.6 mWh cm^−3^ and excellent mechanical properties.^[^ ^145^ ^]^ At the same time, phosphorene also had the advantage of a low diffusion barrier (0.04 eV), which was conducive to the application of phosphene in batteries. Phosphene also had a lower diffusion barrier than graphene (≈0.3 eV),^[^ ^8^ ^]^ which made it easier for ions to diffuse in the electrode. Based on DFT calculations with D‐3 corrections to account for vdWs interactions, the specific capacity of Li_0.5_P and Na_0.5_P were calculated to reach 389.02 and 315.52 mAh g^−1^, respectively.^[^ ^146^ ^]^ Combining a low ion diffusion barrier with high power and energy densities, phosphorene bridges structural advantages and electrochemical performance. Its buckled geometry and vdWs‐tuned capacity enable diverse storage applications, from supercapacitors to alkali‐ion batteries.

##### Properties and Potential Applications of Phosphorene—Biomedical Fields

Due to its almost negligible cytotoxicity and excellent biodegradability, phosphorene is considered to be an outstanding emerging material in the field of biomedicine.^[^ ^147^ ^]^ However, its easy oxidation and degradation properties make phosphorene often requires functionalization to ensure stability for specific applications.^[^ ^125^ ^]^ Its strong laser absorption capabilities have attracted researchers in the field of photothermal therapy. Moreover, the tunable band gap of phosphorene allows it to achieve a steerable electromagnetic field interaction in the ultraviolet and near‐infrared regions, making it highly suitable for optoelectronic and photothermal biomedical applications.^[^ ^127^ ^]^ Qin et al. encapsulated black phosphorene and gemcitabine drug in hydrosol and used them on mice carrying 4T1 xenograft tumors. The experimental results showed that the phosphorene has good photothermal efficiency.^[^ ^148^ ^]^ Liu et al. studied the cytotoxicity and apoptotic mechanism of black phosphorene exfoliated by LPE in vitro.^[^ ^142^ ^]^ The phosphorene was loaded with platinum‐based drugs to form a coordination complex. The results demonstrated that the cancer cells were all killed, confirming the strong potential of phosphorene as drug delivery platform for photothermal therapy. Importantly, the fluorescence images (Figure 12d) showed almost complete cell death only in the BKP/ (1,2‐diaminocyclohexane)platinum(II) (DACHPt) + NIR group, while the BKP/DACHPt or NIR alone had significantly weaker effects, indicating a clear synergistic chemo‐photothermal mechanism. It is worth mentioning that the work achieved an ultra‐high drug loading efficiency exceeding 200%, showing the drug delivery potential of phosphorene. Furthermore, MTT assay (Figure 12e) revealed a concentration‐dependent cytotoxicity of BP/DACHPt with and without NIR, while Figure 12f showed that the cytotoxic effect increased with incubation time due to the sustained release of the active drug species. These results validate the dual therapeutic efficacy and long‐term functional stability of the BP‐based nanocomposite.^[^ ^142^ ^]^ The work of Yang et al. also confirmed this.^[^ ^149^ ^]^ Hydrogel‐embedded BKP phosphorene was prepared to carry polydopamine, which exhibited more than twice the drug‐loading capacity compared to pure hydrogels. Leveraging tunable optical properties and high drug‐loading efficiency, phosphorene enables synergistic chemo‐photothermal therapy with strong biodegradability and minimal toxicity. While functionalization is required to overcome oxidation, recent in vivo and in vitro studies validate its dual‐mode efficacy and sustained‐release behavior for biomedical use.

#### 3.3.2 Arsenene

##### Synthesis—Bottom‐Up Method

Arsenic, the fourth‐period element in group VA, has the same valence electronic structure as phosphorus. The 2D allotrope of arsenic, known as arsenene, has been predicted to exist in four structural phases. Among them, α‐arsenene has a pleated washboard geometry whose structure has a shape similar to that of a chair;^[^ ^150^ ^]^ β‐arsenene has a hexagonal honeycomb structure, with arsenic atoms arranged up and down like phosphorene,^[^ ^121^ ^]^ giving it a buckling structure;^[^ ^151^ ^]^ γ‐arsenene has a rectangular unit cell^[^ ^152^ ^]^ and λ‐arsenene with a three‐layer spatial structure, and its degree of buckling is greater than that of α‐arsenene.^[^ ^153^ ^]^

**Figure 13:**
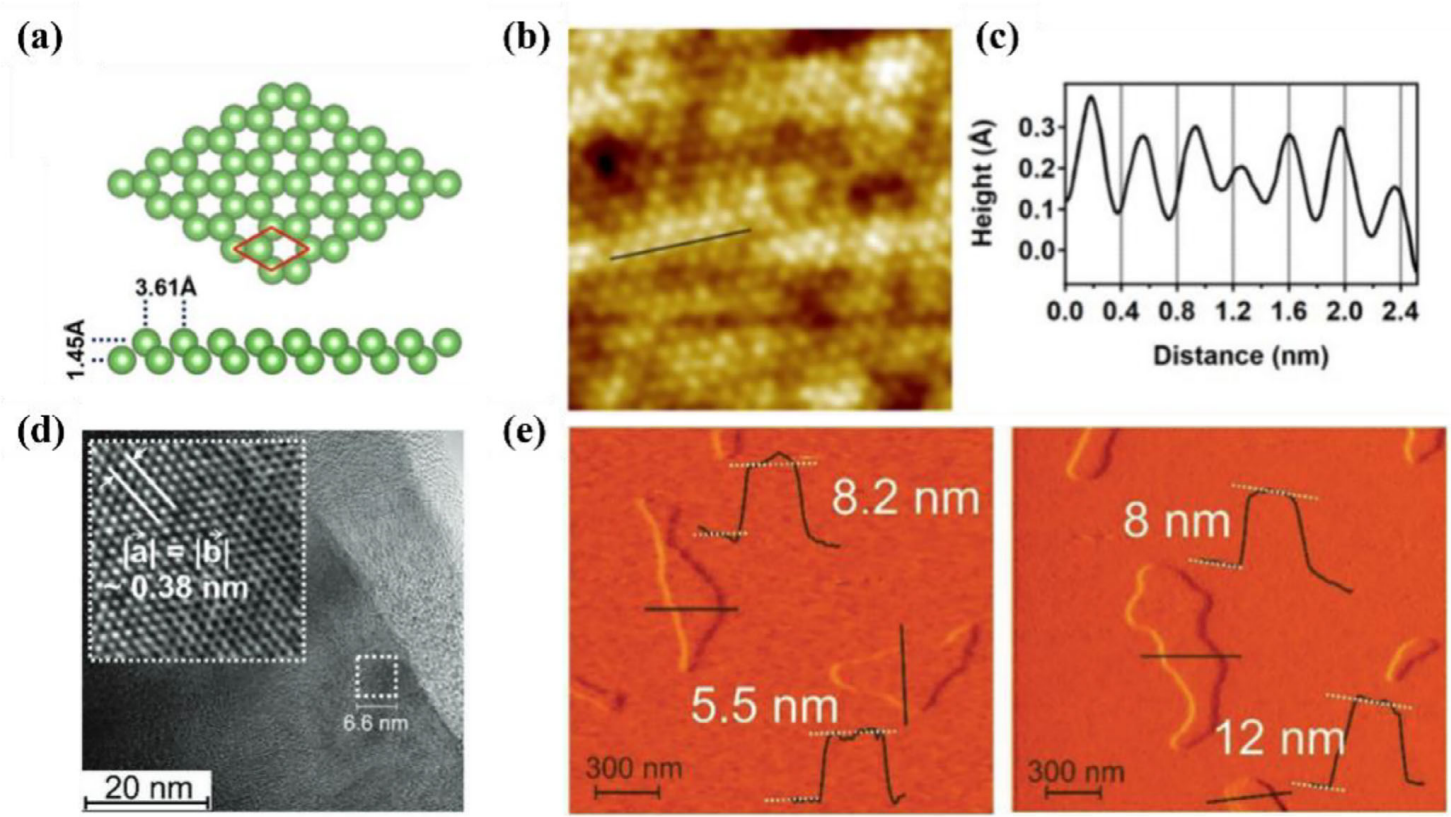
Image of lattice structure and experimental characterization of arsenene. a) Lattice structure (top and side view) of β‐arsenene; b) Atomically resolved empty state (10 mV) STM image; c) Line profile along the black line in (b), the average lattice constant is 3.6 Å, which is consistent with buckled arsenene; Reproduced with permission.^[^ ^130^ ^]^ d) HR‐TEM image of few‐layered arsenene. The inset is the zooming up of the white rectangle part; e) AFM morphology of arsenene shows several layered arsenene flakes of different heights. Reproduced with permission.^[^ ^131^ ^]^ Copyright 2019, John Wiley and Sons.

MBE method was used to synthesize arsenene. Under UHV conditions, Ag (111) was used as substrate after Ar^+^ sputtering and annealing at 400 °C. The sublimated As in heated InAs sheets can be deposited on the substrate surface to form arsenene at 250–350 °C. The wrinkled hexagonal honeycomb structure of arsenene shown in **Figure** **13**a is also consistent with other studies. The morphology study of arsenene (Figure 13b,c) demonstrated the average lattice constant as 3.6 Å, which is consistent with the data obtained by low‐energy electron diffraction (3.61 Å).^[^ ^130^ ^]^

##### Synthesis—Top‐Down Method

There are no vdWs forces between the layers of arsenic atoms. On the contrary, the larger spacing between the atoms of arsenic results in its anisotropic properties. The large atomic distance between the arsenic layers (64 pm) leads to the anisotropic behavior of this layered material, making top‐down exfoliation possible.^[^ ^131^ ^]^ Based on this principle, Beladi‐Mousavi et al. tried to use the LPE method to separate the fewer‐layer arsenic nanosheets based on ultrasonication.^[^ ^131^ ^]^ In this experiment, arsenic nanosheets after argon purging treatment were prepared by 30 W ultrasonication for 90 min using the oxygen‐free organic solvent (e.g., NMP). It is worth noting that the heating of the material needs to be prevented by 40‐s intervals of ultrasonic treatment and ice bath treatment. After one day of rest, centrifuge at 2000 rpm for half an hour to separate arsenic nanosheets. Figure 13d shows an exfoliated arsenene HR‐TEM image, illustrated with an enlarged image of the white square in the figure. The atomic force microscopy (AFM) image of the stripped arsenene is shown in Figure 13e, with different thicknesses ranging from 5 to 12 nm. The structure of arsenene obtained in this experiment shows excellent crystallinity and layered structure, which is consistent with the morphology of β‐arsenene (shown in Figure 13a), indicating that the LPE method retains the crystal structure of arsenic.

**Figure 14:**
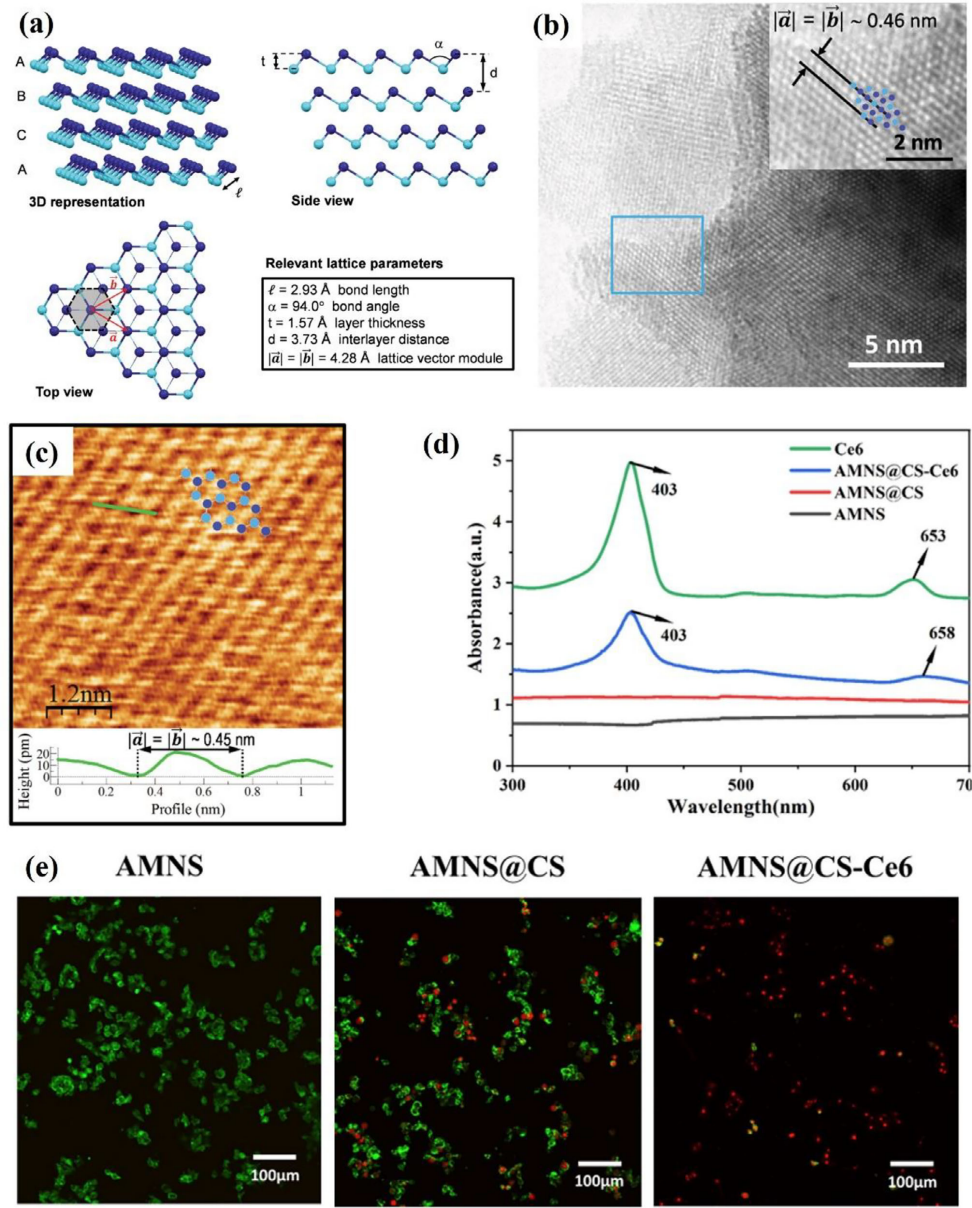
Structural characterization and photodynamic therapeutic applications of antimonene. a) Structure of antimonene, including three‐dimensional representation, side view, and top view with relevant lattice parameters; b) HR‐TEM image of antimonene. The inset is a zooming up of the blue rectangle; c) AFM topography of single‐layer antimonene. The superimposed portion of the image shows the atomic structure of antimonene (dark blue balls are the top atom, and light blue balls are the bottom atom). The bottom represents the profile of the green line in the AFM image. Reproduced with permission.^[^ ^133^ ^]^ Copyright 2016, John Wiley and Sons. d) The UV–vis spectra of Antimonene nanosheets (AMNS), AMNS@ chitosan coating (CS), AMNS@CS‐ chlorin e6 (Ce6), and free Ce6; e) The cell viability/cytotoxicity staining images show the state of the cells after being exposed to radiation for 5 min following their co‐incubation with AMNS, AMNS@CS, and AMNS@CS‐Ce6.; Reproduced with permission.^[^ ^166^ ^]^ Copyright 2024, Springer Nature.

##### Properties and Potential Applications of Arsenene—Energy Storage

Arsenic, which shares the same stoichiometric interlayer as phosphorus, has already been studied and demonstrated as a potential anode material in ion batteries. In traditional Li_3_As LiBs, the theoretical capacity of Li_3_As is 1072 mAh g^−1^, indicating its strong potential as an anode material for LiBs.^[^ ^154^ ^]^ Lim et al. use As/carbon nanocomposites as the anode of the LiBs, which has a reversible capacity of 1306 mAh g^−1^ after 100 cycles.^[^ ^154^ ^]^ The superiority of arsenene in energy storage is expected. However, the application of arsenene to energy storage is currently based on theory. A first principle calculation based on DFT for the α and β phases of arsenene shows that the diffusion barrier of β‐arsenene is slightly lower than that of α‐arsenene.^[^ ^155^ ^]^ The diffusion barrier for Li (0.11 eV and 0.12 eV), Na (0.04 eV and 0.07 eV), and K (0.03 eV and 0.05 eV)^[^ ^155^ ^]^ was better than that for some other 2D materials, such as graphene, black or blue phosphene.^[^ ^8^, ^145^ ^]^ Another study based on DFT calculations explored the possibility of using arsenene thin films as anchoring materials for the cathode in lithium‐sulfur batteries.^[^ ^156^ ^]^ With ultra‐low diffusion barriers for Li, Na, and K ions, arsenene shows strong theoretical promise for energy storage. While bulk arsenic‐based anodes have demonstrated high capacity experimentally, the application of 2D arsenene remains at the computational stage, awaiting synthesis and device‐level validation.

##### Properties and Potential Applications of Arsenene—Photovoltaics and Photocatalysis Fields

As a material with a 1.66 eV band gap, high carrier mobility (10^2^–10^4^ cm^2^ V^−1^ s^−1^), and excellent light absorption capacity, arsenene shows exciting potential in the field of photovoltaics and photocatalysis. Based on DFT calculations, the pairing of arsenene with molybdenum disulfide, tetracyano‐quinodimethane, or tetracyanonaphtho‐quinodimethane can form heterostructures with type II band arrangements.^[^ ^157^ ^]^ Such materials meet all the requirements for photocatalytic water decomposition, and as potential photovoltaic materials, they can exhibit excellent power conversion efficiency of ≈20%. Molecular dynamics simulations confirmed that arsenene interacts with H_2_O or O_2_ at room temperature via physisorption, and arsenene has a high energy barrier for oxidation, showing its high stability in ambient atmospheres.^[^ ^157^ ^]^ In addition, it has been reported that arsenene nanosheets induce apoptosis on NB_4_ acute promyelocytic leukemia cells (82% inhibition rate) without toxicity to normal cells.^[^ ^158^ ^]^ Arsenene combines a suitable band gap, high mobility, and ambient stability, fulfilling key criteria for photocatalysis and photovoltaics. Its biocompatibility and selective cytotoxicity further expand its potential toward multifunctional optoelectronic and biomedical applications.

#### 3.3.3 Antimonene

##### Synthesis—Bottom‐Up Method

As with other 2D materials, bottom‐up methods are actively being explored by researchers in pursuit of large‐area antimonene synthesis. Fortin‐Deschênes et al. chose the MBE technique to synthesize antimonene on the surface of Ge (111).^[^ ^132^ ^]^ A Ge (111) substrate cleaned with acetone and isopropyl‐ketone was blown dry with nitrogen and annealed for one hour at 600 to 700 °C under UHV conditions before flash firing to 800 °C. Antimonene was obtained by evaporating high‐purity antimony crystals (99.99999%) at a rate of 700 Å min^−1^ and controlling the growth temperature from room temperature to 330 °C. However, the obtained product contains a three‐dimensional antimony structure. In order to optimize this step, a second‐step growth process at a lower rate (below 50 Å min^−1^) was performed to control the morphology and quality of the grown antimonene. This process allows 2D islands to grow laterally faster than three‐dimensional islands, with no additional nucleation occurring, thus increasing the coverage of 2D islands relative to three‐dimensional islands.^[^ ^132^ ^]^ The thickness of antimonene ranges from 2 to 7 layers, with a single layer being ≈5.24 Å thick. The wrinkles are obvious and the size can reach several micrometers. Considering the possible effect of substrate on the growth of antimonene, the researchers explored more synthetic routes. At present, antimonene has been successfully synthesized by using Ag (111), Pb (111), Cu (111), and Cu (110) as substrates, which brings new possibilities for the study of antimonene.^[^ ^159^, ^160^, ^161^ ^]^

##### Synthesis—Top‐Down Method

Arsenic, as a heavy pnictogens (group VA element), retains a layered structure analogous to that of BKP. The vdWs interactions between layers make mechanical exfoliation of arsenic into 2D asenene possible.^[^ ^162^ ^]^ Theoretical studies of antimonene suggested a variety of phases, including α‐antimonene with a twisted bilayer structure, β‐antimonene with a hexagonal structure similar to the surface of metal Sb (111), and γ‐antimonene and δ‐antimonene with rectangular Wigner‐Seitz units.^[^ ^163^ ^]^ The first experimental synthesis of antimonene was reported by Ares et al. in 2016.^[^ ^133^ ^]^ This work used an adhesive tape to exfoliate antimony tablets from commercially available high‐purity block antimony (99.99999%). To avoid the inefficiencies caused by direct transfer, the researchers used a more elaborate strategy of transferring the antimony sheet from the tape to a layer of viscoelastic polymer attached to the glass sheet. By pressing the polymer onto the silicon oxide substrate, the researchers were able to obtain a large area of thin antimony sheets (antimonene) in a controllable manner, which was visible through an optical microscope (OM). **Figure** **14**a shows the molecular structure of β‐antimonene, which is confirmed in the HR‐TEM image shown in Figure 14b. Meanwhile, the high‐resolution AFM images taken after two months in the atmosphere are shown in Figure 14c. The shape of the green line is shown at the bottom of the image, and the blue rhombus shows the atomic lattice of antimony atoms (dark blue is the top atom, light blue is the bottom atom, and the side length is 1.284 nm). The monolayer antimonene is 0.4 nm, with a hexagonal lattice structure and a good crystal structure. This result has also been verified through DFT calculation simulation. After several months of observation and nano‐manipulation, the stability of antimonene was further confirmed by AFM, OM, and TEM.^[^ ^133^ ^]^ Subsequently, the LPE method was also successfully applied to the synthesis of antimonene, and the obtained antimonene showed the same characteristics as mechanical exfoliation.^[^ ^164^, ^165^ ^]^ A novel top‐down approach has been reported in a recent study. This work presented a pressurized alloying method for introducing Li_3_Sb alloy intermediates by pre‐physicochemical β‐phase antimony edge regions in the presence of n‐butyllithium and internal pressure. The reaction of Li_3_Sb with deionized water produces gaseous SbH_3_, and the gas buoyancy provided by this process instantaneously overcomes the interlayer vdWs forces to promote the isolation of β‐antimonene. The result shows that β‐phase antimony can be efficiently exfoliated into antimonene nanosheets with perfect base texture (transverse size about 3 µm, thickness less than 2 nm, and wrinkled hexagonal lattice structure) by this method.^[^ ^134^ ^]^

##### Properties and Potential Applications of Antimonene—Energy Storage

Similar to phosphene, antimonene has attracted significant attention in the field of energy storage due to the excellent Na^+^ theoretical storage capacity of antimony (660 mAh g^−1^).^[^ ^167^ ^]^ After the successful synthesis of antimonene, Tian et al. studied the Na^+^ storage process of the few‐layer antimonene. They found that few‐layer antimonene has a diffusion barrier of 0.14 eV, which is extremely favorable for Na^+^ diffusion. According to in‐situ synchronous XRD, ex‐situ selected‐area electron diffraction, and DFT calculations, antimonene underwent a reversible crystal phase evolution to $\mathrm{Sb}⇋\mathrm{NaSb}⇋Na_{3}\mathrm{Sb}$, which realizing high structural stability storage of Na^+^.^[^ ^168^ ^]^ In addition, the energy storage behavior of antimonene electrodes in liquid electrolytes is also excellent. One work reported a specific capacitance up to 599 F g^−1^ by scanning antimonene electrodes in H_2_SO_4_, which is more than twice the capacitance achieved in KOH, LiOH, and LiCl‐based electrolyte systems.^[^ ^169^ ^]^ Using an asymmetric supercapacitor composed of a positive antimonene electrode and a negative carbon nanotube electrode, researchers demonstrated a wide operating voltage of 1.8 V, a relatively high energy density of 46 Wh kg^−1^ at a power density of 450 W kg^−1^ and excellent cycling stability (after 5000 cycles, capacitance retention reaches 112.7% at 1.5 A g^−1^).^[^ ^169^ ^]^ Antimonene exhibits favorable Na⁺ diffusion kinetics, reversible phase transitions, and high electrochemical stability, enabling efficient ion storage and supercapacitor performance. Its strong capacitance response in acidic electrolytes and robust cycling behavior highlight its versatility across battery and hybrid energy storage platforms.

##### Properties and Potential Applications of Antimonene—Optoelectronic and Electronic Devices

It has been shown that antimonene can absorb light in the visible range and has favorable carrier mobility, which makes it potentially useful in optoelectronic and electronic devices. Zhang et al. demonstrated that antimony behaves as a semimetal in the block state.^[^ ^170^ ^]^ However, when antimonene is tinned to a single atomic layer, it becomes an indirect semiconductor with a band gap of 2.28 eV. This transition has important implications for the development of transistors with high switching ratios, optoelectronic devices operating in the blue to ultraviolet spectral range, and mechanical sensors based on novel 2D crystals. Wang et al. used ab initio molecular dynamics simulations to study the properties of monolayer β‐antimonene and obtained an optical bandgap of 1.5 eV and electron/hole mobility of 150/510 cm^2^ V^−1^ s^−1^.^[^ ^171^ ^]^ They also optimized the structure of the antimonene FETs through DFT and generalized gradient approximation to meet the low‐power and high‐performance requirements outlined in the international semiconductor technology roadmap for the coming decade. While antimonene exhibits tunable electronic structure and theoretical suitability for optoelectronic applications, current studies remain largely computational. The absence of device‐level validation and challenges in material stability highlight the gap between conceptual promise and practical implementation.

##### Properties and Potential Applications of Antimonene—Biomedical Fields

The optical properties of antimonene have also sparked interest in its use in biomedical photothermal therapy. Fickert et al. has studied the thermophysical behavior of antimonene using temperature‐dependent and laser power‐dependent Raman spectroscopy, combined with numerical simulations of heat transfer mechanisms conducted in COMSOL Multiphysics.^[^ ^172^ ^]^ The results showed that antimonene has photothermal oxidation behavior and is expected to be applied in the field of photothermal therapy. However, the inherent toxicity of antimony and the current limited understanding of the biocompatibility of antimonene pose challenges to its biomedical applications. Therefore, functionalization is a necessary condition for improving biocompatibility and promoting safe therapeutic use. In one work, chitosan/antimonene hydrogels (CS/AM hydrogels) were prepared by using hydrogels as additional carriers. Through vitro antibacterial tests, the antibacterial effect of CS/AM hydrogels was significantly enhanced under near‐infrared light irradiation. A mouse model was used to detect the wound healing and antibacterial performance of CS/AM hydrogels both in vivo and in vitro, and the results proved that CS/AM hydrogels effectively promote wound healing and have significant antibacterial activity. In addition, biodistribution analysis revealed no detectable accumulation of antimonene in major organs, and the materials showed low in vivo toxicity.^[^ ^173^ ^]^ Recent advancements have pushed the biomedical applications of antimonene further by integrating surface functionalization and combinatorial therapeutic strategies. For instance, Zhang et al. developed a multifunctional nanoplatform by modifying antimonene nanosheets with chitosan and loading them with chlorin e6 (Ce6), enabling both photothermal therapy (PTT) and photodynamic therapy in a synergistic manner.^[^ ^166^ ^]^ UV–vis spectra verified the preservation of Ce6's photodynamic functionality after conjugation, evidenced by the characteristic absorption peaks at 403 and 658 nm as shown in Figure 14d. To evaluate in vitro therapeutic efficacy, Calcein‐AM/PI staining was performed under dual‐laser irradiation, focusing on three treated groups (Figure 14e). Cells treated with unmodified AMNS showed partial cell death, indicating limited photothermal effects. Upon chitosan coating (AMNS@CS), red fluorescence increased, reflecting enhanced thermal stability and improved PTT efficacy. Notably, AMNS@CS‐Ce6 induced almost complete cell death, demonstrating a pronounced synergistic effect of photothermal and photodynamic therapy.^[^ ^166^ ^]^ Thus, functionalized antimonene‐based nanoplatforms represent a promising direction for precise, biocompatible, and minimally invasive cancer therapy.

#### 3.3.4 Bismuthene

##### Synthesis—Bottom‐Up Method

**Figure 15:**
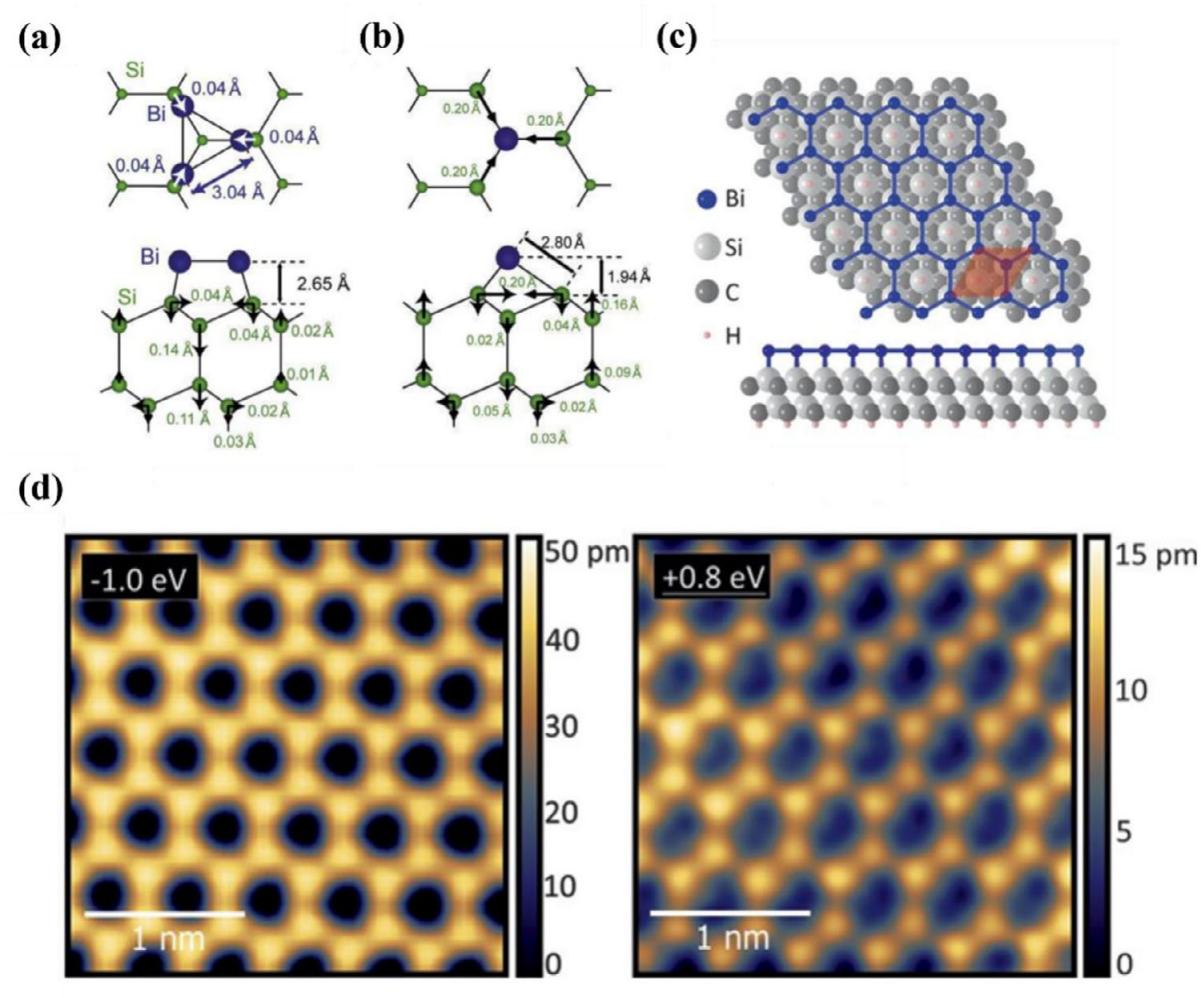
Structural model of the a) β‐phase and b) α‐phase of bismuthene; Reproduced with permission.^[^ ^174^ ^]^ Copyright 2010, Elsevier; c) A schematic representation of a bismuthene monolayer epitaxially aligned on SiC (0001) substrate with a commensurate (√3×√3) R30° superlattice registration; d) HR‐STM images depicting both the occupied (left) and unoccupied (right) electronic states are provided. These images corroborate the emergence of bismuth‐constructed hexagonal lattice structures. Reproduced with permission.^[^ ^4^ ^]^ Copyright 2017, American Association for the Advancement of Science.

As the heaviest element of Group VA, 2D structure of bismuth has been expected in terms of electronic properties, and the exploration of 2D bismuth (bismuthene) has been reported. Kuzumaki et al. used dynamic low‐energy electron diffraction to study the atomic structure of Bi/Si (111)–(√3×√3) R30° reconstructed surface formed under different adsorbent coverage. However, the I‐V curve formed on the α‐Bi/Si (111)–(√3×√3) R30° surface is inconsistent with earlier low‐energy electron diffraction studies.^[^ ^174^ ^]^ This discrepancy is thought to be due to the low quality of the sample and the co‐existence of a portion of the β‐phase bismuthene (**Figure** **15**a) with the α‐phase (Figure 15b). The successful synthesis of the expected bismuthene was reported in 2017. This synthesis process in this work is performed under UHV conditions using a smooth SiC (0001) with H‐terminated as substrate. Before growing bismuthene, it is necessary to heat to ≈650 °C to slowly desorption H on the substrate. During this process, researchers provided Bi atoms to achieve bismuth deposition and, finally, annealing at ≈400 °C to improve the orderability of the honeycomb film.^[^ ^4^ ^]^ The synthesized bismuthene was shown to have a honeycomb structure, as shown in Figure 15c,d, with a lattice constant of 5.35 Å. On the basis of previous research, recent work has achieved the use of low‐temperature and low‐pressure hydrochemical etching combined with MBE technology to establish a convenient method to prepare bismuthene (β‐phase with honeycomb structure) selectively and α‐Bi on hydrogen‐terminated SiC (0001).^[^ ^175^ ^]^

##### Synthesis—Top‐Down Method

Due to the layered structure of bismuth, the top‐down method that overcomes interlayer vdWs forces are also being explored for bismuthene synthesis. Lu et al. proposed a method for the synthesis of the few‐layer bismuthene with a rhomboid A7 structure by sonochemical exfoliation.^[^ ^135^ ^]^ High‐purity bismuth (99.999%) was ground into powder as raw material. The mixture of bismuth and isopropyl was treated with ice bath ultrasound and probe ultrasound, and finally centrifuged at 5000 rpm for 20 min. The characterization results of TEM, SEM, and AFM confirmed that the thickness of the β‐bismuth suspension prepared by sonochemical exfoliation was 4 nm, the lattice spacing was 0.322 nm, and it presented a honeycomb structure. In another work, bismuthene nanosheets were synthesized by an ionic liquid‐assisted grinding and exfoliation method.^[^ ^136^ ^]^ This work ensured adequate peeling by grinding bulk bismuth powder and ionic liquid (1‐butyl‐3‐methylimidazolium hexafluorophosphate, BMIMPF_6_) over a long period (60 h). After washing with DMF‐acetone mixture to remove the ionic liquid, the product was dimensioned by a sequential centrifugation step and dried under vacuum to obtain the bismuthene product. Bismuthene obtained by centrifugation at 3000 rpm has a transverse size of 20nm and a thickness of 2nm, and its lattice structure retains the rhombohedral lattice structure of bulk bismuth

##### Properties and Potential Applications of Bismuthine—Energy Storage

Bismuth has high theoretical capacity and excellent alkali metal adsorption energy, which are ideal characteristics to be used as anode materials for ion batteries. Bi has a theoretical volumetric capacity of up to 3800 mA h cm^−3^, while maintaining a capacity retention rate of 94.4% after 2000 cycles in battery with NaPF_6_‐diglyme electrolyte.^[^ ^176^ ^]^ A first‐principles study calculated the potential of buckled bismuthine as an anode material for lithium, sodium, and potassium ion batteries.^[^ ^177^ ^]^ The high theoretical capacity of buckled bismuthene (2276 mAh g^−1^, 2149 mAh g^−1^, and 1896 mAh g^−1^, respectively) and good adsorption stability (adsorption energies with 2.7 eV, 2.94 eV, and 3.45 eV, respectively) demonstrate the great potential of buckled bismuthene materials for battery anodes. Another study based on first‐principles calculations found that bismuthene can adsorb up to two layers of potassium atoms, and the electronic conductivity of bismuthene after adsorption is significantly improved. The ultra‐low average open‐circuit voltage (0.17 V) along the zigzag direction of the bismuthene monolayer combined with the low diffusion barrier to potassium atoms (0.02 eV) suggests that the bismuthine‐based batteries have excellent performance with high energy density and excellent rate performance.^[^ ^178^ ^]^ With strong metal adsorption, high theoretical capacity, and low diffusion barriers, bismuthene shows promise for next‐generation alkali‐ion batteries. Its structural features support fast charge transport and high energy density. However, translating these properties into scalable devices requires further validation of cycling stability, conductivity tuning, and material‐process integration.

##### Properties and Potential Applications of Bismuthine—Topological Insulator

Bismuth has the largest atomic number in Group VA and also has a large bulk gap up to ≈0.8 eV,^[^ ^4^ ^]^ which is the largest bulk gap identified so far in the experiment. It also provides bismuthene with the ability to have a strong SOC. As early as 2006, Murakami theoretically predicted that bismuthene has a 2D topological insulator state.^[^ ^179^ ^]^ As mentioned earlier in this review, topological insulator materials have many excellent properties.^[^ ^116^ ^]^ The work of Reis et al. calculated the topological edge states of bismuthene by using a compact bound model and DFT simulations.^[^ ^4^ ^]^ They proposed a strong SOC resulting from the interaction of bismuthene and SiC substrate and determined that the Rashba‐type SOC introduces a band splitting of 0.384 eV. Bismuth has proved to be an ideal candidate for QSH insulators with spin conductivity in topological insulators. Despite its promising QSH behavior and strong SOC, current studies on bismuthene remain largely theoretical or substrate‐dependent, with limited scalability and experimental validation, warranting further work on growth control, stability, and device integration.

##### Properties and Potential Applications of Bismuthine—Photonic Fields

Bismuth is also regarded as a promising material for photonic applications because of its unique electronic properties, intrinsic stability, and nonlinear optical properties. The optical properties of a few‐layer bismuthene with a rhomboid A7 crystal structure were studied in previous research.^[^ ^135^ ^]^ The results showed that the slope of the scattering ring correlates with the decrease of light intensity as the wavelength increases, indicating wavelength‐dependent scattering behavior. This phenomenon indicates that spatial self‐phase modulation is mainly caused by electronic transitions. At the same time, the saturation absorption characteristic of bismuthene was found, the light modulation depth was 2.03%, and the saturation intensity was about 30 MW cm^−2^. The direct modulation and generation of ultrafast pulsed laser at 1559.18 nm in the NIR band is realized by using a saturation absorber based on a few‐layer bismuthine.^[^ ^135^ ^]^ Guo et al. fabricated a Sub‐200 femtosecond (fs) soliton mode‐locked erbium‐doped fiber laser based on microfiber saturated absorber using bismuthene obtained by sonochemical exfoliation method. The researchers measured the operating characteristics of the laser when only microfibers were inserted, from which only continuous wave lasers could be emitted. After inserting bismuthene microfibers, stable soliton pulses with Kelly sideband can be obtained when the pump power is increased from 100 to 350 mW.^[^ ^180^ ^]^ The soliton laser has a pump threshold of 50 mW at 976 nm and a pulse width of about 193 fs, which is the shortest soliton pulse produced by a laser made from 2D materials based on elements of Group VA up to the time of this experiment. While bismuthene‐based saturable absorbers show strong potential for ultrafast photonics, challenges remain in achieving large‐scale, uniform exfoliation and long‐term stability, and current demonstrations are mostly limited to lab‐scale fiber systems with few comparative benchmarks.

### 3.4 Group VIA

In group VIA, only the 2D structures of selenium and tellurium were experimentally prepared. And unlike other 2D materials, their 2D structure is formed by the arrangement of helical chains in a specific direction.^[^ ^29^, ^181^ ^]^ The preparation and morphological characteristics of selenene and tellurene are shown in **Table** **4**.

**Table 4:** Xenes of Group VIA elements.

Xenes	Precursor	Synthetic method	Morphological characteristics	Refs.
Selenene	Se powder	PVD	Minimum thickness of 5 nm, a serrated edge structure, hexagonal lattice structure with helical chain stacking	[35]
	Se powder	CVT	Ultra‐thin (0.6 to 0.7nm) nanosheets, hexagonal lattice structure with helical chain stacking	[182]
	Bulk Se	LPE	Size of 50–130 nm, thickness of 5–10 nm, triclinic selenium phase	[183]
Tellurene	Te powder	PVD	Hexagonal tellurene, thickness of 77 nm, size of 6–10 µm	[29]
	Na_2_TeO_3_	LPE	Thickness of 10 nm, size up to 100 µm, unique helical chain‐like structure	[30]
	Te	Mechanical exfoliation	Thickness of 15 nm, length up to 50 nm	[28]
	Te	LPE	Thickness in a range of 5.1 to 6.4 nm, size in a range of 41.5 to 177.5 nm, lattice distance of 3.2 Å	[184]

#### 3.4.1 Selenene

##### Synthesis—Bottom‐Up Method

**Figure 16:**
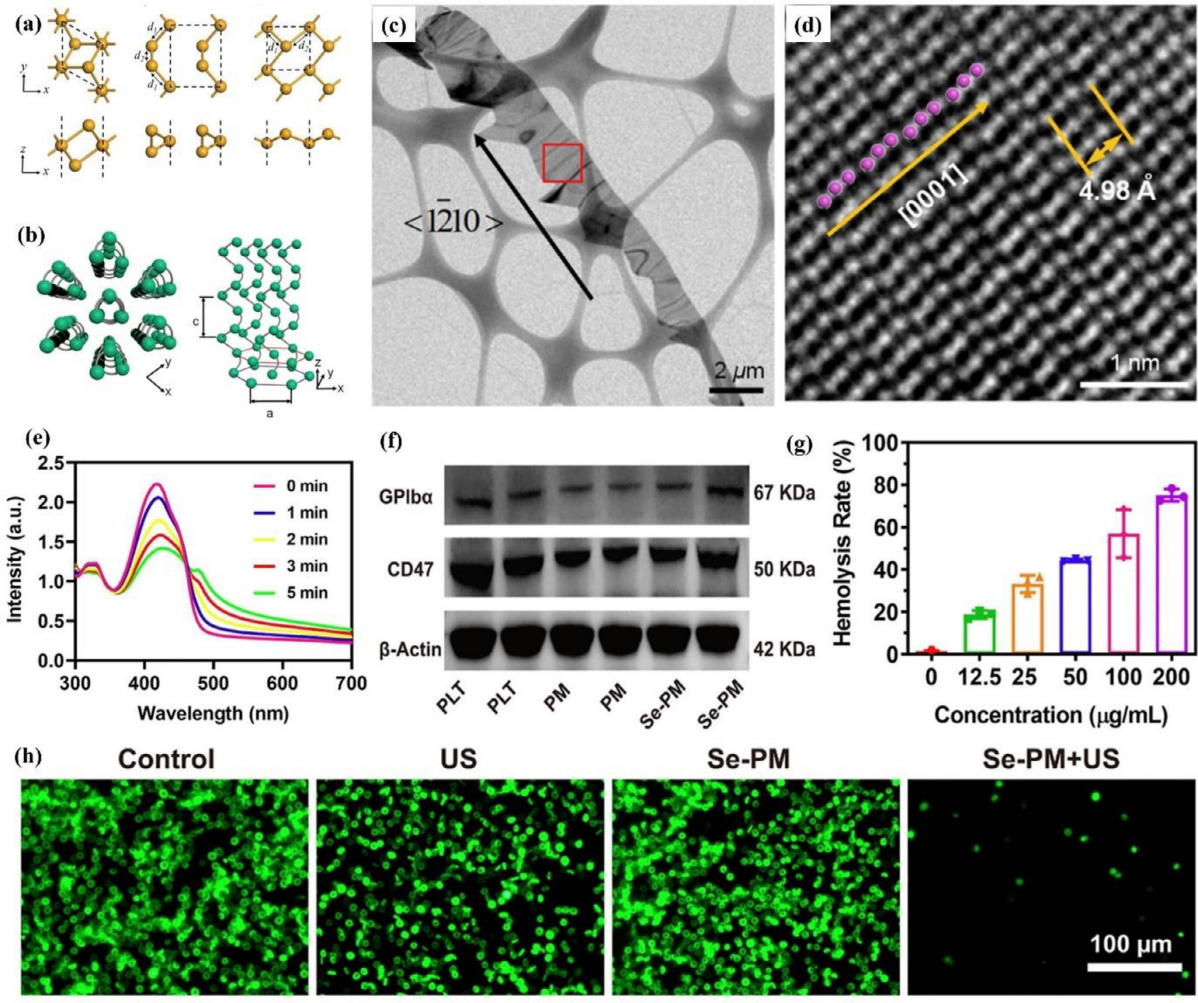
Structure diagram and experimental characterization diagram of selenene. a) Top and side views of three structures of selenene: T‐Se (left), C‐Se (middle), and S‐Se (right). Black dashed lines indicate the primitive cell of each phase. The bond length on the left is 2.67 Å; the middle is *d1* = 2.40 Å, *d2* = 2.37 Å, and the right *d1* = 2.40 Å, *d2* = 2.71 Å; Reproduced with permission.^[^ ^181^ ^]^ Copyright 2019, Institute of Physics Publishing. b) Atomic structure of T‐Se, include top view and side view; c) Bright‐field TEM characterization of a Se nanosheet; d) High‐angle annular dark‐field scanning transmission electron microscopy image. Reproduced with permission.^[^ ^35^ ^]^ Copyright 2017, American Chemical Society. e) Time‐dependent degradation of methylene blue under ultrasound irradiation with selenene, indicating •OH generation; f) Western blot of (Glycoprotein Ib alpha chain) GPIbα and CD47 (a transmembrane protein) in platelets, platelet membrane, and Se‐Platelet membrane nanosheets; g) Hemolysis rate of Se‐Platelet membrane nanosheets under ultrasound at different concentrations; h) Fluorescence images of red blood cells stained with calcein‐AM under different treatment conditions (control, ultrasound, Se‐platelet membrane, and Se‐platelet membrane with ultrasound); Reproduced with permission.^[^ ^185^ ^]^ Copyright 2024, Elsevier.

Through a particle swarm optimization algorithm combined with DFT calculations, three stable structures of 2D selenium were identified, as shown in **Figure** **16**a.^[^ ^181^ ^]^ T‐Se has a structure similar to 1T‐MoS_2_, C‐Se consists of a series of one‐dimensional helical chains, and S‐Se has a square structure. A bottom‐up synthesis method has been reported for the successful synthesis of 2D T‐Se (selenene).^[^ ^35^ ^]^ As shown in Figure 16b, selenium atoms are covalently bonded along the c‐axis in the form of helical chains, which are stacked radially along their axis through weak vdWs interactions to form a hexagonal lattice structure. In this work, the researchers used the PVD method to successfully synthesize large‐size high‐quality 2D selenium nanosheets. The silicon single wafer (Si (111)) is used as the growth substrate and is placed 20–25 cm away from the high‐purity selenium powder (99.99%). The temperature of selenium source area is controlled at 210 °C, and the temperature of base is 100 °C under argon environment. The researchers found that amorphous selenium nanospheres appeared on the substrate surface when the temperature rose to 160 °C and finally formed ultra‐thin selenium nanosheets with a minimum thickness of 5 nm in 60 min after the target temperature was reached. As shown in Figure 16c, the obtained bright field TEM image of selenene shows that it has a serrated edge structure, and the high‐angle annular dark field scanning transmission electron microscopy image (Figure 16d) shows the analysis of Se helix atomic chains along [0001] with a lattice surface fringe spacing of 4.98 Å.^[^ ^35^ ^]^

Recent work has also produced selenene by assisted growth of metal seeds using chemical vapor transport (CVT).^[^ ^182^ ^]^ In this work, silicon or Si/SiO_2_ substrates are used as the growth substrate. After a cleaning and drying step, bismuth, tin, or antimony metals are used as seeds in ethanol for ultrasonic probe stripping to obtain thin metal sheets. These metal sheets are then applied to the clean base by drip coating. CVT growth was performed using a single‐zone hot furnace. The base is placed in the center of the heating zone, and the selenium powder is placed at the upstream end of the tube furnace to ensure that the temperature is above the melting point of the selenium. Aron‐hydrogen (85%/15%) is used as the carrier gas, and the temperature needs to be heated to 750 °C. The temperature needs to be maintained for 10 min, and then the furnace needs to be cooled naturally to ensure the successful growth of selenene. AFM analysis showed that the bilayer selenene synthesized by this method has an ultra‐thin thickness.^[^ ^182^ ^]^ The successful synthesis of ultra‐thin (0.6–0.7 nm) selenene is a surprising improvement compared to the thicker selenene (5 nm) obtained in previous experiments.^[^ ^35^ ^]^ It is worth mentioning that the atomic chain structure of selenene synthesized in this experiment is helically arranged in the direction of (0001), forming a hexagonal lattice, which is different from the thick selenene nanosheets growing in the (0001) direction shown in Figure 16d. Also, the measured surface spacing at (010), (012), and (003) reflections were shown as 3.3, 2.0, and 1.7 Å, respectively.

**Figure 17:**
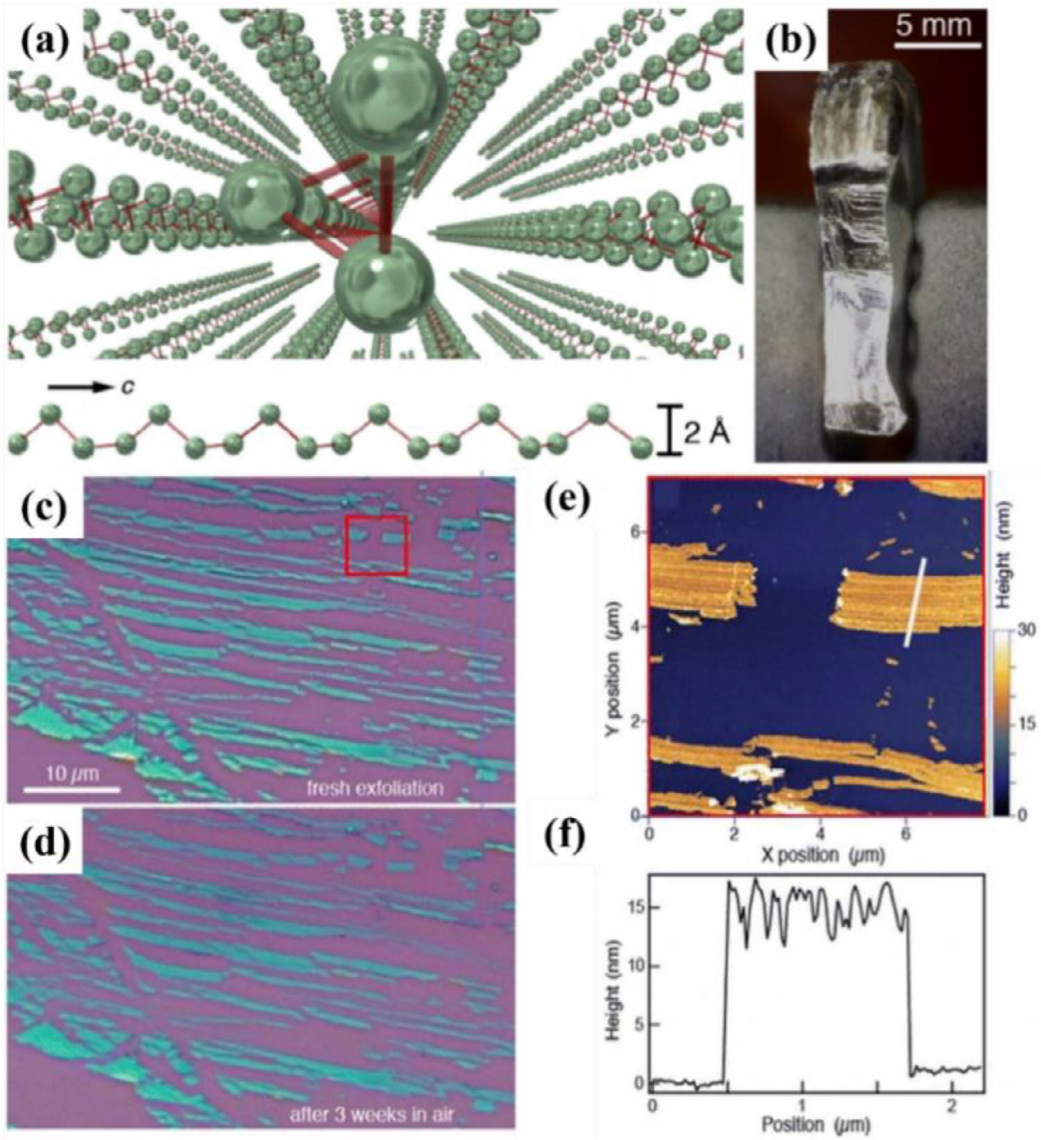
Te element and related characterization of 2D Te materials. a) A diagrammatic representation of a Te single‐crystal lattice composed of single‐atom chains held together by vdWs forces is depicted from the top view, with a side view of the Te chain configuration shown below. The triangular cross‐section of a chain measures 2 Å in height, and the distance between chains is 3.4 Å; b) Te single crystal used in synthesis of 2D Te experiment; c) 2D tellurene exfoliated on a Si/SiO_2_ substrate, captured immediately post‐exfoliation.; d) Same sample as in (c) following three weeks of air exposure; e) AFM image of the red square in (c); f) Height profile along the white line in (e). Reproduced under the terms of the CC‐BY license.^[^ ^28^ ^]^ Copyright 2017, Hugh O. H. Churchill et al.

##### Synthesis—Top‐Down Method

It is well established that layered bulk materials can be exfoliated to obtain 2D counterparts.^[^ ^9^, ^12^, ^54^ ^]^ However, as mentioned earlier, selenium does not exist a typical layered structure, instead it consists of atomic chains held together by vdWs forces.^[^ ^35^ ^]^ Fan et al. successfully synthesized non‐layered 2D selenium nanosheets by the LPE method.^[^ ^183^ ^]^ In their work, bulk selenium powder was used as raw material for LPE in IPA. After the bulk selenium powder is dispersed in IPA to form a sealed mixture, the mixture is ultrasonic treated with a probe ultrasonic processor, the power is set to 200 W, and the switching cycle is 2 seconds on/2 seconds off to avoid thermal oxidation during exfoliation. The ultrasound‐treated selenium dispersion was sealed and subjected to a bath ultrasound treatment for 10 hours, using a water‐cooled circulation system to keep the mixture temperature below 15 °C. The selenium dispersion is centrifuged using a centrifuge, which is first centrifuged at 3000 rpm for 30 minutes to remove larger selenium particles. Then, centrifuge again at 10000 rpm for 30 minutes to collect the product, and large area between 50 and 130 nm 2D selenium nanosheets were obtained after drying. Measured by AFM, the thickness of 2D selenium nanosheets is between 5 and 10 nm, and XRD and Raman spectroscopic analysis further confirms that the synthesized 2D selenium nanosheets are typical of triclinic selenium (T‐Se) phase.^[^ ^183^ ^]^

##### Properties and Potential Applications of Selenene—Photodetectors

Because of the unique atomic chain structure and 2D morphology, selenene exhibits excellent photoelectric properties, such as high photoconductivity, strong responsivity, and rapid response time. These characteristics make 2D selenium nanosheets have potential application value in the field of photodetectors.^[^ ^183^ ^]^ In the work of synthesizing selenene via the PVD method, FETs and phototransistors were fabricated using the synthesized selenene, and their device performance was evaluated.^[^ ^35^ ^]^ Selenene nanosheet phototransistors exhibit photoresponsivity up to 263 AW^−1^, which indicates that it is an efficient photoelectric material. The photocurrent response speed of selenene nanosheet phototransistor is also an important performance index. According to the report, the rise time of selenene nanosheet phototransistor is 0.1 s, and the fall time is 0.12 s, which indicates that selenene has a fast light response ability. In addition, the high stability of selenene under environmental conditions has become the key to the industrialization of this application. Even after 15 days of exposure to air, the surface morphology of selenene is almost unchanged, and the device performance is not significantly degraded.^[^ ^35^ ^]^ In addition, photodetectors based on photoelectrochemical are also being studied by researchers. Fan et al. dispersed selenene nanosheets obtained by LPE in the mixture of polyvinylidene fluoride and DMF by ultrasonic treatment, directly dropped the dispersion on the surface of conductive indium tin oxide glass after ultrasonic cleaning, and dried in vacuum as a working electrode.^[^ ^183^ ^]^ A three‐electrode system with platinum wire as the counter electrode and saturated calomel electrode as the reference electrode was constructed. The photoelectric response behavior was measured by linear sweep voltammetry and amperometry. The results show that the photocurrent density increases significantly with the increase of bias voltage in the range of 0 to −0.6 V. At −0.6 V bias voltage, the photocurrent density reaches 1.28 µA cm^−2^ and the photoresponse is 10.45 µA W^−1^, showing excellent photodetection performance.^[^ ^183^ ^]^ Although selenene shows impressive responsivity and environmental stability, its relatively slow response time and low photocurrent density in PEC systems highlight the need for improved charge transport strategies and better contact engineering for practical, high‐speed device applications.

##### Properties and Potential Applications of Selenene—Biomedical Fields

As selenium is an essential trace element in the human body, the potential biomedical applications of selenene have been explored. A recent study proposed the application of selenene in the field of piezothrombolysis.^[^ ^185^ ^]^ Piezothrombolysis is a therapeutic technique that uses the energy of ultrasound waves to break up and dissolve blood clots. This method typically integrates piezoelectric materials with ultrasonic stimulation, offering a non‐invasive method for targeted thrombolytic therapy. The mechanism of piezothrombolysis hinges on the piezoelectric nature of selenene. Under ultrasound (US) stimulation, selenene generates electron–hole pairs that react with H_2_O and O_2_ to produce reactive oxygen species (ROS), including hydroxyl radicals (•OH) and superoxide anions (•O_2_^−^). This ROS generation was directly verified via diphenyl isobenzofuran degradation and electron paramagnetic resonance signals, as shown in Figure 16e, which clearly demonstrates the effective production of •O_2_^−^ under ultrasonic irradiation in the presence of selenene. The generation of these ROS species is responsible for breaking fibrin skeletons and damaging erythrocyte membranes, thus enabling effective thrombus disintegration. To achieve biological targeting and systemic biosafety, the authors coated selenene with murine platelet membranes. As confirmed by western blot results in Figure 16f, essential membrane proteins such as CD47 and GPIbα were retained on the platelet membrane (PM) nanosheets, which ensuring the thrombus recognition and immune evasion. This functionalization allows for selective accumulation at clot sites, reducing off‐target effects and enhancing therapeutic precision.^[^ ^185^ ^]^ In terms of therapeutic performance, hemolysis assays showed a significant increase in erythrocyte lysis only when Se‐PM nanosheets were activated by ultrasound, reaching over 70% at higher concentrations (Figure 16g). This effect was further confirmed by calcein‐AM (calcein acetoxymethyl ester) staining (Figure 16h), where red blood cells exhibited strong fluorescence in control groups but showed fragmented or diminished signals after Se‐PM + ultrasound treatment, indicating membrane rupture. These results highlight the ultrasound‐triggered and localized thrombolytic activity of Se‐PM nanosheets. While selenene‐based piezothrombolysis shows promise for targeted, non‐invasive thrombolytic therapy, current studies are limited to in vitro validation; further investigation into in vivo biocompatibility, long‐term biosafety, and targeted delivery efficiency is needed for clinical translation.

#### 3.4.2 Tellurene

##### Synthesis—Bottom‐Up Method

Tellurium and selenium, both members of group VIA, have a helical chain structure. As a result, the early research on the growth of tellurium nanomaterials was predominantly focused on the synthesis of one‐dimensional nanostructures.^[^ ^186^, ^187^ ^]^ The synthesis of 2D tellurium nanoplates was proposed in 2014 in a report by Wang et al. .^[^ ^29^ ^]^ In this work, a vdWs epitaxy method was successfully used for the growth of 2D tellurene. Unlike traditional epitaxial growth methods, vdWs epitaxy relies on weak vdWs forces and uses chemically inert substrates. This method can effectively reduce the chemical reaction between the substrate and the growing material. In this work, fluorophlogopite mica (KMg_3_(AlSi_3_O_10_)F_2_) was used as the substrate. Tellurium powder (99.99% purity) and the substrate were placed in a vacuum tube in an argon environment. The furnace temperature was set to 750 °C for the tellurium source and 500 °C for the substrate and maintained for 60 min to promote the deposition and growth of tellurium atoms on the mica substrate. After growing, the tellurene on the substrate was cooled naturally to room temperature. The tellurium nanoplates grown on the mica substrate were transferred to ethanol by ultrasonic treatment for subsequent analysis.^[^ ^29^ ^]^ Hexagonal tellurium nanoplates were observed under an OM. However, the tellurium nanosheets obtained in this work are relatively thick, the transverse size is small (6–10 µm), and the synthesis of high‐quality tellurene is still being investigated.

Wang et al. proposed a solution‐based growth method for the synthesis of ultra‐thin, large‐area, and high‐quality 2D tellurene.^[^ ^30^ ^]^ Analytically pure NaTeO_3_ (4.5 × 10^−4^ moles) and a certain amount of polyethylene pyrrolidone into double distilled water were used as raw materials, mixed in 25wt% ammonia and 80wt% hydrazine hydrate. The reaction took place at a temperature of 160—200 °C for a period of time, and after cooling, the solution was centrifuged to obtain the precipitate and washed with double‐distilled water to obtain the product. In this work, a solvent‐assisted post‐growth refinement process was also proposed. By mixing the synthesized solution with acetone at a ratio of 1:3 for six hours and centrifuging, ultra‐thin tellurene with an average thickness of 10 nm and a size of 100 µm can be obtained. Tellurium atoms are arranged in the form of helical chains and stacked together through vdWs to form a hexagonal lattice as selenene.^[^ ^182^ ^]^ In the subsequent work, Wang et al. further explored the solution growth‐based methods for tellurene synthesis on, revealing the influence of different processing conditions on both the quality and yield of the resulting tellurene products.^[^ ^188^ ^]^ Tellurene was synthesized and characterized by Gao et al. using the same method, and the results of XRD and Raman spectra confirm that tellurene nanosheets have a hexagonal phase structure.^[^ ^189^ ^]^ In addition, the MBE method has also been successfully used to synthesize tellurene. Huang et al. grew tellurium thin films on a graphene/6H‐SiC (0001) substrate.^[^ ^190^ ^]^ Chen et al. synthesized an ultra‐thin β‐tellurium layer using highly oriented pyrolytic graphite as a substrate.^[^ ^191^ ^]^

##### Synthesis—Top‐Down Method

Previous studies have proposed that tellurium single crystals are composed of spiral atomic chains held by relatively weak vdWs interactions.^[^ ^186^ ^]^ This structural feature has motivated researchers to investigate the feasibility of top‐down methods for tellurene synthesis. The crystal structure of the tellurium is shown in **Figure** **17**a.^[^ ^28^ ^]^ In this work, high‐quality tripartite tellurium single crystals (Figure 17b) were selected as raw materials, and the freshly cut tellurium crystal surface is directly slid on the clean silicon substrate in the vertical sliding direction of the c‐axis by manual exfoliated method. Figure 17c,d displays OM images of exfoliated tellurene, captured immediately after exfoliation and after three weeks of exposure to air, respectively. In both images, the thinnest regions of the tellurene crystals appeared as darker green and blue contrasts. The two OM images showed no significant morphological changes, demonstrating the stability of tellurene in the air. High‐resolution AFM images are presented on Figure 17e,f. The height profile shows that the thickness of the tellurene is 15 nm. The length of linear band‐like tellurene can reach 50 micrometers. Additionally, the exfoliated tellurene obtained a Raman pattern matching that of bulk tellurium.^[^ ^28^ ^]^ These results confirm that tellurene was obtained by mechanical exfoliation, and its environmental stability is conducive to further exploration of tellurene applications. In addition to mechanical methods, the LPE method has also been successfully used to prepare tellurene. Xie et al. obtained tellurene with a thickness in the range of 5.1 to 6.4 nm by ultrasonic treatment and centrifuge separation of hand‐ground bulk tellurium in IPA solvent.^[^ ^184^ ^]^ HR‐TEM result shows that tellurene has a clear crystal structure, with a lattice spacing of ≈3.2 Å. Another study compared the exfoliation results using water, IPA, and NMP as solvents and evaluated the stability of tellurene nanosheets in solvents. This work suggests that organic solvents (IPA and NMP) can disperse tellurene better, and tellurene has better stability in them.^[^ ^19^ ^]^

##### Properties and Potential Applications of Tellurene—Electronic Devices Applications

Tellurene is considered to have enormous potential in electronic device applications due to its high mobility, excellent switching ratio, and good stability. Wang et al. synthesized tellurene by solution growth method and further fabricated the FETs of tellurene.^[^ ^30^ ^]^ They found that the tellurene‐based FETs showed a switching ratio of up to 10^6^, which means that it has good current modulation capability. At the same time, the relatively high field‐effect mobility (about 700 cm^2^ V^−1^s^−1^) indicates that the good charge‐carrier transport efficiency of tellurene is successfully reflected in the transistor. By reducing the channel length and integrating high‐k dielectrics, transistors with an on‐state current density of more than 1 A mm^−1^ have been achieved, which is one of the highest values of all 2D material transistors to date. The environmental stability of tellurene also allows tellurene‐based FETs to operate unencapsulated in the air for more than two months.^[^ ^30^ ^]^ These characteristics indicate that tellurene‐based FETs have great application prospects in the field of electronic devices, especially in applications requiring high stability, high current density, and high mobility.

The high thermoelectric figure of merit (ZT) value of tellurene shows that it also has a potential application in thermoelectric devices. A report by Liu et al. suggests that tellurene is ideal for thermoelectric generators in wearable devices.^[^ ^192^ ^]^ Tellurene has a ZT value of up to 2.9 (for reference, Bi_2_Te_3_ has a ZT value of 1.3,^[^ ^193^ ^]^ and MoS_2_ has a ZT value of 2.2^[^ ^194^ ^]^), which allows it to convert heat energy into electricity more efficiently. The 2D structure of tellurene has a high specific surface area and ultra‐thin thickness, which improves the efficiency of thermoelectric conversion without adding burden to wearable devices. Tellurene thermoelectric generators can use the heat naturally emitted by the human body, and the low toxicity of tellurene can ensure biosafety when directly contact human body.^[^ ^192^ ^]^ Despite promising performance in both electronics and thermoelectrics, key issues such as growth scalability and long‐term reliability remain to be addressed before tellurene can be widely deployed in real‐world devices.

##### Properties and Potential Applications of Tellurene—Photonic Devices

Zhang et al. found that synthetic tellurene nanosheets exhibit excellent broadband saturation absorption and optical limitation behavior in the laser photon energy range (0.73–2.76 eV) in the near‐infrared to the visible spectrum, which opens up the possibility of tellurene photonic device applications.^[^ ^195^ ^]^ The open‐aperture z‐scan technique was used to investigate the nonlinear absorption characteristics of tellurene. Experimental results show that tellurene nanosheets exhibit optical limitation behavior at high photon energies (2.76, 1.91, and 1.46 eV), namely two‐photon absorption. With the increase of excitation intensity, the transmittance reaches the minimum value at *z* = 0, forming a trough of wave. At 1064 nm (1.17 eV) excitation, the saturation strength of tellurene is the same order of magnitude as that of graphene oxide, but the two‐photon absorption coefficient of tellurene is greater than that of graphene oxide. Tellurene can be used as a saturation absorber in fiber lasers because of its excellent saturation absorption properties under low‐energy photon excitation. In addition, its two‐photon absorption behavior allows it to limit the intensity of light passing through, which has the potential to be used in the protection of sensitive optical devices or eye protection devices.^[^ ^195^ ^]^ Guo et al. investigated the nonlinear optical response of a composite membrane composed of 2D tellurene nanosheets embedded in a polyvinylpyrrolidone (PVP) matrix, using open‐aperture Z‐scan measurements across a broad wavelength range (800–1550 nm).^[^ ^19^ ^]^ The results revealed a consistently high nonlinear absorption coefficient (|β| ∼ 10^−1^ cm GW^−1^) at all tested wavelengths, with peak saturable absorption observed at 1550 nm. Notably, the composite membrane exhibited both large modulation depths and low saturation intensities, confirming its capability as a broadband saturable absorber. The encapsulation of tellurene within the PVP matrix not only stabilized the material against oxidation but also facilitated practical integration into fiber laser systems, where it enabled stable femtosecond pulse generation. Tellurene's exceptional broadband nonlinear optical properties and stable femtosecond pulse generation highlight its promise for ultrafast photonic applications; nonetheless, practical implementation still depends on improving material stability, optimizing composite integration, and validating performance under real‐world laser operating conditions.

### 3.5 Xenes in Other Element Groups

In addition to group III–VI Xenes, several emerging elemental 2D materials namely beryllene, iodinene, molybdenene, and goldene, have recently attracted increasing interest.^[^ ^196^, ^197^, ^198^, ^199^ ^]^ These Xenes exhibit unique lattice symmetries, bonding environments, and synthesis mechanisms that fall outside conventional group‐based trends. This section analyzes their structural features, fabrication methods, and application potentials, with emphasis on how their unconventional electronic configurations and phase selectivity contribute to distinct synthesis challenges and functional opportunities.

#### 3.5.1 Beryllene

##### Synthesis—Top‐Down Method

**Figure 18:**
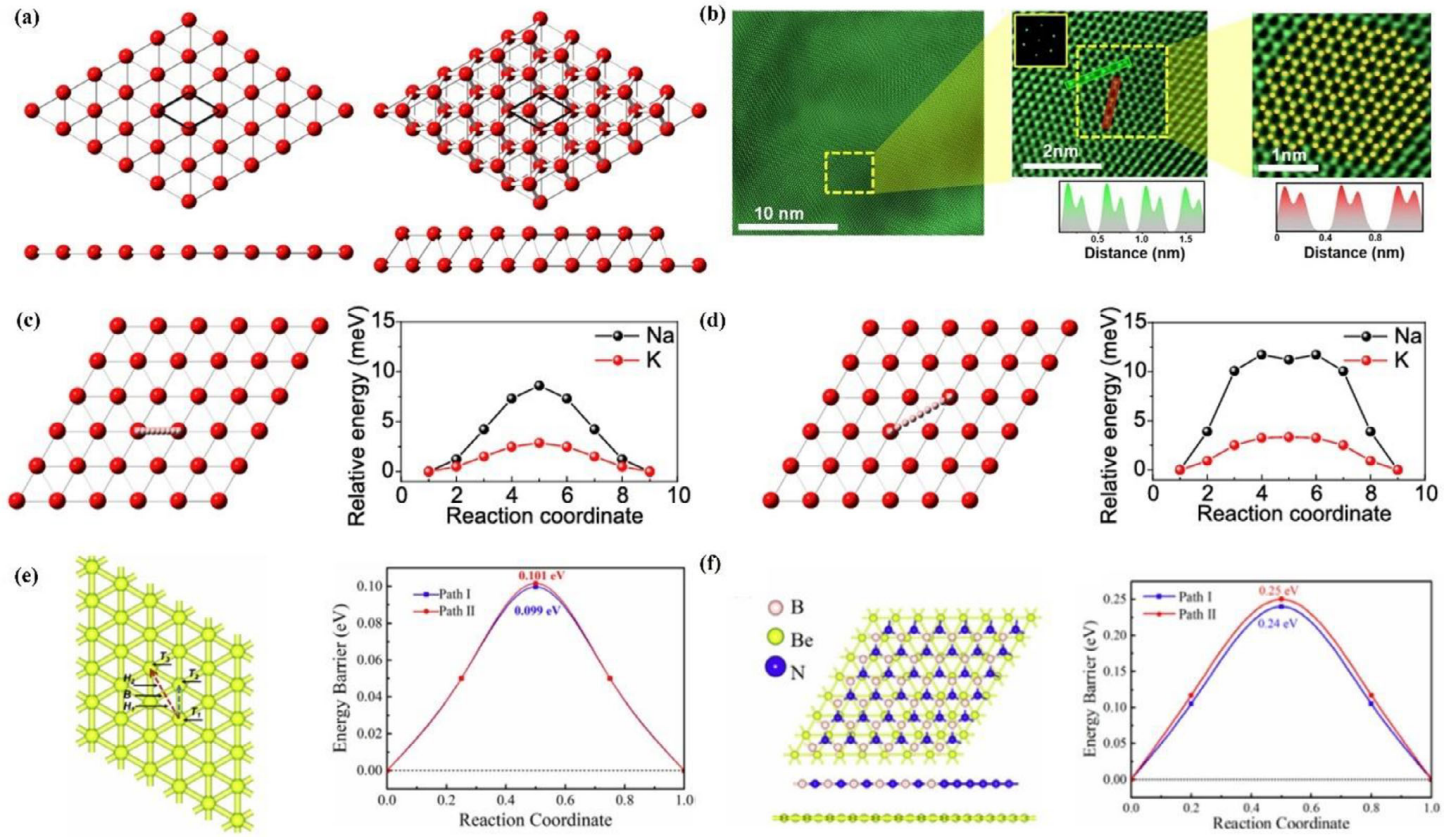
Structural features, atomic resolution imaging, and ion migration pathways of beryllene: a) Top and side views of the α‐beryllene monolayer (left) and β‐beryllene (right); Reproduced with permission.^[^ ^200^ ^]^ 2020, American Chemical Society. b) Large‐scale HR‐TEM images show ordered crystal regions. The magnified area exhibits a distinct hexagonal atomic arrangement, and the line graphs obtained along two lattice directions indicate that the distances between atoms are 1.6 Å (middle) and 1.3 Å (right); Reproduced under the terms of the CC‐BY license.^[^ ^196^ ^]^ Copyright 2023, Sumit Chahal et al; c) T‐B‐T (direct migration route) and d) T‐H‐B‐H‐T (passes through centers of Be triangles) migration pathways and energy barriers for Na⁺ and K⁺ on α‐beryllene; Reproduced with permission.^[^ ^200^ ^]^ Copyright 2020, American Chemical Society; e) Migration pathway for Mg on α‐beryllene and corresponding diffusion barriers; f) Top and side views (left) of the h‐BN/α‐beryllene heterostructure and corresponding diffusion barriers for Mg on h‐BN/α‐beryllene heterostructure; Reproduced with permission.^[^ ^201^ ^]^2023 Royal society of chemistry.

The exploration of beryllene, a 2D elemental beryllium allotrope, has evolved from theoretical prediction to experimental realization. In their landmark theoretical work, Sun et al. proposed two stable forms of 2D beryllene as shown in **Figure** **18**a: α‐beryllene, a single planar layer where each Be atom is sixfold coordinated, and β‐beryllene, composed of two stacked α‐layers with slight interlayer buckling.^[^ ^200^ ^]^ Both phases were predicted to be dynamically stable, thermally robust up to ≈1600 K, and metallic, with ultralow diffusion barriers for Na⁺ and K⁺ ions, and exceptional specific capacities (up to 1487 mAh g^−1^ for Na on α‐beryllene). Chahal et al. reported the first successful synthesis of freestanding beryllene nanosheets via liquid‐phase exfoliation. Metallic beryllium powder was dispersed in DMF and subjected to 20 hours of ultrasonication, followed by centrifugation to isolate monolayer to few‐layer flakes. These exhibited lateral sizes between 0.2 and 4 µm. HR‐TEM (Figure 18b) revealed diverse crystallographic motifs including hexagonal, square, and stripe‐like superstructures, suggestive of multiple stable allotropes and high defect tolerance.^[^ ^196^ ^]^ These polymorphs are stabilized by multi‐center bonding. Specifically, three‐center two‐electron bonds due to beryllium's electron deficiency. Raman spectra revealed two key modes: a crystalline E_2_g mode at ≈451 cm^−1^, and a quantum confinement‐related peak at 614 cm^−1^. XPS confirmed metallic Be⁰ signals, while UV‐Vis spectra showed a sharp absorbance edge consistent with metallic behavior and transparency.

##### Properties and Potential Applications of Beryllene—Energy Storage

Recent theoretical investigations have revealed beryllene, a novel 2D beryllium allotrope, as a highly promising anode material for next‐generation metal‐ion batteries. The α‐ and β‐phases of beryllene exhibit exceptional structural and electronic properties, including high thermal and dynamical stability, metallic conductivity, and strong adsorption capabilities for alkali and alkaline‐earth metal ions. In α‐beryllene, the planar hexagonal structure with sixfold‐coordinated Be atoms and intrinsic metallic band structure (Figure 18a)^[^ ^200^ ^]^ provide a favorable framework for fast electron and ion transport. First‐principles calculations reveal that Na⁺ and K⁺ exhibit ultrafast surface diffusion on α‐beryllene, with energy barriers as low as 9 and 3 meV, respectively, along well‐defined low‐resistance paths (Figure 18c,d).^[^ ^200^ ^]^ In another study on Mg^2^⁺, the migration remains fast and nearly isotropic, with diffusion barriers of ≈0.1 eV in multiple directions and minimal structural distortion (Figure 18e). To ensure environmental stability, a vdWs heterostructure composed of h‐BN on α‐beryllene has been proposed.^[^ ^201^ ^]^ This configuration maintains metallic conductivity and allows Mg adsorption and migration with only a slight increase (≈0.15 eV) in diffusion barrier, demonstrating practical feasibility under encapsulation (Figure 18f). These properties collectively position beryllene as a high‐performance, high‐capacity, and fast‐charging anode material for future Na‐, K‐, and Mg‐ion batteries. The combination of rapid ion diffusion, metallic conductivity, and structural robustness, along with compatibility with encapsulation strategies such as h‐BN layering, highlights the potential of beryllene as an advanced anode material; however, challenges related to experimental verification, large‐scale synthesis, and long‐term safety must still be addressed for practical battery applications.

#### 3.5.2 Iodinene

##### Synthesis—Top‐Down Method

2D iodine, also known as iodinene, has recently emerged as a promising monoelemental material within the Xenes family. Although elemental iodine is more volatile and reactive compared to its group‐VA or group‐VI counterparts, its intrinsic layered molecular crystal structure offers a theoretical basis for 2D exfoliation and stabilization.^[^ ^197^ ^]^ The initial experimental breakthrough was achieved by Qian et al., who successfully exfoliated few‐layer iodinene sheets from bulk iodine crystals using a sonication‐assisted liquid‐phase strategy. The resulting sheets exhibited typical lateral dimensions of several hundred nanometers and a thickness around 1 nm, indicating their ultrathin nature. The authors confirmed the layer‐dependent band structures and surface oxidation behavior through XPS and UV–Vis characterizations.

##### Synthesis—Bottom‐Up Method

**Figure 19:**
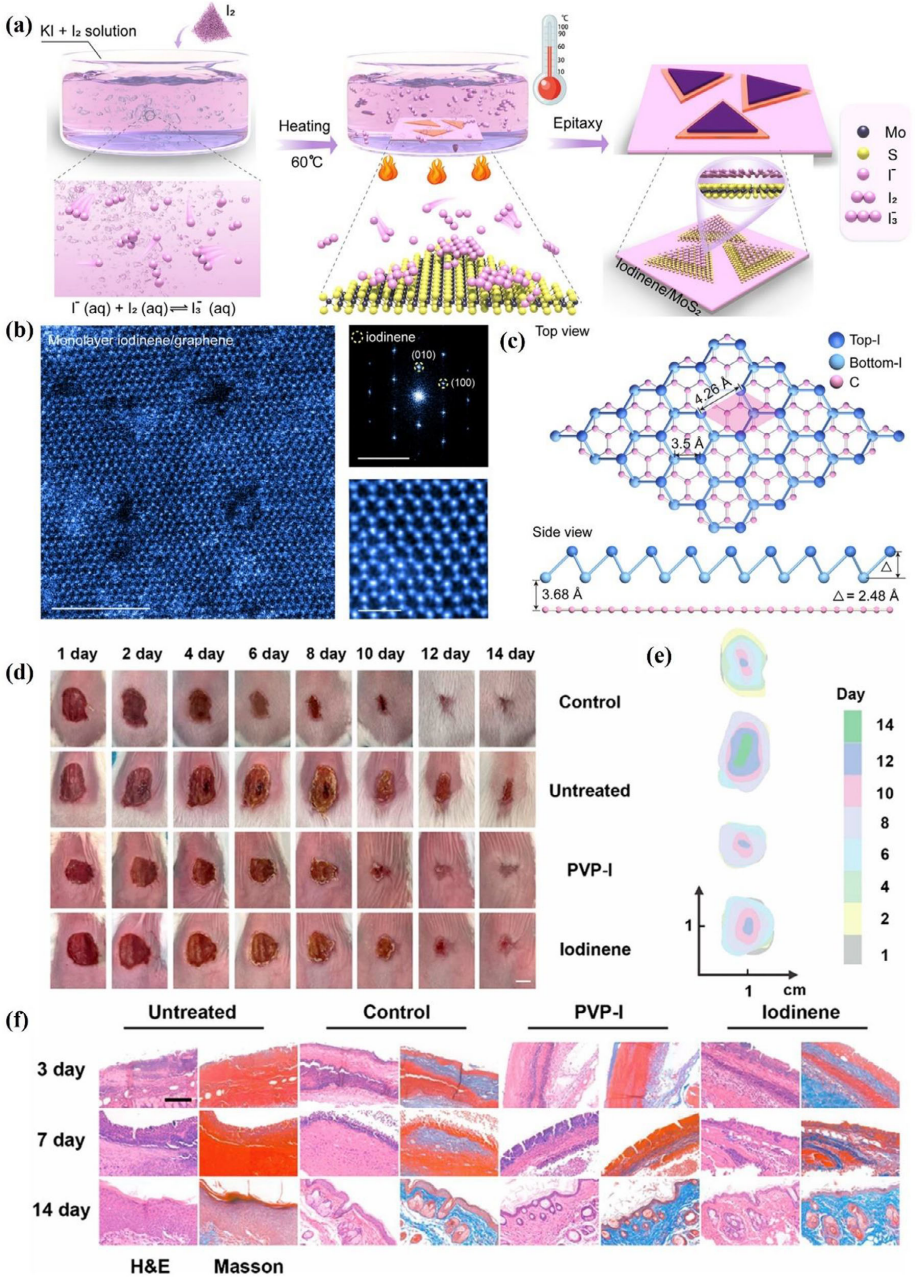
Epitaxial growth, atomic structure, and therapeutic application of monolayer iodinene: a) Schematic of epitaxial growth of monolayer iodinene on a 2D template; b) ADF‐STEM images of the iodinene/graphene heterostructure, including atomic‐resolution visualization of the monolayer with a characteristic honeycomb lattice; corresponding FFT pattern confirms crystallinity; c) Top and side views of the DFT‐optimized model: buckled 1a × 1a iodinene on a √3 × √3 graphene supercell; The pink box marks the unit cell of the iodinene lattice; Reproduced with permission.^[^ ^202^ ^]^ Copyright 2025, Elsevier; d) Representative photographs of infected skin wounds over a 14‐day period following different treatments, illustrating accelerated healing in the iodinene‐treated group; e) Quantitative analysis of wound area reduction corresponding to (d), based on time‐dependent digital tracing of wound edges; f) Histological evaluation of wound tissues collected on days 3, 7, and 14 using Hematoxylin & Eosin and Masson staining, showing enhanced epithelial regeneration, collagen deposition, and reduced inflammation in the iodinene group. Statistical values are presented as mean ± SD (n = 3). Significance was determined by one‐way Analysis of Variance (*p < 0.05, **p < 0.01, ***p < 0.001, ****p < 0.0001); “ns” indicates no significant difference; Reproduced with permission.^[^ ^203^ ^]^ Copyright 2023, American Chemical Society.

Building upon these advances, Luo et al. introduced a template‐guided epitaxial growth method to synthesize monolayer metallic iodinene on MoS_2_ and graphene substrates.^[^ ^202^ ^]^ As shown in **Figure** **19**a, this strategy relies on slow evaporation of iodine under moderate temperatures (∼80 °C), enabling iodine atoms to crystallize on the surface of 2D templates via vdWs interactions. Aberration‐corrected STEM imaging revealed well‐ordered monolayer films of iodinene on graphene with a clear honeycomb symmetry (Figure 19b). The fast Fourier transform confirms a periodic structure with hexagonal diffraction patterns, consistent with the expected crystalline symmetry. High‐resolution ADF‐STEM imaging also resolves the atomic arrangement of iodine atoms within the sheet.

Interestingly, bilayer structures were occasionally observed with clear zigzag edge terminations. These multilayer domains provide a platform to study stacking‐dependent properties. To understand the atomic geometry, DFT calculations confirmed that the energetically favorable configuration adopts a buckled structure (Figure 19c), with a lattice constant of ≈4.26 Å and a vertical buckling height of ∼2.48 Å between the top and bottom iodine sublayers. This buckling enhances structural stability and differentiates the 2D phase from its orthorhombic bulk counterpart.^[^ ^202^ ^]^ The combination of experimental and theoretical data supports the successful stabilization of monolayer iodinene via interfacial charge redistribution and lattice confinement, forming an epitaxial hexagonal superlattice with intriguing electronic features.

##### Properties and Potential Applications of Iodinene—*Biomedical Field*

Biomedical applications were pioneered by You et al., who demonstrated that iodinene nanosheets could undergo a hydrogen peroxide‐triggered transformation into iodinene and hypoiodous acid (HIO), enabling stimuli‐responsive antibacterial activity tailored to infection microenvironments.^[^ ^203^ ^]^ In vivo experiments using *Staphylococcus aureus*‐infected wound models revealed the therapeutic potential of iodinene in promoting wound healing while minimizing systemic toxicity. As shown in Figure 19d, photographic tracking of wound sites from day 0 to day 14 indicated significantly accelerated healing in the iodinene‐treated group compared to PVP‐I and PBS controls. Quantitative analysis of wound closure dynamics (Figure 19e) further confirmed this trend, with the iodinene group exhibiting the most rapid and consistent reduction in wound area over time.

To evaluate tissue‐level regeneration, histological staining was performed on wound sections harvested at days 3, 7, and 14. Figure 19f presents Hematoxylin & Eosin and Masson‐stained images, which show more complete re‐epithelialization, denser collagen deposition, and reduced inflammatory cell infiltration in the iodinene group.^[^ ^203^ ^]^ These results underscore the superior regenerative performance and biocompatibility of iodinene in vivo. Collectively, this study demonstrates that iodinene serves as a smart, H_2_O_2_‐responsive antimicrobial platform, offering precise, on‐demand activation and enhanced wound repair capacity, with promising applications in infection‐associated tissue engineering and wound care. As a stimuli‐responsive antibacterial platform, iodinene enables targeted wound therapy via H_2_O_2_‐triggered activation. Its in vivo efficacy in accelerating healing and minimizing inflammation highlights both therapeutic precision and biocompatibility, though clinical translation will require deeper investigation into long‐term safety and scalable formulation strategies. Demonstrating precise responsiveness to infection‐associated stimuli and strong regenerative outcomes, iodinene represents a promising candidate for advanced wound care applications. Nonetheless, successful clinical implementation will depend on further research into formulation scalability, dosage optimization, and comprehensive long‐term safety evaluation.

##### Properties and Potential Applications of Iodinene—Energy Storage

Energy storage represents a crucial application domain for 2D materials. Qian et al. employed few‐layer iodinene as a cathode material for sodium‐ion batteries (SIBs).^[^ ^197^ ^]^ Compared with bulk molecular iodine, the 2D nanosheet structure of iodinene offered dramatically improved electrochemical kinetics, characterized by faster sodium‐ion diffusion and superior high‐rate performance. Remarkably, even at an ultrahigh current density of 10 A g^−1^, the electrode delivered a reversible capacity of 109.5 mAh g^−1^, with excellent capacity retention after thousands of cycles. These enhancements were attributed to the ultrathin architecture of iodinene, which provides short ion diffusion pathways, enlarged surface area, and a high fraction of pseudocapacitive contribution. First‐principles calculations further revealed a low energy barrier (0.07 eV) for vertical Na⁺ diffusion in few‐layer structures, in contrast to the horizontal diffusion pathways dominant in bulk iodine.^[^ ^197^ ^]^ Leveraging vertical Na⁺ diffusion and pseudocapacitive behavior, few‐layer iodinene delivers outstanding rate capability and cycling stability in SIBs. Its ultrathin structure enables fast ion transport and high surface reactivity, though practical integration will depend on scalable synthesis and long‐term electrode stability under operational conditions. Theoretical and experimental findings position few‐layer iodinene as a highly promising cathode material for sodium‐ion storage, offering fast ion transport and strong pseudocapacitive behavior; yet, its current development remains at the early stage, and further efforts are required to establish scalable synthesis protocols, full‐cell integration strategies, and long‐term cycling reliability under realistic operating conditions.

#### 3.5.3 Molybdenene

##### Synthesis—Bottom‐Up Method

**Figure 20:**
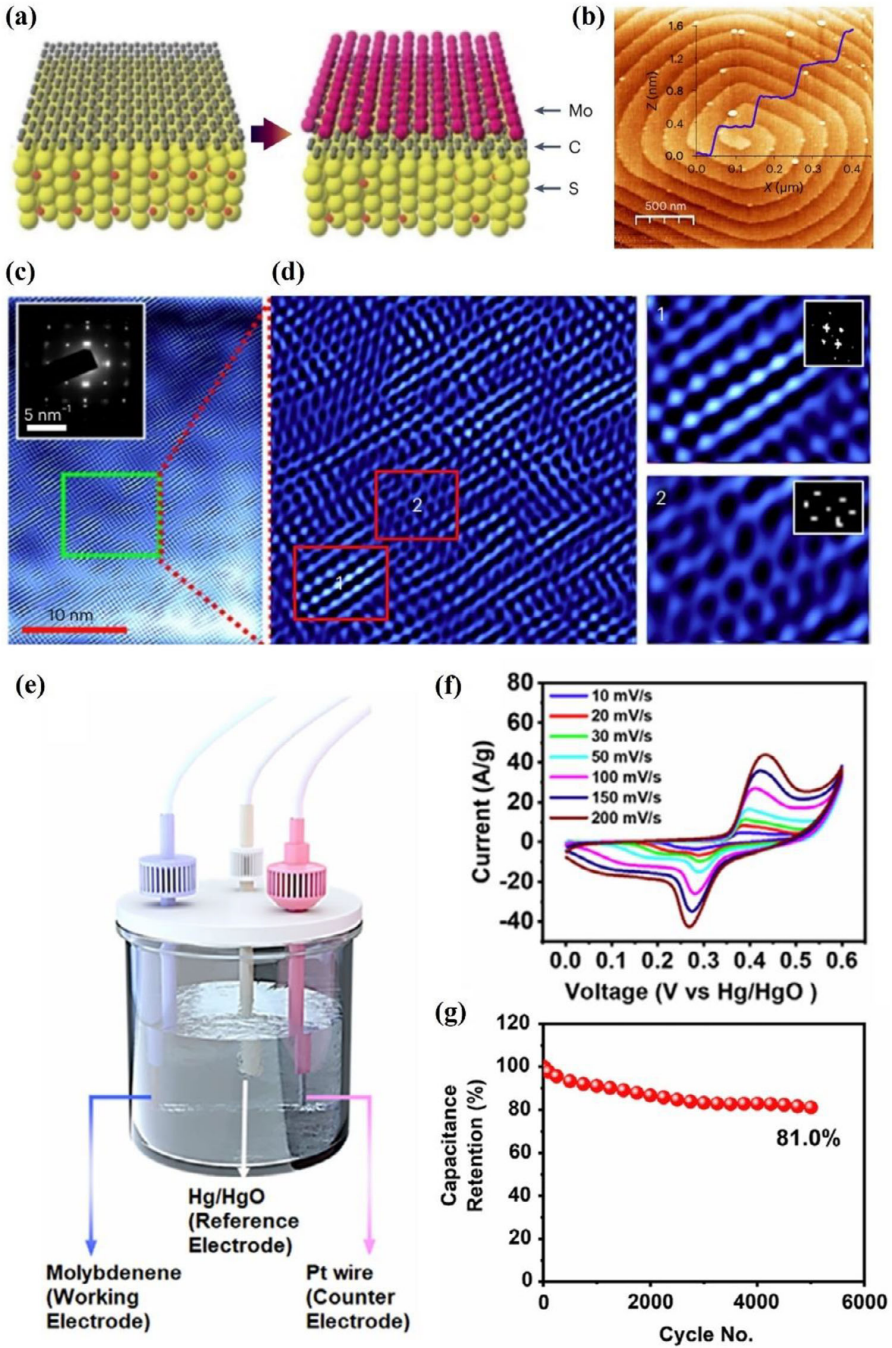
Microwave‐assisted synthesis, structural characterization, and electrochemical performance of molybdenene: a) Schematic of graphene‐assisted microwave synthesis of molybdenene. Microwave‐induced fields break Mo–S bonds, and Mo atoms diffuse through the graphene network to form a 2D sheet. Mo, S, and C atoms are shown in pink, yellow, and grey; b) AFM images showing staircase‐like features (≈0.4 nm steps), indicating screw‐dislocation‐driven monolayer growth; c) HR‐TEM image of the molybdenene top surface; d) Magnified view of selected regions in (c), showing criss‐cross atomic patterns with fourfold symmetry region; Reproduced under the terms of the CC‐BY license.^[^ ^198^ ^]^ Copyright 2023, Tumesh Kumar Sahu et al; e) Schematic illustration of the three‐electrode setup used for electrochemical testing of molybdenene‐based electrodes; f) Cyclic voltammetry curves of molybdenene at scan rates from 10 to 200 mV·s^−1^; g) Capacitance retention of molybdenene over 5000 cycles at 100 mV·s^−1^; Reproduced under the terms of the CC‐BY license.^[^ ^204^ ^]^ Copyright 2025, Ajayan Vinu, Prashant Kumar, Kamlendra Awasthi, et al.

Currently, the synthesis of molybdenene is mainly achieved through bottom‐up strategies. To date, no effective top‐down method has been reported. Unlike vdWs layered materials such as MoS_2_ or graphite, molybdenene lacks a layered precursor and exhibits strong Mo–Mo covalent bonding, rendering top‐down exfoliation approaches ineffective. The first experimental realization of molybdenene was achieved by Sahu et al., who applied microwave irradiation to exfoliate MoS_2_ into a monoelemental metallic sheet.^[^ ^198^ ^]^ As shown in **Figure** **20**a, localized heating and electric fields generated by microwaves resulted in Mo–S bond cleavage and initiated the reorganization of Mo atoms into 2D crystalline layers. The resulting whisker‐like structures exhibited layered surfaces, which were further analyzed by AFM. Notably, Figure 20b displays a staircase‐like topography with step heights of ≈0.4 nm, indicating screw‐dislocation‐driven layer‐by‐layer growth, a typical signature of monolayer formation. HR‐TEM further confirmed the crystallinity and atomic arrangement. As seen in Figure 20c, the molybdenene sheet exhibits a well‐defined periodic lattice structure, and Figure 20d highlights the presence of multiple crystalline domains. In particular, one region shows perpendicular linear arrays of atoms, indicative of a fourfold symmetric lattice, while another region reveals a strained sixfold configuration.^[^ ^198^ ^]^ These features collectively validate the successful bottom‐up formation of free‐standing metallic molybdenene with structural polymorphism at the atomic scale. Building on this strategy, Pandey et al. introduced a modified method in which MoS_2_ was mixed with graphene flakes to enhance microwave absorption.^[^ ^204^ ^]^ Upon pulsed microwave treatment, molybdenene whiskers were formed and subsequently exfoliated via sonication to yield few‐layer nanosheets.

These experimental results are supported by the DFT studies of other related works. Smid et al. performed DFT calculations on four molybdenene polymorphs and identified the buckled hexagonal structure as the only dynamically stable phase, confirmed by the absence of imaginary phonon modes.^[^ ^205^ ^]^ This structure also exhibited metallic behavior with multiple band crossings at the Fermi level. Another work reported by Gusarov et al. reached consistent conclusions, showing that buckling lowers the total energy and enhances thermodynamic stability.^[^ ^206^ ^]^ The buckled hexagonal phase was again the most favorable, while the tetragonal buckled form was identified as a metastable alternative. Electron localization function and Bader analyses revealed delocalized metallic bonding, supporting its high conductivity and structural robustness.

##### Properties and Potential Applications of Molybdenene—Energy Storage

Molybdenene has demonstrated excellent performance as a supercapacitor electrode material, owing to its metallic conductivity, layered morphology, and electrochemical durability. Pandey et al. assessed its performance in a standard three‐electrode system (**Figure** **20**e), where exfoliated molybdenene nanosheets served as the active material.^[^ ^204^ ^]^ The cyclic voltammetry (CV) curves retained a near‐rectangular shape across a range of scan rates (Figure 20f), indicating electric double‐layer capacitance behavior and fast charge transport. In a two‐electrode asymmetric configuration, molybdenene also exhibited stable galvanostatic charge–discharge profiles, with triangular curves maintained across various current densities (Figure 20g). This reflects high coulombic efficiency and structural integrity during cycling. A specific capacitance of 327.78 F·g^−1^ at 10 mV·s^−1^ and 81% capacitance retention after 5000 cycles further underscore its potential as a high‐performance, durable electrode material for energy storage applications.^[^ ^204^ ^]^ Although still in the early research stage, molybdenene has demonstrated highly promising electrochemical properties in the application of supercapacitors, including high specific capacitance, efficient charge transfer, and long‐term cycle stability. These properties have been confirmed both in the three‐electrode configuration and the asymmetric dual‐electrode configuration.

#### 3.5.4 Goldene

##### Synthesis—Top‐Down Method

The development of 2D gold nanostructures has progressed through diverse synthetic strategies, aiming to overcome gold's natural tendency toward isotropic growth and bulk aggregation. Among early efforts, Shin et al. employed an interfacial growth strategy at the oleic acid/water boundary, where the immiscibility between oil and water confined gold nanoparticle growth to two dimensions.^[^ ^207^ ^]^ The reduction of Au^3^⁺ ions by hydroxylamine hydrochloride was locally enhanced by carboxylic acid headgroups on the oleic acid interface, leading to anisotropic lateral growth of hyperbranched gold nanodendrimers. Phase field crystal simulations validated the formation of crystallographically tilted branches under diffusion‐limited conditions. In 2022, Chahal et al. introduced a microwave‐assisted bottom‐up method, in which HAuCl_4_ was rapidly reduced in DMF.^[^ ^208^ ^]^ Although the AFM measurements indicated thicknesses in the range of 0.4–2.6 nm, no structural evidence (e.g., XRD or STEM) was provided to confirm atomic monolayer configuration or the formation of a distinct 2D gold phase. Therefore, these nanosheets were not explicitly identified as goldene by the authors.

**Figure 21:**
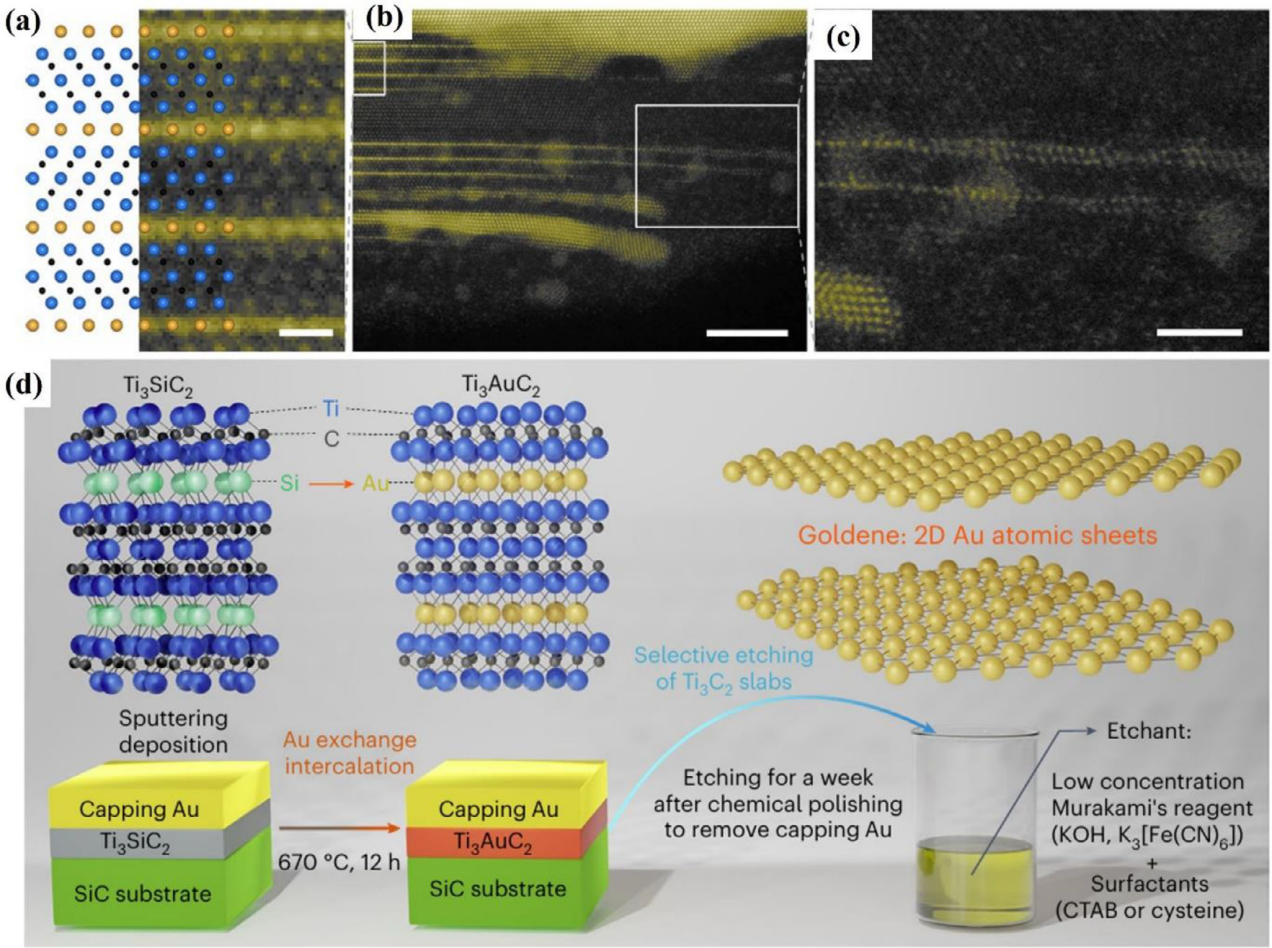
Structural evolution and synthesis mechanism of freestanding goldene: a) Atomic model corresponding to the square‐marked region in (b), showing the arrangement of Au, Ti, and C atoms (yellow, blue, and black, respectively); b) Cross‐sectional high‐resolution scanning transmission electron microscopy (HR‐STEM) image of Ti_3_AuC_2_ after etching with Murakami's reagent and Cetyltrimethylammonium bromide. The left region retains the original Ti_3_AuC_2_ layered structure, while the right side reveals the exfoliated monolayer goldene at the etching front; c) Enlarged HR‐STEM image showing partially curled goldene layers after selective removal of Ti_3_C_2_ slabs, as indicated in (b); d) Schematic illustration of the goldene synthesis process. The Ti_3_SiC_2_ precursor is converted to Ti_3_AuC_2_ via gold intercalation at 670 °C, followed by selective etching of Ti_3_C_2_ using a diluted Murakami's reagent (KOH and K_3_[Fe(CN)_6_]) in the presence of surfactants (Cetyltrimethylammonium bromide or cysteine), leading to the formation of freestanding goldene layers; Reproduced under the terms of the CC‐BY license.^[^ ^199^ ^]^ Copyright 2024, Shun Kashiwaya et al.

A breakthrough was achieved by Kashiwaya et al., who synthesized goldene via top‐down exfoliation from a layered MAX phase, Ti_3_AuC_2_.^[^ ^199^ ^]^ The process involved selective removal of the Ti and C atomic layers using a diluted Murakami's reagent in the presence of surfactants such as cetyltrimethylammonium bromide or cysteine, enabling the release of a single‐atom‐thick gold layer. The structural features of the system were revealed through advanced electron microscopy and schematic modeling. In the initial Ti_3_AuC_2_ structure, gold atoms occupy the internal layers of the MAX lattice, as illustrated by an atomic model highlighting the layered configuration and elemental positioning (Figure 21a). Upon partial etching, high‐resolution STEM imaging captured a distinct transition zone, where the intact Ti_3_AuC_2_ on the left gives way to the emergent monolayer goldene on the right (Figure 21b), clearly visualizing the exfoliation front. Further magnified images revealed that the resulting goldene sheets retained their atomically thin nature while exhibiting slight out‐of‐plane curling, confirming their freestanding character and intrinsic flexibility (Figure 21c). Figure 21d illustrates the process of embedding gold into the Ti_3_SiC_2_ precursor, converting it into Ti_3_AuC_2_ at high temperatures, and subsequently separating out the gold film through chemical exfoliation.^[^ ^199^ ^]^ Notably, the strong in‐plane Au–Au bonding ensured structural integrity throughout exfoliation, distinguishing this approach from previous methods reliant on substrate confinement or multi‐atom‐thick assemblies. Building upon this experimental foundation, Mortazavi et al. conducted a comprehensive theoretical investigation using DFT and machine‐learned interatomic potentials, which validated the mechanical rigidity, low lattice thermal conductivity (∼10 W/m·K), and robust metallic character of goldene under strain and elevated temperatures, thereby affirming its viability as a stable and freestanding 2D metallic system.^[^ ^209^ ^]^

##### Properties and Potential Applications of Goldene—*Hydrogen Sensing and Adsorption*

To date, the application‐oriented research on goldene remains limited to theoretical studies, with no experimental reports demonstrating functional use. Among these, hydrogen adsorption has emerged as a key direction. A first‐principles study by Sheremetyeva and Meunier revealed that hydrogen atoms can stably adsorb onto goldene's hollow sites, with adsorption energies ranging from −0.33 to −0.42 eV for surface coverages between 1/9 and 4/9.^[^ ^210^ ^]^ The system maintains dynamic and mechanical stability under partial hydrogenation, and the adsorption induces distinct features in both the local density of states and simulated STM contrast, suggesting potential for hydrogen sensing. Moreover, vibrational analysis identified a pronounced Au–H stretching mode at 1065 cm^−1^, providing a spectroscopic signature for detection. Although high coverage reduces adsorption strength, the overall interaction remains sufficient for surface‐functional applications. Although the exploration of the application fields of goldene is still at the stage of theoretical research, these results still indicate that goldene may serve as a chemically stable and spectrally active two‐dimensional platform for hydrogen detection, mapping, or anti‐corrosion surface coating, and it is worthy of experimental research in the future.

## 4 Comparative Insights, Challenges, and Outlook for Xenes

The development of Xenes materials has undergone a significant transformation, driven by both the intrinsic chemistry of the constituent elements and the shifting demands of practical applications. A cross‐family comparison reveals a clear gradient in synthetic complexity and structural stability, reflecting a broader trend in the evolution of two‐dimensional elemental materials.^[^ ^2^, ^6^, ^9^ ^]^

Generally, Xenes derived from lighter elements (such as boronene and silicene located in the IIIA and IVA groups) require extremely complex growth conditions, including extremely high vacuum conditions and carefully selected metal substrates, in order to stabilize their monolayer structure.^[^ ^8^, ^10^, ^17^ ^]^ These conditions, while enabling precise atomic control, impose severe limitations on scalability and transferability.^[^ ^78^ ^]^ In contrast, heavier group elements, particularly those in groups VA and VIA, give rise to layered compounds such as phosphorene and tellurene, which can be exfoliated through top‐down methods like LPE, allowing for larger‐scale and more accessible synthesis.^[^ ^126^, ^183^, ^184^ ^]^ This transition from substrate‐constrained epitaxy to solution‐processable synthesis also reflects a shift in design philosophy. Initially, the field focused on the pursuit of structurally exotic Xenes with unique electronic or topological properties, even at the expense of yield and stability.^[^ ^4^, ^13^, ^127^ ^]^ However, the emphasis has expanded to include large‐area fabrication, environmental robustness, and compatibility with device integration.^[^ ^30^, ^35^ ^]^ Notably, while early‐generation Xenes boast superior structural precision, such as the anisotropic striped phases of borophene, their synthesis is limited in scale. Conversely, tellurene, despite being synthesized through a relatively crude process, can form stable and freestanding ribbons with scalable dimensions.^[^ ^35^ ^]^

This family‐based comparison reveals a clear trend: as the transition occurs from the earlier Xenes (such as borophene and silicene) to heavier element categories of Xenes (such as tellurene), the synthetic routes become more flexible, independent structures become easier to achieve, and large‐scale manufacturing becomes more feasible. However, behind this apparent simplification of synthesis lies a deeper and persistent challenge: the successful path to realizing Xenes still heavily relies on the cumbersome process of combining density functional theory‐based predictions with repeated experimental verifications.^[^ ^43^, ^57^ ^]^ To address this challenge, researchers typically employ a combined approach of DFT simulation and experimental trial‐and‐error to determine the feasible synthesis routes for new Xenes. Although this integrated method has successfully achieved several previously envisioned monoelemental two‐dimensional materials, it often requires a significant amount of manpower, time, and resources. The design space of Xenes is extremely broad, covering not only different element candidates but also various substrates, precursors, temperatures, and reaction environments. Therefore, accelerating this discovery process requires more efficient tools. Among them, data‐driven methods and machine learning frameworks have emerged as promising candidate solutions.^[^ ^211^, ^212^, ^213^ ^]^ Song et al. proposed a representative direction that combines large language model–based learning with an efficient text representation of crystal structures to identify structural subspaces with high synthetic possibility.^[^ ^220^ ^]^ By learning from known experimental structures, their framework can efficiently predict which crystal structure are most likely to be successfully synthesized, even if thermodynamic stability cannot be guaranteed. This addresses a long‐standing limitation of energy‐driven crystal structure prediction, which often fails to capture kinetically accessible metastable states. In parallel, Wang et al. demonstrated that machine learning can also predict key electronic properties of Xenes, such as band gaps under strain with remarkable accuracy using compact datasets.^[^ ^20^ ^]^ Their decision tree regression model, trained on DFT‐derived descriptors (e.g., lattice constants, valence, electronegativity) can accurately reproduce the bandgap of tellurene and generalize well to other strained 2D materials. This kind of property‐predictive machine learning model can greatly reduce the computational cost of screening new Xenes for electronic and optoelectronic applications. These methods may predict the synthesis window, optimize reaction parameters, and even propose new stable strategies, thereby reducing reliance on lengthy DFT experimental cycles.

However, for Xenes synthesis prediction, there are still some key limitations in machine learning and deep learning methods. Firstly, the training datasets often favor structures that are thermodynamically stable under specific conditions, making it difficult for the model to understand the kinetic accessible or metastable phases. Secondly, many machine learning models are like “black boxes” and lack interpretability. This is a problem for experimentalists who need clear chemical insights to guide synthesis. Thirdly, most models rely on static structural features and cannot capture key kinetic factors such as nucleation barriers, substrate effects, or diffusion kinetics, which are crucial for accurately predicting the growth paths of two‐dimensional materials. To address these limitations, some strategies can be referred to. For instance, transfer learning enables pre‐trained models trained on large databases to adapt to specific Xene systems with limited data. Goodall and Lee demonstrated that such models, using only stoichiometry, can accurately predict formation energies, which helps alleviate the problem of data scarcity and the bias towards known phases.^[^ ^214^ ^]^ At the same time, physical information neural networks embed symmetries and energy constraints into the model architecture, improving the extrapolation effect and solving the problem of the lack of dynamical awareness in traditional machine learning models.^[^ ^215^ ^]^ Although these methods are promising, they still need to be more closely integrated with experimental feedback and dynamical modeling to completely overcome the “real gap” between predictions and actual Xene synthesis.

On the other hand, even for those Xenes that have been successfully synthesized, a persistent challenge lies in their stability under ambient conditions.^[^ ^216^ ^]^ Many Xenes are prone to degradation through oxidation, structural reconstruction, or substrate‐dependent instability once removed from vacuum or protective environments.^[^ ^57^ ^]^ For instance, exposure to air, moisture, and light can lead to rapid degradation of the structure and loss of electronic functionality.^[^ ^57^, ^125^ ^]^ A notable example is 2D BKP. This material will oxidize within a few hours when exposed to ambient air, forming surface bubbles and phosphorus oxides, which reduces its electrical performance.^[^ ^217^ ^]^ Water further accelerates this process through a polarization effect that enhances the electron‐accepting ability of oxygen. This significantly limits their applicability in practical devices, where environmental robustness is a prerequisite. Strategies such as encapsulation, functionalization, or the construction of heterostructures have shown promise. Recent studies have begun to employ encapsulation strategies to enhance the stability of specific Xenes. For example, Martella et al. demonstrated that encapsulating silicene with an Al_2_O_3_ layer and transferring it onto flexible substrates significantly improves its environmental robustness while enabling reversible strain‐responsiveness.^[^ ^92^ ^]^ This approach mitigates substrate‐induced electronic perturbations and facilitates integration of Xenes into flexible and strain‐sensitive electronic applications.

Beyond encapsulation, the formation of Xene‐based heterostructures provides another effective route to enhance material stability.^[^ ^218^ ^]^ In lateral heterostructures, such as the germanene–stanene junctions reported by Ogikubo et al., the atomically sharp interfaces suppress elemental intermixing and minimize defect formation, thereby preserving the intrinsic electronic structure of each domain.^[^ ^18^ ^]^ Their work reveals that carefully engineered in‐plane junctions maintain structural order and prevent 3D islanding or amorphization, both of which are common degradation pathways in monolayer Xenes. Similarly, Dhungana et al. demonstrated that vertical stacking of silicene and stanene layers leads to enhanced air stability, especially when combined with encapsulation.^[^ ^219^ ^]^ The heterointerface reduces substrate‐induced strain and charge transfer, allowing each layer to retain its structural identity and electronic functionality over time. A representative example of such stability‐enhancing heterostructure engineering is the borophene–graphene system demonstrated by Liu and Hersam.^[^ ^220^ ^]^ Through bottom‐up synthesis under ultrahigh vacuum, they achieved both lateral and vertical heterointegration of borophene and graphene. Notably, the lateral heterointerface exhibited near‐atomic sharpness despite the substantial mismatch in lattice symmetry and periodicity. This sharp interface is enabled by boron's diverse bonding configurations and multicenter bonding characteristics. Moreover, vertical stacking was realized via boron intercalation beneath pre‐grown graphene, leading to rotationally commensurate heterostructures that electronically decouple the graphene from the metal substrate and reduce substrate‐induced perturbations. These observations affirm that borophene's structural flexibility supports the formation of structurally coherent and electronically clean heterointerfaces, even with dissimilar 2D materials These findings highlight how heterostructure engineering can mitigate environmental sensitivity and unlock practical applications of Xenes in nanoelectronics.

However, despite these advances, Xenes‐based heterostructures still face numerous challenges. A key challenge lies in the fact that many Xenes materials (such as silicene, germanene, and boronene) lack natural vdWs gaps, resulting in strong interface coupling. This makes it more difficult to form atomically sharp and chemically inert interfaces than in traditional two‐dimensional heterostructures (such as graphene/hexagonal boron nitride). Additionally, the sensitivity of Xenes synthesis to substrate crystal structure, surface cleanliness, and thermal stability makes it difficult to reproducibly fabricate high‐quality heterostructures at wafer‐scale areas. These issues are further compounded by the lack of mature transfer and synthesis technologies, as many Xenes materials must be synthesized and stored in ultra‐high vacuum or inert atmospheres to avoid environmental reactivity. To overcome these limitations, future research may rely on the integration of precise substrate engineering and dynamic interface control. The use of pre‐patterned catalytic templates or chemically functionalized substrates can enable selective nucleation and orientation locking, thereby suppressing interface defects. Meanwhile, interface passivation strategies can reduce unwanted diffusion while maintaining epitaxial alignment. Machine learning models trained on high‐throughput simulation and experimental databases can also provide data‐driven methods for navigating the complex phase space of Xene growth and heterostructure formation.

## 5 Conclusions

In summary, this review provides a comprehensive overview of the progress made in the synthesis and stabilization of Xenes, systematically classified by element group (III, IV, V, VI, and others). Although Group III Xenes (such as borophene) exhibit promising anisotropic properties, their extreme instability at room temperature and pressure poses significant challenges for synthesis. Group IV Xenes (such as silicene and germanene) typically require synthesis under ultra‐high vacuum conditions using epitaxial templates, limiting their scalability. Group V Xenes benefit from having layered bulk phases suitable for exfoliation, but face issues of rapid oxidation and degradation. Group VI Xenes exhibit improved environmental stability, but controlling phase purity and morphology remains challenging. Additionally, emerging non‐traditional Xenes introduce complex electronic behavior, necessitating further synthetic innovations. Despite progress in both top‐down and bottom‐up preparation methods, issues such as low yields, poor air stability, and substrate dependency persist across all categories. Recent advancements in encapsulation technology, heterostructure design, and machine learning‐guided synthesis demonstrate significant potential for addressing these challenges. In the future, by integrating in situ characterization, theoretical modeling, and high‐throughput experiments, controllable and scalable production of Xenes will be achieved. Such interdisciplinary efforts are expected to unlock the full potential of Xenes in electronics, energy storage, catalysis, and other fields.

## Conflict of Interest

The authors declare no conflict of interest.
